# The role of transposable elements in functional evolution of amphioxus genome: the case of opsin gene family

**DOI:** 10.1038/s41598-018-20683-9

**Published:** 2018-02-06

**Authors:** Chrysoula N. Pantzartzi, Jiri Pergner, Zbynek Kozmik

**Affiliations:** 1 Laboratory of Eye Biology, Institute of Molecular Genetics of the ASCR, v.v.i., Division BIOCEV, Prumyslová 595, 252 50 Vestec, Czech Republic; 2 Department of Transcriptional Regulation, Institute of Molecular Genetics of the ASCR, v.v.i., Videnska 1083, 14220 Prague 4, Czech Republic

## Abstract

Transposable elements (TEs) are able to jump to new locations (transposition) in the genome, usually after replication. They constitute the so-called selfish or junk DNA and take over large proportions of some genomes. Due to their ability to move around they can change the DNA landscape of genomes and are therefore a rich source of innovation in genes and gene regulation. Surge of sequence data in the past years has significantly facilitated large scale comparative studies. Cephalochordates have been regarded as a useful proxy to ancestral chordate condition partially due to the comparatively slow evolutionary rate at morphological and genomic level. In this study, we used opsin gene family from three *Branchiostoma* species as a window into cephalochordate genome evolution. We compared opsin complements in terms of family size, gene structure and sequence allowing us to identify gene duplication and gene loss events. Furthermore, analysis of the opsin containing genomic loci showed that they are populated by TEs. In summary, we provide evidence of the way transposable elements may have contributed to the evolution of opsin gene family and to the shaping of cephalochordate genomes in general.

## Introduction

Transposable elements (TEs) are complicated biological entities able to replicate and jump to new locations (transposition) in the genome. Rather simple models have been defined to study their dynamics^1^, while their classification is also problematic. The first TE-classification system^2^, distinguishes two classes of TEs, based on the transposition intermediate: RNA (class I or retrotransposons) and DNA (class II or DNA transposons), which follow a “copy-and-paste” and “cut-and-paste” mechanism, respectively. This system was later modified in order to include bacterial, non-autonomous TEs (such as the Miniature Inverted Repeat Transposable Elements - MITEs) and other types of TEs that couldn’t fall in any of these two categories. Curcio and Derbyshire^3^ categorized transposons according to the way they move, determined by their transposase proteins. A hierarchical classification system for eukaryotic TEs has been proposed by Wicker, *et al*.^4^, which takes into account not only the replication strategy but also the structure of the encoded proteins and of the non-coding domains, the presence and size of the target site duplication (TSD) and even some phylogenetic data.

It was long ago speculated that TEs can “control the time and type of activity of individual genes”^5^, or in other words they play key role in a variety of gene regulatory networks and lately there is accumulating information in favor of this theory (revised by Chuong *et al*.^6^ and Bourque^7^). This can be achieved either by the insertion of TEs in the proximity of genes and consequently the generation of new regulatory elements^7^ or the emergence of new regulatory proteins^8^. In fact, TEs occupy a large proportion of the regulatory control regions (revised by Feschotte^8^). On one hand, TEs alter gene expression (activate or inactivate genes); on the other hand, they promote inversions and deletions of chromosomal DNA, they can create new genes (or exons), or serve as illegitimate recombination hotspots. Consequently, they contribute to the shaping of the genome’s architecture, its evolution and the emergence of genetic innovations^9–12^. TE-associated chromosomal rearrangements can be driven by two mechanisms, in particular via homologous recombination^13^ or by an alternative transposition process^14^.

TEs are main components of eukaryote and prokaryote genomes and they are known to occupy large portions of vertebrate, invertebrate and plant genomes in particular^15–19^. Long-terminal repeat retrotransposons (LTRs) are the predominant order of TEs in plants^20^, whereas the Non-LTR TEs are the most commonly encountered in the human genome^21^ and *Alu* repetitive elements, in particular, are known to generate deletions, duplications and complex genomic rearrangements^22^.

The subphylum Cephalochordata, a.k.a. amphioxus or lancelets, have been regarded as a key animal group for understanding the origin of vertebrates, and a useful proxy to the ancestral chordate condition. This is in part due to the presumed slow evolutionary rate within the cephalochordate lineage both at the morphological and the genomic level. Cephalochordata are comprised of the three genera, namely *Branchiostoma*, *Asymmetron* and *Epigonychtys*^23^. It was recently found that Cephalochordata preserve a high TE diversity in comparison to modern vertebrates^24^. In fact, a comparative analysis of TEs in various genomes has revealed that they constitute 28% of *B. floridae* genome^25^. Amphioxus TEs belong to more than 30 superfamilies, which are highly heterogeneous as generally none of their members are drastically more abundant than others, and none of the TEs seems to have suffered any massive expansion^26^. The phylogenetic relationship within the extant amphioxus lineage was investigated^27^ providing divergence time estimates and suggesting a rather recent diversification within *Branchiostoma* genus, with divergence time similar e.g. to that between rodents belonging to *Muridae* family (mouse and rat)^28^. Whole genome comparative study of *B. belcheri* and *B. floridae* indicated high rate of proteome diversification^24^, which might however be explained at least in some cases by the gene prediction errors^29^.

In order to provide an insight into the possible role of TEs in cephalochordate genome evolution we focused on the opsin gene family, a member of the G-Protein Coupled Receptor (GPCR) gene superfamily. Opsins play crucial role in light detection in animals and their number differs significantly among species, with no apparent correlation to the overall level of body plan sophistication. Opsins classification, interfamily relationships and evolution of animal vision have been studied extensively^30–39^. Opsins can be roughly clustered into four major groups, namely the ciliary opsins expressed in ciliary photoreceptors (C-type), the rhabdomeric opsins expressed in rhabdomeric photoreceptors (R-type), the Group 4 opsins, and the Cnidarian opsins. Members of the three major groups were recently identified in the European lancelet^40^, whereas similar studies in the past were focused on the opsin complements of the Florida and Chinese lancelets^41–43^. By using manually curated and experimentally confirmed opsin complement of three *Branchiostoma* species, namely *B. lanceolatum* (Pallas 1774), *B. floridae* (Hubbs 1922) and *B. belcheri* (Gray 1847), we have identified gene duplication and loss events. Extrapolating from opsin gene family as an example, we try to address the question of how transposable elements may have been involved in the gene gain/losses and shaping of the *Branchiostoma* genus genome.

## Materials and Methods

### Gene Prediction, alignments, synteny and phylogenetic analysis

We analyzed both available *Branchiostoma floridae* genome assemblies, i.e. v1.0 through JGI, where two haplotypes are present (http://genome.jgi.doe.gov/Brafl1/Brafl1.home.html) and v2.0 through NCBI (http://www.ncbi.nlm.nih.gov/assembly/GCF_000003815.1/), from which most of the allelic scaffolds have been eliminated and is therefore a non-redundant mosaic of v1.0. All previously annotated opsin genes^41^ were validated through BLAST, Genscan^44^ and SpliceView^45^ analyses. In order to detect putative opsin homologs that were not previously reported, we conducted extensive keyword and BLAST searches. Newly identified opsin containing genomic loci were subjected to Genscan and SpliceView for *de novo* gene prediction. In the case of discrepancies between database gene models and our *in silico* analysis, PCR amplification of the “suspicious” regions was performed, followed by cloning and sequencing (see paragraph “Cloning and Sequencing of Opsin Gene Fragments/Transcripts”). Additionally, we thoroughly queried the *B. belcheri *HapV2(v7h2) and the v18h27.r3_ref_genome assemblies, available at the Chinese Lancelet (Amphioxus) Genome Sequencing project webpage (http://genome.bucm.edu.cn/lancelet/), applying both keyword and BLAST searches. In order to investigate the phylogenetic relationships of previously annotated and newly identified amphioxus opsins and thus establish orthology of opsin genes, a Maximum Likelihood tree was constructed according to Pantzartzi *et al*.^40^. The same dataset was used and it was enriched with *B. floridae* and *B. belcheri* sequences (Supplementary File 1 and Table 1). For each opsin gene, orthologs from the three *Branchiostoma* species were aligned using ClustalO^46^ and visualized using BoxShade. In the case of orthologs absent from one or two species, we used Circoletto^47^, in order to investigate synteny conservation and visualize sequence similarity among syntenic scaffolds from the *Branchiostoma* species. E-value for the BLAST run was set to e^−20^.

### Transposable Elements Analysis

Genomic scaffolds containing opsins and those expected to contain opsin genes based on synteny analyses were screened for repetitive elements using Censor^48^ in the RepBase database^49^. NCBI Accession numbers for *B. floridae* scaffolds used are NW_003101565 (Bf_scaffold6), NW_003101418 (Bf_scaffold_187), NW_003101537 (Bf_scaffold_36), NW_003101507 (Bf_scaffold_98) and NW_003101409 (Bf_scaffold_196). The genomic regions used were: Bf_scaffold_6: 305,868–729,662 or 305,868–547140 (Comparison of Narrow Regions, CNR); Bf_scaffold_187: 4,135,366–4,628,754 or 4,135,366–4,378,895 (CNR); Bl_Sc0000005: 5,300,000–7,300,000 or 6,885,201–7,300,000 (CNR); Bb_scaffold48: 1–2,523,832 or 1,200,000–2,523,832 (CNR); Bf_scaffold_36: 4,567,754–4,488,902, Bf_scaffold_98: 4,107,000–4,213,900, Bl_Sc0000154: 143,384–219,100, Bl_Sc0000040: 850,000–1,050,000, Bb_Sc0000263: 1–200,000; Bb_scaffold123: 447,402–528,601; Bf_scaffold_196: 2,792,247–2,817,466, Bl_Sc0000011: 2,118,981–2,146,160; Bb_Sc0000116: 763,100–794,099.

### Animal Collection

*B. floridae* adults were collected in Old Tampa Bay (Florida, USA, no permission required for amphioxus collection). Housing of animals and *in vivo* experiments in the present study were performed in accordance with guidelines established by the Institute of Molecular Genetics and in compliance with national guidelines (ID#12135/2010–17210). All animal works were also conducted according to the National Institute of Health standards as underlined by the “Guide for Care and Use of Laboratory Animals’’. Gametes were obtained and embryos raised, as previously described^50^. Staging of all collected embryos was performed according to Hirakow and Kajita^51^, specimens from late neurula (N3), larvae (L1–L3) and adult stage were collected and frozen in RNAlater® Stabilization Solution (ThermoFisher Scientific), under light conditions.

### RNA Isolation/cDNA Preparation

Total RNA was isolated from *B. floridae* embryos stored in RNAlater® Stabilization Solution using the Trizol reagent (Ambion). To avoid genomic DNA contamination, isolated RNA was treated with DNaseI and purified on RNeasy Mini Kit (Qiagen) column. Random-primed cDNA was prepared from 250ng of RNA in a 20 μl reaction using SuperScript VILO cDNA Synthesis kit (Invitrogen).

### Cloning and Sequencing of Opsin Gene Fragments/Transcripts

For validation of the *in silico* predicted gene models, cloning and sequencing of opsin gene fragments and complete transcripts from *B. floridae* was performed, according to Pantzartzi *et al*.^40^. Primers used are included in Supplementary Table 2.

### qRT-PCR

Primers used are provided in Supplementary Table 2. Experiments and analysis of results were performed according to Pantzartzi *et al*.^40^. TBP was used as the housekeeping gene.

## Results

### Identification, classification and genome organization of opsin genes in the *Branchiostoma* genus

We initially performed a thorough comparative analysis of the opsin gene repertoires of three cephalochordate species. We used the recently reported genes from *B. lanceolatum*^40^ together with previously reported genes from *B. floridae* and *B. belcheri*^41–43^ many of which had to be re-predicted and some were *de novo* identified in the current study (Supplementary Table 1). Final transcripts and encoded proteins for newly characterized and modified opsins from *B. floridae* and *B. belcheri* as well as details on gene organization and genomic location are provided in Supplementary File 1. Orthology of identified genes was validated by synteny and phylogenetic analysis (Supplementary Fig. 1). The alignments of orthologs for each opsin gene from the three *Branchiostoma* species are provided in Supplementary File 1. Orthologs have the same number of exons; the sole exceptions are *op7* and *op20*. Orthologous exons have almost identical size, however, pronounced changes are observed in the size of the last exon. Furthermore, there is a great similarity among orthologs in terms of sequence, with the C-terminus being the most variable. Evidently, opsin genes are spread over 16 genomic regions (scaffolds) in *B. floridae* and 14 in *B. belcheri* (Supplementary Fig. 2). Phylogenetic analysis (Supplementary Fig. 1) in combination with the arrangement of opsin genes in the genomes of the three species (Supplementary Fig. 2) supports the fact that the majority of opsin genes are represented by an ortholog in all three species (Table 1). This is not the case for *op6*, *op12b*, *op13b*, and *op17b*, which seem to be the result of a gene duplication.

**Table 1:** Opsin repertoire in the *Branchiostoma* genus.

		*B. lanceolatum*	*B. floridae*	*B. belcheri*
C-type opsins	***op1***	+	+	+
	***op2***	+	+	+
	***op3***	+	+	+
	***op4***	+	+	+
	***op5***	+	+	+
Neuropsins	***op6***	−	+	−
	***op7***	+	+	+
	***op8***	+	+	+
Go opsins	***op9***	+	+	+
	***op10***	+	+	+
	***op11***	+	+	+
	***op12a***	+	+	+
	***op12b***	−	−	+
	***op13a***	+	+	+
	***op13b***	+	−	−
Peropsins	***op14***	+	+	+
Melanopsins	***op15***	+	+	+
Amphiop6	***op16***	+	+	+
	***op17a***	+	+	−
	***op17b***	+	−	−
	***op18***	+	+	+
	***op19***	+	+	+
	***op20***	+	+	+
	***op21***	+	+	+
*TOTAL*		**21+1pseudo**	21	20

Note: Numbering of genes is based on Pantzartzi *et al*.^40^.

We further analyzed the opsin expression pattern across different developmental stages (Supplementary Fig. 3) of *B. floridae*. Onset of several opsin genes expression starts at L1 stage, in which frontal eye and lamellar body (ciliary photoreceptive organs) start to develop. In agreement with *B. lanceolatum*^40^, the majority of the *B. floridae* opsins show most predominant expression in L2/3 stages, where all of the known amphioxus photoreceptor organs are differentiated. Nevertheless, differences are observed between the two species in regard to the onset of expression of *op13a*. Interestingly, *op6*, a gene detected only in *B. floridae*, follows a distinct pattern in regard to the other two neuropsins (i.e. *op7* and *op8*), for which expression patterns are the same for both *B. floridae and B. lanceolatum*.

### Transposable elements and opsin genes in the *Branchiostoma* genus

Differences have been noted among the three *Branchiostoma* species in regard both to the structure and the number of opsin genes (Table 1 and Supplementary File 1). Since transposable elements (TEs) have been vastly implicated in gene structure alteration as well as gene duplications and losses, we scanned scaffolds containing altered genes against RepBase to locate TEs populating these regions; for opsin orthologs that are absent from one or two *Branchiostoma* species (Table 1), we found the syntenic scaffolds and also scanned them against RepBase.

The beginning of forth exon of *Bl_op2* is occupied by small repeated sequences, a fact that leads to elongation of the third cytoplasmic loop (Supplementary File 1). Noticeably, the fifth intron of *Bl_op8* highly resembles a satellite locus from *Salmo salar* (SAT-11_SSa in RepBase). In fact, the beginning of the last exon is one of the repeat units. It is also worth mentioning that the last exon of *Bl_op16* is longer in size than the respective exons from the *B. floridae* and *B. belcheri* orthologs due to palindromic repeats at its end (Supplementary File 1). *Bl_op16* is flanked by a truncated and a complete copy of the DNA transposon Ginger2-1 and the non-autonomous DNA transposon Harbinger-N11 (data not shown).

**Figure 1:**
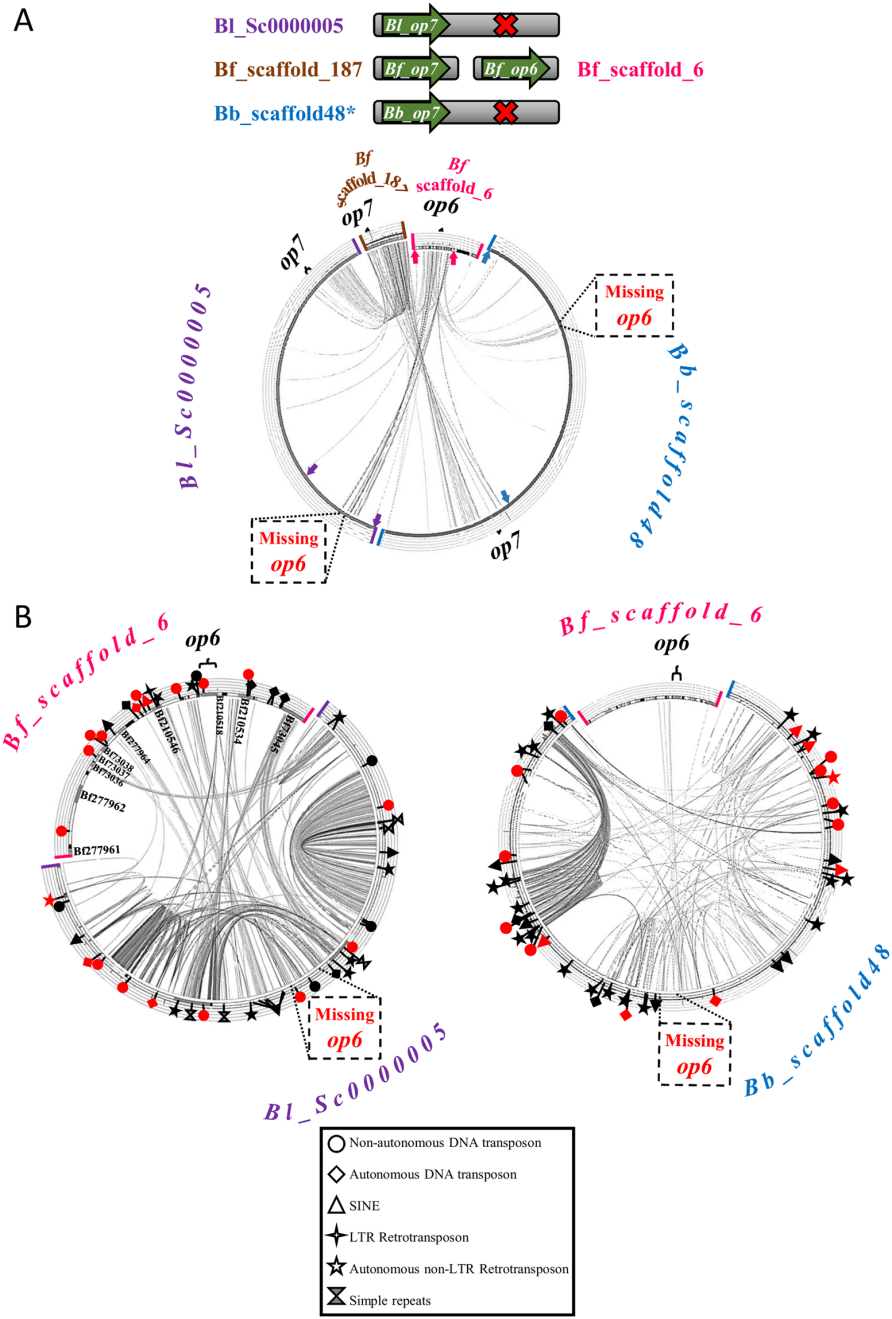
Comparison of genomic loci containing or lacking *op6* and *op7*. (**A**) Comparison of the *op6* containing Bf_scaffold_6 and the *op7* containing Bf_scaffold_187 with the Bl_Sc0000005 and the Bb_scaffold48 that apparently contain only *op7* and lack *op6*. (**B**) Comparison of more narrow regions of Bf_scaffold_6 (delimited by arrows in (**A**)), with Bl_Sc0000005 (left) and Bb_scaffold48 (right). A high degree of duplicated regions was observed for *B. lanceolatum*, with the most striking example that of Bf73045 (left). Duplicated regions were also observed for *B. belcheri* (right). Red and black (complete and partial copies based on the RepBase database) symbols mark the position of simple tandem repeats and various families of Transposable Elements (TEs) (see key legend for explanation and Supplementary Fig. 4A for TE names). For the sake of clarity, predicted *B. floridae* gene models are listed only in the internal part of the Bf_scaffold_6 in (**B**). Ribbons connecting syntenic scaffolds under comparison denote similarity at genomic level.

Comparison of the syntenic scaffolds related to *op6* is portrayed in Fig. 1. High similarity is observed among the genomic regions containing *op7* in *B. floridae*, *B. lanceolatum* and *B. belcheri* (Fig. 1A). Similarity is also observed between the genomic regions flanking *op6* in *B. floridae* and *B. lanceolatum *Sc0000005 and *B. belcheri* scaffold48, however, there are no traces of *op6* in the other two species. Some of the immediately flanking genes of *Bf_op6* have their orthologs in *B. lanceolatum* (only one seems to be eliminated, namely Bf210534), but are duplicated in the latter, with more striking example that of Bf73045 (Fig. 1B). Duplication of other genomic fragments in the region where *Bl_op6* was supposed to be is also evident. Numerous families of transposable elements and simple repeated sequences of varying size (2–65 bp) have been detected within and in the proximity of the duplicated genes and genomic fragments in Bl_Sc0000005 (see Supplementary Fig. 4A for names of TEs). A similar case of duplicated genomic fragments populated by transposable elements is also observed in *B. belcheri*. What is even more appealing is the number and type of transposable elements within *Bf_op6* and *Bf_op7* genes and in their vicinity (Supplementary Fig. 4B). No other conservation at genomic level is observed between *B. floridae* scaffolds 6 and 187, apart from the opsin genes and various transposable elements, as shown in Supplementary Fig. 4B.

**Figure 2:**
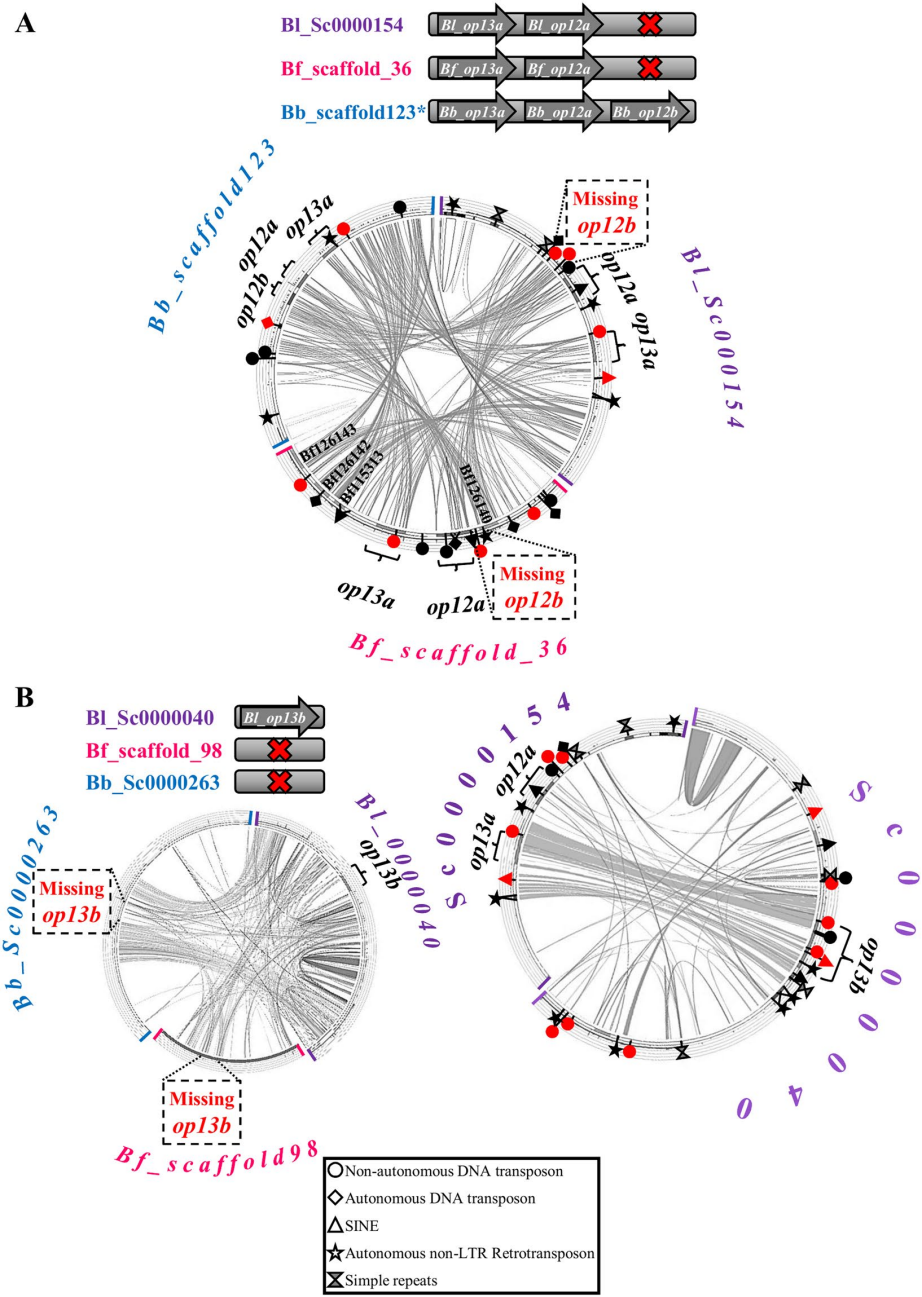
Comparison of genomic loci containing or lacking *op12a*, *op12b*, *op13a* and *op13b* opsins. (**A**) Comparison of Bl_Sc0000154 with Bf_scaffold_36 and Bb_scaffold_23 (**B**) Comparison of *B. lanceolatum* scaffold containing the *op13b* gene with the syntenic scaffolds from *B. floridae* (Bf_scaffold_98) and *B. belcheri* (Bb_Sc0000263). (**C**) Comparison of *B. lanceolatum* scaffolds bearing opsins *op13a* (Sc0000154) and *op13b* (Sc0000040). Red and black (complete and partial copies based on the RepBase database) symbols mark the position of simple tandem repeats and various families of transposable elements (TEs) (see key legend for explanation and Supplementary Fig. 4B and C for TE names). Predicted *B. floridae* gene models are listed in the internal part of the scaffolds.

Differences are observed among the three species in regard to *op12* and *op13* copies (Table 1, Fig. 2 and Supplementary Fig. 2). In general, these genes exhibit high sequence similarity and contain the same number of exons. Size of the exons is almost identical, with a strikingly smaller last exon in *Bb_op12b* (Supplementary File 1). Comparison of scaffolds bearing *op12a*, *op12b* and *op13a* from the three species (Fig. 2A) shows that there is high conservation in opsin genes as well as in their flanking regions. However, no significant similarity exists in the intergenic regions of *op12a* and *op13a*. Interestingly, opsin genes in *B. belcheri* are flanked by complete copies of DNA transposons (Supplementary Fig. 4C). The absence of *op13b* ortholog from *B. floridae* and *B. belcheri* is evident from the comparison of syntenic scaffolds (Fig. 2B). On the other hand, scaffolds containing the *B. lanceolatum op13a* and *op13b* paralogs (Fig. 2C, Supplementary Fig. 4C) show a high degree of similarity only in the genic regions and their immediate neighborhood which does not extend further in the region of *Bl_op12a*. The region of similarity is bordered by simple repeats as well as complete or partial copies of TEs.

**Figure 3:**
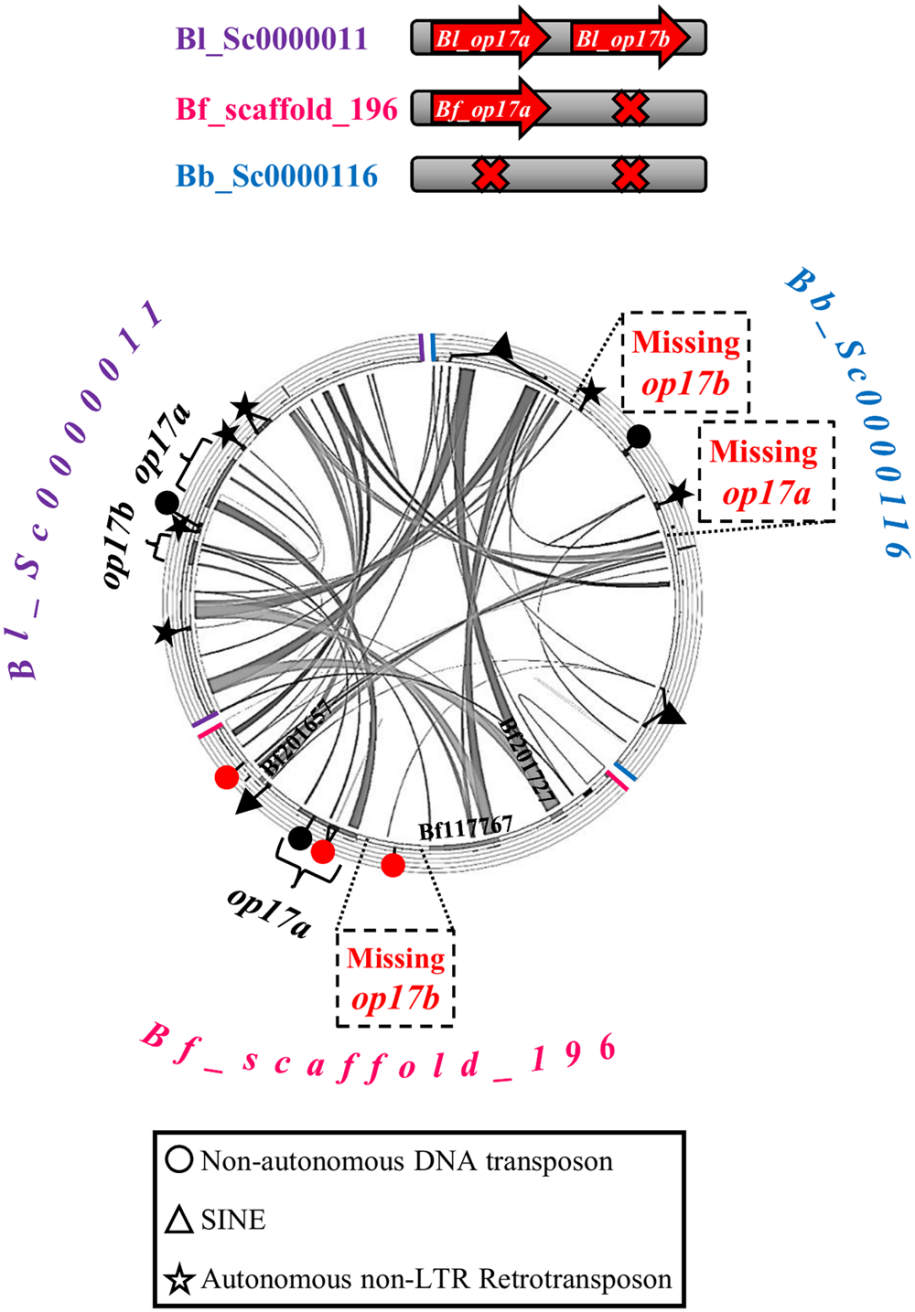
Comparison of genomic loci containing or lacking *op17a* and *op17b* opsins. Comparison of the *op17a* and *op17b* containing scaffold from *B. lanceolatum* with the *op17a*-containing *B. floridae* scaffold and the syntenic scaffold from *B. belcheri* that obviously lacks both *op17a* and *op17b*. A clear conservation of the genomic regions is observed. Red and black (complete and partial copies based on the RepBase database) symbols mark the position of various families of transposable elements (TE) (see key legend for explanation and Supplementary Fig. 4D for TE names). Predicted *B. floridae* gene models are listed in the internal part of the scaffolds.

Another example of putative gene duplication and loss event is that of *op17a* and *op17b* (Fig. 3). Using the neighboring genes of *Bf_op17a* we detected the syntenic scaffold in *B. belcheri*. Comparison of the three scaffolds shows conservation in the flanking regions but no traces of a *Bb_op17a* gene. Instead, in the region where *Bb_op17a* is expected to be, there are copies of retrotransposons^52^ (Supplementary Fig. 4D). *Bl_op17a* and *Bl_op17b* genes are as well flanked by autonomous and non-autonomous transposons.

**Figure 4:**
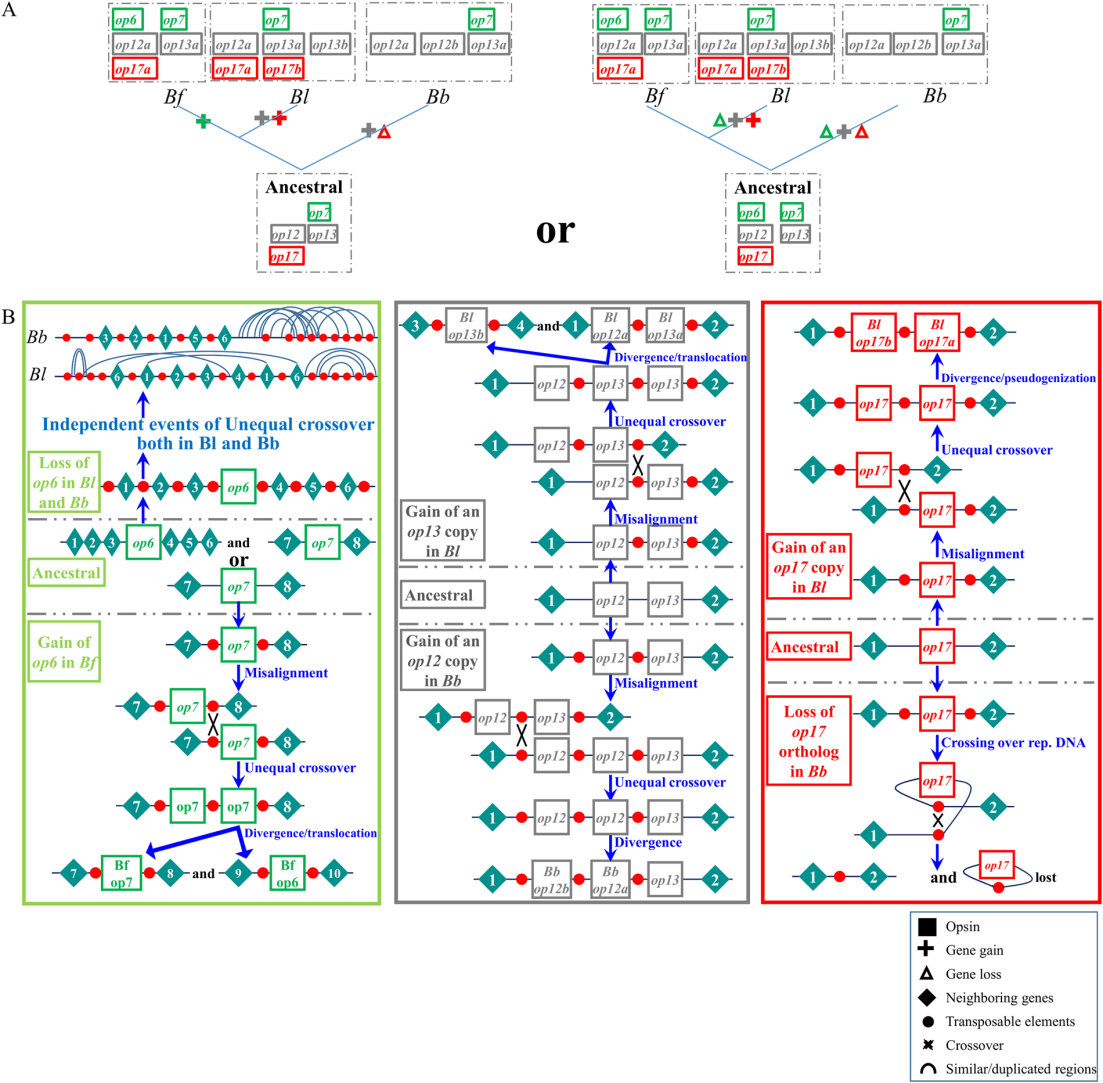
Reconstruction of the evolutionary history of opsin family in the *Branchiostoma* genus. (**A**) Schematic representation of gene gains and losses in the lineages of *B. lanceolatum, B. floridae and B. belcheri*. (**B**) *op6* was either lost independently in the lineages of *B. lanceolatum* and *B. belcheri* or duplicated in *B. floridae*, due to misalignment and unequal cross-over events, where Transposable Elements (TEs) were used as illegitimate recombination hotspots. Likewise, *Bb_op12b* and *Bl_op13b* were duplicated independently only in the genomes of *B. belcheri* and *B. lanceolatum*, respectively. Finally, *Bl_op17b* was duplicated in the genome of *B. lanceolatum* and later was rendered non-functional, whereas recombination over transposable elements eliminated *Bl_op17a* from the *B. belcheri* genome.

To summarize our previous findings, we could say that independent events of gene duplications and losses occurred during the evolution of *Branchiostoma* opsins (Fig. 4A). Taking into account the higher similarity between *B. lanceolatum* and *B. belcheri* regions, the almost identical structure of *Bf_op6* and *Bf_op7* and the presence of common transposable elements within and outside these two genes, we could conclude that *op6* is the result of a duplication event in *B. floridae*, after its split from *B. lanceolatum*. However, we cannot rule out the possibility that *op6* existed in the common ancestor of the *Branchiostoma* species and it was eliminated in the lineages of *B. lanceolatum* and *B. belcheri*. We could also conclude that *Bb_op12a* and *Bl_op13a* were independently duplicated in *B. belcheri* and *B. lanceolatum*. Finally, we assume that *op17a* was lost in *B. belcheri* and *op17b* is the result of a gene duplication only in *B. lanceolatum* (Fig. 4A). Figure 4B outlines what the ancestral state could have been for each of the duplicated/lost genes and the putative mechanisms through which gene gains and losses took place. Complete and partial copies of TEs identified in the vicinity of opsin genes probably served as illegitimate spots for recombination, leading to misalignment, unequal crossover and hence duplication of an opsin gene, as in the case of *op12* and *op13*, or caused crossing over of the same chromosome, leading to the deletion of *op17* in *B. belcheri*.

## Discussion

Cephalochordates are often used as a proxy to the ancestral chordates. This is in large part due to the presumed slow evolutionary rate of their genomes. In this study we used the *Branchiostoma* opsin gene family as an example of how TEs can shape cephalochordate genomes, by deleting or creating new genes, by altering the number and size of exons or influencing their expression patterns. We further reconstructed the evolutionary history of opsin family in the *Branchiostoma* genus, via comparison of primary sequence, structure and expression patterns of opsin genes from three cephalochordate species.

The species-specific duplicates *Bl_op13a* and *Bl_op13b* differ in their spatial (tissue-specific) but overlap in their temporal expression patterns and are already detected at an earlier stage than *B. floridae* (Pantzartzi *et al*.^40^ and Supplementary Fig. 3). The first one is indicative of subfunctionalization, where the two genes seem to have optimized for specific tasks in tissues with different type of photoreceptor cells (ciliary and rhabdomeric), while the latter implies that *Bl_op13a* underwent neofunctionalization, due to which expression is triggered at an earlier stage. The relatively large size of the Go group and the retention in the genome of the duplicated opsins (*Bb_op12b* and *Bl_op13b*) could be an indication of fine tuning between these opsins in order for specific photoreception-related tasks to be fulfilled. Similarly, retention of *Bf_op6* and *Bf_op7* in the genome of *B. floridae* could be attributed to subfunctionalization, since changes are noted in their temporal expression pattern (Supplementary Fig. 3B).

The role of transposable elements (TEs) in shaping the genome and promoting evolution has been the focus of many studies, and what was formerly characterized as “junk” or “selfish DNA” is gaining more and more value and functional importance^53^. TEs may act in the same or completely different way, depending on selection forces. This is nicely exemplified by the ParaHox loci in *Ciona*, amphioxus and vertebrates^54,55^. ParaHox cluster in *Ciona* has lost the tight organization present in chordates and this degeneration could be attributed to the invasion of TEs in the locus, specifically of MITEs^55^. On the other hand, even though the amphioxus ParaHox cluster was found to be a hotspot for TE insertion, selection constraints probably inhibit this disruptive elements from influencing the ParaHox locus^54^. Another example of how TEs may influence the gene structure is that of *PRHOXNB* gene, for which the gain of an intron was reported, in which the miniature inverted-repeat transposable element (MITE) LanceletTn-2 was detected^56^.

An increase in the number of opsin gene has been previously reported for various species, owing either to local gene duplications^57^ or whole genome duplications^58^. In some cases, the number or structure of opsin genes seems to be shaped under the influence of TEs^59–61^. The presence of an incomplete *Alu* element upstream the human middle wavelength sensitive (MW) opsin gene may imply that *Alu* elements have been involved in the initial gene duplication responsible for the MW and long-wavelength sensitive (LW) genes in the Old World primates and the high frequency of gene loss and gene duplication within the opsin gene array^60^. It is suggested that unequal crossover is the mechanism through which this duplication occurred^60^. In the swordtail fish, *Xiphophorus helleri*, one of the four LW copies was found to be the result of a retrotransposition event^59^. On the other hand, the loss of function of the *Takifugu rubripes* RH2-2 gene is reported to follow a transposon-induced deletion that truncated the N-terminal of the protein^61^.

**Figure 5:**
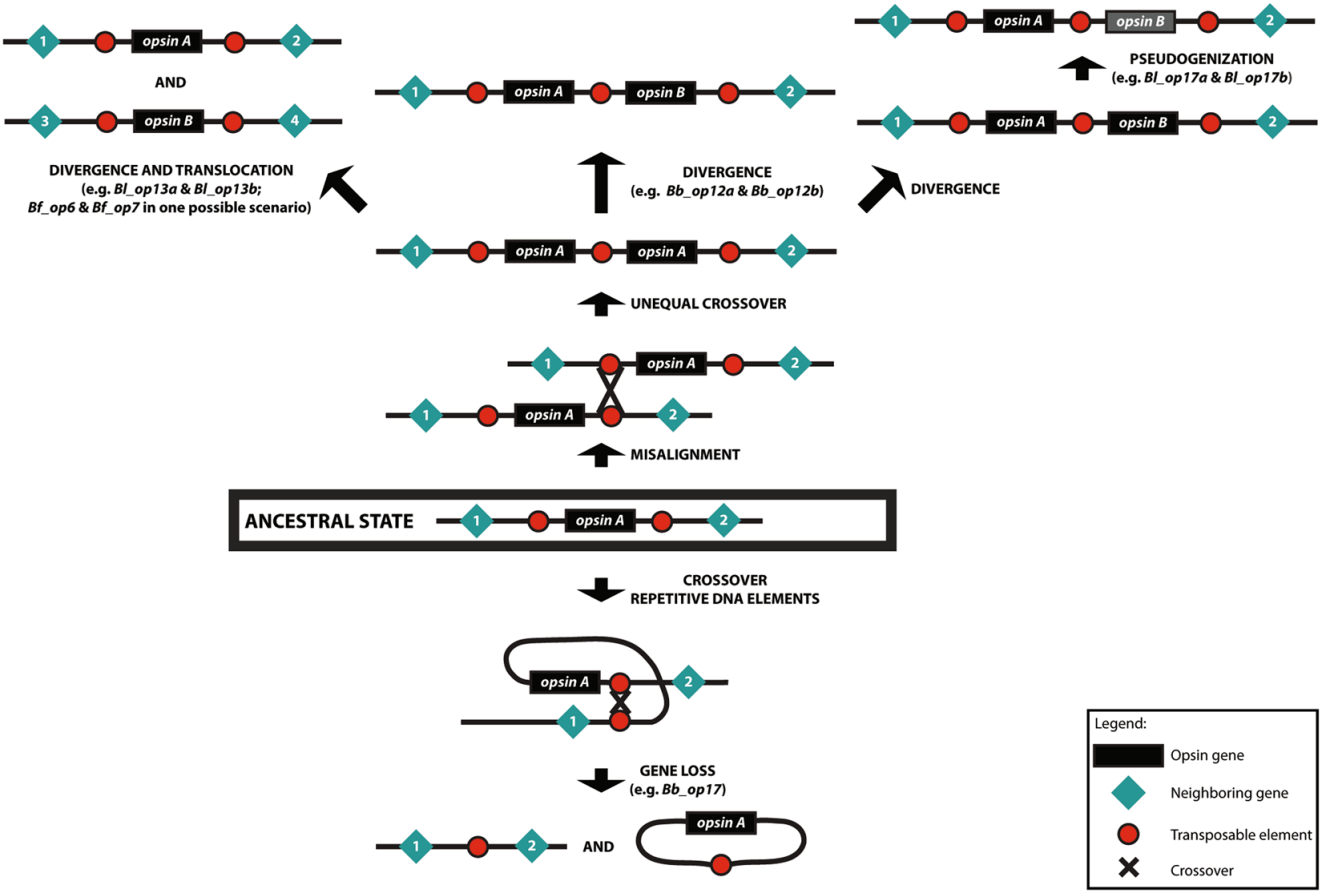
Suggested mechanisms for the evolution of a gene family, under the effect of Transposable Elements, as exemplified by the opsin family in the *Branchiostoma* genus.

We have provided information about how TEs might have led to gene duplications and losses in the *Branchiostoma* opsin family, or alterations in the number and size of exons. In fact, the *Branchiostoma* opsin family could serve as an example of how TEs can play an important role in the shaping of a gene family and of the genome per se, through gene gain and loss events due to unequal cross-over or moving of genes between different loci in the genome (Fig. 5). Moreover, TEs may also lead to neofunctionalization of duplicate genes, which typically occurs by the acquisition of new regulatory elements. Overrepresentation of transcription factor binding sites is evident for TEs residing in promoter regions of not only human genes^11^, but of amphioxus as well^62^. Retention of *Branchiostoma* gene copies in the genome and differences in their spatiotemporal expression pattern, together with the presence of different types of TEs, could also imply that TEs were not implicated only in the birth or death of opsin genes but in their control as well.

## Electronic supplementary material

Supplementary Information
