# Supplementary material for: The role of transposable elements in functional evolution of amphioxus genome: the case of opsin gene family
Source: Sci Rep. 2018 Feb 6;8:2506. doi: 10.1038/s41598-018-20683-9 (PMC5802833; doi:10.1038/s41598-018-20683-9)
Supplement: Supplementary file 1 — Supplementary Information [file 41598_2018_20683_MOESM1_ESM.pdf]

Article

The role of transposable elements in functional evolution of amphioxus genome: the case of opsin gene family

Chrysoula N. Pantzartzi[1], Jiri Pergner[2], Zbynek Kozmik[1,2,*]

[1]Laboratory of Eye Biology, Institute of Molecular Genetics of the ASCR, v.v.i., Division BIOCEV, Prumyslová595, 252 50 Vestec, Czech Republic

[2]Department of Transcriptional Regulation, Institute of Molecular Genetics of the ASCR, v.v.i., Videnska 1083, 14220,Prague 4, Czech Republic

* Author for Correspondence: Zbynek Kozmik, Institute of Molecular Genetics of the ASCR, v.v.i., Videnska 1083, 14220, Prague, Czech Republic, +420 241062110, kozmik@img.cas.cz

op1 + Amphiop5

op1							
B. lanceolatum	***B. floridae***				***B.belcheri***		**ScanProsite[5]**
Genomic scaffold in Assembly BraLan2[1]	JGI gene model (Putative allele or duplicate)[2]	NCBI gene model, Transcript and protein Accession numbers	Exon No and size[3]	Comments	Predicted gene model & scaffold in HapV2 assembly[4]	Comments	
Sc0000170<br>(505915..531566)<br><br><br>413aa<br>e1= 373<br>e2= 188<br>e3= 147<br>e4= 243<br>e5= 291	**84893+124039***<br>scaffold_136<br>(74630[a]<br>scaffold_52)	**74630***<br>scaffold_245<br>(345aa)<br><br>XM_002589165<br>XP_002589211	 <br><br><br>406aa<br>e1= 370<br>e2= 188<br>e3= 147<br>e4= 243<br>e5= 279	Scaffold retained in NCBI is not the correct one – exon 4 is missing from scaffold_245	048460*<br>Sc0000452<br><br><br>411aa<br>e1= 349<br>e2= 188<br>e3= 147<br>e4= 243<br>e5= 309		GPCR +<br>(57-310aa)<br>OPSIN +<br>(294-310aa)

B. floridae

>Bf_op1_1227bp
ATGTTAGGTATGCATAACGTCATGAACGCTACAGACTATGACAACAACAACGCAACGTTTGCTGCCTGGA
ACTTTCAAAGGAACGGCACAACTGAAGAAGAGGTTGAATTCTCAGGGTTCGACACCGTGGCTGTTGTCAT
CGCCGCCATCGGTATTGCAGGGTTCCTGAGCAATGGCGCGGTGGTTTTGCTGTTCCTGAAGTTCCGGCAG
CTGCGCACTCCCTTCAACATGCTGCTGCTGAACATGTCCGTGGCCGACCTGCTGGTGTCCGTGTGCGGCA
ACACGCTGTCCTTCGCCTCCGCCGTCCGTCACCGCTGGCTGTGGGGCAGGCCCGGCTGTGTATGGTACGG
GTTCGCCAATCACCTCTTTGGTCTGGTATCCCTGATCTCCCTGGCTGTCATCTCCTACGAGCGGTACCGG
ATGGTAGTCAAGCCGAAGGGCCCGGGCTCCTCTTACCTTACCTACAACAAAGTGGGCCTGGCCATTATCT
TCATCTACCTGTACTGTCTGCTGTGGACGACTCTACCAATCGTGGGGTGGAGCAGCTACCAACTCGAGGG
TCCTAAGATAAGCTGCTCCGTAGCATGGGAGGAGCACAGCCTGTCCAACACGTCTTACATCGTGGCTATC
TTCATCATGTGTCTGCTACTGCCTCTTCTCATCATCATATACTCCTACTGCCGCTTGTGGTACAAGGTGA
AGAAAGGTTCGCAGAACCTTCCACCAGCCATAAGAAAGTCGAGTCAGAAGGAACAGAAGATTGCCCGGAT
GGTAGTGGTGATGATCACGTGTTTCCTGGTGTGCTGGCTGCCGTACGGAGCCATGGCGCTGGTGGTGAGT
TTCGGCGGGGAGAGCCTGATCTCCCCCACCGCTGCAGTGGTTCCGTCTCTGCTCGCCAAGTCCAGCACCT
GCTACAACCCGCTGGTCTACTTCGCCATGAACAACCAGTTCCGCCGGTATTTCCAAGATCTTCTGTGCTG
CGGCCGCAGACTGTTTGACGCCTCTGCCTCTGTGAACACGTGTAACACCTCGGCCATGCCCAGACATTCC
CCAGTTTTCCAGAAACCCGACTCTGACCAGTACAATGGGATACAAAAGAGTAGAGAACCACAGATGAGAA
CAACGGGGCAAAACGCGCCATACCGACAGTGGATAGAGATGCAGACCATCGCCGTCGTCGTGAAGGCTGA
CGAAGTAAACAACAAATTTGGCGAGGTTAAAACGTAA

>Bf_op1_408aa
MLGMHNVMNATDYDNNNATFAAWNFQRNGTTEEEVEFSGFDTVAVVIAAIGIAGFLSNGAVVLLFLKFRQ
LRTPFNMLLLNMSVADLLVSVCGNTLSFASAVRHRWLWGRPGCVWYGFANHLFGLVSLISLAVISYERYR
MVVKPKGPGSSYLTYNKVGLAIIFIYLYCLLWTTLPIVGWSSYQLEGPKISCSVAWEEHSLSNTSYIVAI
FIMCLLLPLLIIIYSYCRLWYKVKKGSQNLPPAIRKSSQKEQKIARMVVVMITCFLVCWLPYGAMALVVS
FGGESLISPTAAVVPSLLAKSSTCYNPLVYFAMNNQFRRYFQDLLCCGRRLFDASASVNTCNTSAMPRHS
PVFQKPDSDQYNGIQKSREPQMRTTGQNAPYRQWIEMQTIAVVVKADEVNNKFGEVKT

B. belcheri

>Bb_op1_1236bp
ATGCTGGGCATTTATAATGACTACGGTAACAACAACACGACGTTTGCCGCCTGGAACTTTCAAAGGAACG
GCACTGAAGAAGTCGACTTCTCCGAGTACGAGGCAGTTGCCGGCGTGATAGCGGTGATCGGTCTCGTGGG
GTTCGTGAGTAACGGCGCGGTGGTGGTCCTGTTCCTGAAGTTCCGGCAGCTGCGCACGCCCTTCAACCTG
CTGCTGCTGAACATGTCCGTGGCCGACCTGCTGGTGTCCGTGTGCGGCAACACGCTGTCCTTCGCCTCCG

```
CCGTCCAACACCGCTGGCTGTGGGGCCGGCCCGGCTGTGTCTGGTACGGCTTCGCCAATCATCTCTTTGG
TCTGGTGTCGCTGATCTCCCTGGCGGTCATCTCCTACGAGCGGTACCGGATGGTGGTGAAACCGAAGGGT
CCGGGCTCCTCTTATCTCACCTACAACAAAGTGGGCCTGGCCATCCTCTTCATCTACCTGTACTGTCTGT
TGTGGACGGCTCTACCTATCGCGGGGTGGAGCAGCTACCAACTCGAGGGTCCTAAGATCGGGTGCTCCGT
GGCCTGGGAGGAACACAGCTGGTCCAGCACGTCTTACATCGTGGCCCTGTTCACCACGTGTCTGTTCGCG
CCTCTTCTCGTCATCATCTACTCCTACTACCGGCTGTGGTACAAGGTGAAGGAAGGTTCGCGGAACCTGC
CTGCGGCTATGAGAAAGTCGAGTCAGAAGGAGCAGAAAATCGCCATGATGGTAGTGGTGATGATCACGTG
TTTCCTGGTGTGCTGGCTGCCGTACGGCGCCATGGCCCTGGTGGTGAGTTTCGGCGGGGAGCGGCTGATC
TCGAACACCGCCGCCGTGGTGCCGGCTCTCATGGCCAAGTCCAGCACCTGCTACAACCCGGTCGTCTACT
TCGCCATGAACAGCCAGTTCCGCCGCTACTTCCAGGAGCTACTGTGCTGCGGTCGCAGACTGTTTGATGC
CTCTGCGTCGGCAGGTACGTGTAACACAGCCGTGCCAGGGAAAAATTCGCAAGGTTTCCGGAAACCCAAC
TCTGACCATGACAACGGACTGCCGAAGCAAACCGAAGGATCTGTGAGCGACCACGCTTGTAATGATGAAT
CAGAGATGGAGGGAGCCCGGCAGGATACGGCATCGCAACAGTGGATAGAGATGCAAGCTGTCGCTATCGT
CGTGAAGGCTGTTGAAGTCGACACCGATGCAGAAAATACGCCATAA
```

```
>Bb_op1_411aa
MLGIYNDYGNNNTTFAAWNFQRNGTEEVDFSEYEAVAGVIAVIGLVGFVSNGAVVVLFLKFRQLRTPFNL
LLLNMSVADLLVSVCGNTLSFASAVQHRWLWGRPGCVWYGFANHLFGLVSLISLAVISYERYRMVVKPKG
PGSSYLTYNKVGLAILFIYLYCLLWTALPIAGWSSYQLEGPKIGCSVAWEEHSWSSTSYIVALFTTCLFA
PLLVIIYSYYRLWYKVKEGSRNLPAAMRKSSQKEQKIAMMVVVMITCFLVCWLPYGAMALVVSFGGERLI
SNTAAVVPALMAKSSTCYNPVVYFAMNSQFRRYFQELLCCGRRLFDASASAGTCNTAVPGKNSQGFRKPN
SDHDNGLPKQTEGSVSDHACNDESEMEGARQDTASQQWIEMQAVAIVVKAVEVDTDAENTP
```

```
Bl_op1_413aa         1 ------------------------------------------MLGIYNVFNMTEYGNNNN
Bf_op1_408aa         1 ------------------------------------------MLGMHNVMNATDYDNN-N
Bb_op1_411aa         1 ------------------------------------------MLGIYNDYG----NN--N
Amphiop5_BAC76022    1 MSSVYDRNVSNSRWEGQVPTLATLRGNTDLIGQPRDWSTETVMLGIYNVVNATEYGN--N

                                                        TM I
                                                 #####################
Bl_op1_413aa        19 ATSAAWNFRRNGTA-EEEVEFSGFEPVAGVIAIIGIVGFLSNGAVVLLFLKFRQLRTPFN
Bf_op1_408aa        18 ATFAAWNFQRNGTT-EEEVEFSGFDTVAVVIAAIGIAGFLSNGAVVLLFLKFRQLRTPFN
Bb_op1_411aa        13 TTFAAWNFQRNGT---EEVDFSEYEAVAGVIAVIGLVGFVSNGAVVVLFLKFRQLRTPFN
Amphiop5_BAC76022   59 TTFAAWDFKRNGTGGEEEVEFFGYDAVAGVIAIIGVVGFVSNGAVVVLFLKFPQLRTPFN

                                 TM II                     TM III
                          ######################        ######################
Bl_op1_413aa        78 MLLLNMSVADLLVSVCGNTLSFASAVRHRWLWGRPGCVWYGFVNHLFGLVSLISLAVISF
Bf_op1_408aa        77 MLLLNMSVADLLVSVCGNTLSFASAVRHRWLWGRPGCVWYGFANHLFGLVSLISLAVISY
Bb_op1_411aa        70 LLLLNMSVADLLVSVCGNTLSFASAVQHRWLWGRPGCVWYGFANHLFGLVSLISLAVISY
Amphiop5_BAC76022  119LLLLNMAVADLLVSVCGNTLSFASAVRHRWLWGRPGCVWYGFANHLFGLVSLISLAVISF

                                                         TM IV
                                                 ######################
Bl_op1_413aa       138 ERYRMVVKPKGPGSSYLTYNKVGLAILFIYLYCLLWTTLPIVGWSSYELEGPEIGCSVAW
Bf_op1_408aa       137 ERYRMVVKPKGPGSSYLTYNKVGLAIIFIYLYCLLWTTLPIVGWSSYQLEGPKISCSVAW
Bb_op1_411aa       130 ERYRMVVKPKGPGSSYLTYNKVGLAILFIYLYCLLWTALPIAGWSSYQLEGPKIGCSVAW
Amphiop5_BAC76022  179 LRYRMVVKPKGPGSSYLTYTKVGLAILFIYLYCLLWTTLPIAGWSSYQLEGPKIGCSVAW

                                         TM V
                                 #####################
Bl_op1_413aa       198 EEHSLSNTSYIVVLFIMCLVAPLLIIIYSYYRLWYKVKKGSRNLPAAIRKSSQKEQKIAR
Bf_op1_408aa       197 EEHSLSNTSYIVAIFIMCLLLPLLIIIYSYCRLWYKVKKGSQNLPPAIRKSSQKEQKIAR
Bb_op1_411aa       190 EEHSWSSTSYIVALFTTCLFAPLLVIIYSYYRLWYKVKEGSRNLPAAMRKSSQKEQKIAM
Amphiop5_BAC76022  239EEHSWSNTSYIVVLFITCLFAPLLIIVYSYYRLWHKVKQGSRNLPAAMRKSSQKEQKIAM

                                 TM VI                      TM VII
                        ########################        #########*############
Bl_op1_413aa       258 MVVVMITCFMVCWLPYGAMALVVSFGGERLISHTAAVVPSLLAKSSTCYNPVVYFAMNSQ
Bf_op1_408aa       257 MVVVMITCFLVCWLPYGAMALVVSFGGESLISPTAAVVPSLLAKSSTCYNPLVYFAMNNQ
Bb_op1_411aa       250 MVVVMITCFLVCWLPYGAMALVVSFGGERLISNTAAVVPALMAKSSTCYNPVVYFAMNSQ
```

```
Amphiop5_BAC76022  299 MVIVMITCFMVCWLPYGAMALVVTFGGERLISHTAAVVPSLLAKSSTCYNPVVYFAMNSQ

Bl_op1_413aa       318 FRRYFQDLLCCGRRLFDTSASQHTCNTIALAKPSQDLRKPDSDR--NNGLPTRSYIAS--
Bf_op1_408aa       317 FRRYFQDLLCCGRRLFDASASVNTCNTSAMPRHSPVFQKPDSDQ--YNGIQKS-------
Bb_op1_411aa       310 FRRYFQELLCCGRRLFDASASAGTCNTAVPGKNSQGFRKPNSD--HDNGLPKQTEGSVSD
Amphiop5_BAC76022  359 FRRYFQDLLCCGRRLFDVSQSVVTGNTAMPRNNSQGFRKDDSDQKQDNGLPKQSEGPMCD

Bl_op1_413aa       374 -----GEQQTGESRQNTASQQWIELQTIAVAIKTDEVDNGAANTS-
Bf_op1_408aa       368 -----REPQMRTTGQNAPYRQWIEMQTIAVVVKADEVNNKFGEVKT
Bb_op1_411aa       368 HACNDESEMEGAR-QDTASQQWIEMQAVAIVVKAVEVDTDAENTP-
Amphiop5_BAC76022  419 HSSNES-QMEGSRHNTAASQQWIEMQTIAVVVKAVEVDTSAANEP-
```

op2

op2							
B. lanceolatum	***B. floridae***				***B.belcheri***		**ScanProsite**[5]
Genomic scaffold in Assembly BraLan2[1]	JGI gene model (Putative allele or duplicate)[2]	NCBI gene model, Transcript and protein Accession numbers	Exon No and size[3]	Comments	Predicted gene model & scaffold in HapV2 assembly[4]	Comments	
Sc0000009<br>(4027267..4043520)<br><br>420aa<br>e1= 45<br>e2= 247<br>e3= 329<br>e4= 345 Repeats<br>e5= 297!!	**205982**<br>scaffold_28<br>(70446[m]206170[d] scaffold_28)	**206170**<br>scaffold_22<br>(272aa)<br><br>XM_002611044<br>XP_002611090	 <br><br><br>405aa<br>e1= 45<br>e2= 247<br>e3= 329<br>e4= 312<br>e5= 285		254850*<br>Sc0000035<br><br><br>389aa<br>e1= 45<br>e2= 247<br>e3= 329<br>e4= 270<br>e5= 279		GPCR +<br>(31-305aa)<br>OPSIN -<br>**K296** is at position 295

B. floridae

```
>Bf_op2_1218bp
ATGGATCCTACCGATGAAACCCTAATGTCAGACGGCGGGCTCGAGCCTGTGGCGGCGATCCTGGCCCTTA
TCGGCGTGCTGGGCATAGTGAACAACTCCACCACCCTGTACCTCGTGGGCCGGTACAAACAGCTCCGGAC
ACCGTTCAACATCCTGATGGTTAACCTGTCGGTCAGTGACCTCTTGATGTGTGTCCTGGCCACACCCTTC
AGCTTCGTGTCCAGTCTGCACGGCCGCTGGATGTTCGGCCACTCTGGCTGTGAGTGGTATGGCTTCATCT
GCAACTTTCTGGGCATTGTGTCCTTGATCACCTTGACGGTCATCTCCTACGAACGGTACCTGCTGATGAA
GCGGCTCCCAAACGAACGTATTCTGTCGTACCGCGCGGTGGCGCTCGCCGTGGTCTTCATCTGGTGCTAC
AGCCTGCTGTGGACGGCTCCACCGCTGGTGGGGTGGAGCAGCTACGGACCGGAGGGCTACGGCATCTCCT
GCTCGGTCAACTGGGAGTCACGCACCGCCAACGACACATCCTACATCGTGGCCTACTTCGTGGGTTGCCT
GGTCTTTCCCGTCGCCATCATAGTTATCTCGTACACCCGTCTCATCCTTTACATGCGTCAGCAACTACCG
GACGCAACGCAGGGTACACAGAAGCAAGCACAAGGTGCACAACAGCAGCCACAAGGTGCGCAACAGCAGG
CCCCATCTGCACCGATGCAGATGCTGGTGCGGCGAGAGAAGAGAGTGACCAAGATGGTGGTGGTGATGAT
CATGGGGTTCACGATCTGCTGGACCCCTTACACCATCGTGGCGCTGATCGTCACCTGCGGGGGAGAGGGC
ATCATCACCCCGGCGGCTGCCACGGTGCCCGCGCTCTTCGCCAAGTCCAGTGTGGTCTACAACGCCGCCA
TCTATGTGGCCATGAACAATCAGTTCCGGAAGTGTTTCCTAAGATCCTTGAACTGCCGGTCCCAACCACG
TGACCCTTCGTCCCAGCAGTACACCTTGAAGACCAACCAGGTCGGCATGTCGACCAGCGGCTCACAAGCG
GCTCGCACTGCGGACAGGATCAAGACCGTGCACGTCGCCACAGCCAATCCACAAGACCATCGCTCAAGCT
CGGGACAAGCTGTAGAGGATAACGGGGGGTTTCGGAAATCCCTAACTCACAGCCTTCCGCTTAATAGCAT
TTCCACCCTATTAGAGGCAGAGAAATAA
```

```
>Bf_op2_405aa
MDPTDETLMSDGGLEPVAAILALIGVLGIVNNSTTLYLVGRYKQLRTPFNILMVNLSVSDLLMCVLATPF
SFVSSLHGRWMFGHSGCEWYGFICNFLGIVSLITLTVISYERYLLMKRLPNERILSYRAVALAVVFIWCY
SLLWTAPPLVGWSSYGPEGYGISCSVNWESRTANDTSYIVAYFVGCLVFPVAIIVISYTRLILYMRQQLP
DATQGTQKQAQGAQQQPQGAQQQAPSAPMQMLVRREKRVTKMVVVMIMGFTICWTPYTIVALIVTCGGEG
IITPAAATVPALFAKSSVVYNAAIYVAMNNQFRKCFLRSLNCRSQPRDPSSQQYTLKTNQVGMSTSGSQA
ARTADRIKTVHVATANPQDHRSSSGQAVEDNGGFRKSLTHSLPLNSISTLLEAEK
```

B. belcheri

```
>Bb_op2_1170bp
ATGACTTCAGCAGACGTGGCACTCATCTCAGACGGCGATCTACAGGCCGTGGCCTCGGTCCTGGCAATCA
TTGCCGTGTTGGGGATTGTGAATAACTCCACCACCCTGTACCTCGTTGGGCGGTACAGCCAGCTGCGAAC
ACCCTTCAACATGCTGGTGGTGAACCTCACGGTCAGCGACCTGCTGGTGTGCGTCCTCGGCACGCCCTTC
AGCTTTGTCTCTAGTCTGAAGGGCCGGTGGATGTTCGGGCGTGCCGGCTGTGTGTGGTACGGCTTCATCA
```

ACAGTCTGCTGGGTATCGTGTCCCTGACCACCTTGACAGTCATCTCCTACGAGCGCCACCAGATGATGAA
GCGGCCCCCAAACGCACCCAAGCTGTCCTACCGCTGGGTGGCACTTTCCGTGCTGTTCGTCTGGGTCTAC
AGCCTGCTCTGGACGGTCCCGCCGTTGATGGGGTGGAGCAGCTACGGTCCCGAGTCTCACGGCGTGAGCT
GCTCGGTCAACTGGGTGTCGCGCACCGCCAACGATACCTCCTACATCGTGGCGTTCTTCGTGGGCTGCCT
GGCTGTTCCTGTCGCCGTCATAGTTGTCTCCTACACCCGTCTGGTCCTACATGTGCGGCGGGCACAAGAG
CAGCTGCCAGATGCACCTCCACAGCTGGGAGGTGCCGCCCAGCAGTCCAGCATCACCCGTCGTGAGAAGA
GAGTGACGTGGATGGTGGTGGTGATGGTGGCGTGTTTCGTGGTGTGCTGGCTGCCGTACGGCGTCATGGC
GCTGGTCGTGACGTTCGGCGGAGAGGAGATGGTGACCCCGGAGGCCGCCATGGTGCCCTCGCTGTTCGCC
AAGTCCAGCGTGGCGTACAACGCCGGCATCTACGTCGCCATGAACAGCCAGTTCCGGAGGTGTTTCTTGA
GCTGCTTCAAGTGCCGGTCCCTGCAGCCGGACCGCACGTCCCAGCAGTACGCCTGCAAGAACAGCCAAGT
CGGTGTCTCGACCTGCAGTACACAAGTGGATCGCAACAACAGCTCGGGCGCTGCTGCCACGTGTAATGCG
CAAGCTAATGGTTCAAACTCTGCTGGGCGCAGTGCAGATAGTAATCCGGGGTTTAAGAAGCCCCACCATT
ACGGTTTTCCACTTACTAAAATCTGTCCCATATCAGAGGGTGAAGAGTAA

>Bb_op2_389aa
MTSADVALISDGDLQAVASVLAIIAVLGIVNNSTTLYLVGRYSQLRTPFNMLVVNLTVSDLLVCVLGTPF
SFVSSLKGRWMFGRAGCVWYGFINSLLGIVSLTTLTVISYERHQMMKRPPNAPKLSYRWVALSVLFVWVY
SLLWTVPPLMGWSSYGPESHGVSCSVNWVSRTANDTSYIVAFFVGCLAVPVAVIVVSYTRLVLHVRRAQE
QLPDAPPQLGGAAQQSSITRREKRVTWMVVVMVACFVVCWLPYGVMALVVTFGGEEMVTPEAAMVPSLFA
KSSVAYNAGIYVAMNSQFRRCFLSCFKCRSLQPDRTSQQYACKNSQVGVSTCSTQVDRNNSSGAAATCNA
QANGSNSAGRSADSNPGFKKPHHYGFPLTKICPISEGEE

```
                         TM I                          TM
                  #########################          ##########
Bl_op2_420aa    1 MAPTDEAPMSDGELKAVAAVLGIIGVLGIVNNTTTLYLVGRYSQLRTPFNMLVVNLTVSD
Bf_op2_405aa    1 MDPTDETLMSDGGLEPVAAILALIGVLGIVNNSTTLYLVGRYKQLRTPFNILMVNLSVSD
Bb_op2_389aa    1 MTSADVALISDGDLQAVASVLAIIAVLGIVNNSTTLYLVGRYSQLRTPFNMLVVNLTVSD

                    II                                TM III
                  ############                ######################
Bl_op2_420aa   61 LLVCVLGTPFSFVSSLNGRWAFGHAGCVWYGFINSLLGIVSLITLTVISYERHQLMKRPP
Bf_op2_405aa   61 LLMCVLATPFSFVSSLHGRWMFGHSGCEWYGFICNFLGIVSLITLTVISYERYLLMKRLP
Bb_op2_389aa   61 LLVCVLGTPFSFVSSLKGRWMFGRAGCVWYGFINSLLGIVSLTTLTVISYERHQMMKRPP

                                   TM IV
                           #####################                    ###
Bl_op2_420aa  121NAPKLSYRWVALSVVFVWGYSLLWTVPPLMGWSSYGPEVHGVSCSVNWASRTANDTSYIV
Bf_op2_405aa  121 NERILSYRAVALAVVFIWCYSLLWTAPPLVGWSSYGPEGYGISCSVNWESRTANDTSYIV
Bb_op2_389aa  121 NAPKLSYRWVALSVLFVWVYSLLWTVPPLMGWSSYGPESHGVSCSVNWVSRTANDTSYIV

                       TM V
                  ####################
Bl_op2_420aa  181AYFVGCLAVPVATIVMSYTRLILHVRQAQQPLSDAIQPLPGAQQLPGAQQLPGAQPLPGA
Bf_op2_405aa  181 AYFVGCLVFPVAIIVISYTRLILYMRQQL---PDATQGT--QKQAQGAQQQ----PQGAQ
Bb_op2_389aa  181 AFFVGCLAVPVAVIVVSYTRLVLHVRRAQEQLPDAPPQLGGA------------------

                                   TM VI
                                         ######################             ##
Bl_op2_420aa  241QQLPAAPQQVPCTTRREKRVTKMVVVMVMCFLVCWLPYGIVALIVTFGGEGIITPEASMV
Bf_op2_405aa  232 QQAPS--APMQMLVRREKRVTKMVVVMIMGFTICWTPYTIVALIVTCGGEGIITPAAATV
Bb_op2_389aa  223 -------AQQSSITRREKRVTWMVVVMVACFVVCWLPYGVMALVVTFGGEEMVTPEAAMV

                        TM VII
                  #####*##############
Bl_op2_420aa  301PGLFAKSSVAYNAAIYVAMNSQFRKCFLSSFKCGSQHQQDTTSQLYASYAGKSSQVGAST
Bf_op2_405aa  290 PALFAKSSVVYNAAIYVAMNNQFRKCFLRSLNCRSQPRDPSS----QQYTLKTNQVGMST
Bb_op2_389aa  276 PSLFAKSSVAYNAGIYVAMNSQFRRCFLSCFKCRSLQPDRTS----QQYACKNSQVGVST

Bl_op2_420aa  361 CSFQVDRTVDRNNILNVAASNAQGHCSSSPRQADSIRGFRKSLPYSFPLTKICPISEVEE
Bf_op2_405aa  346 SGSQAARTADRIKTVHVATANPQDHRSSSGQAVEDNGGFRKSLTHSLPLNSISTLLEAEK
```

```
Bb_op2_389aa   332 CSTQVDRNNSSGAAATCNA--QANGSNSAGRSADSNPGFKKPHHYGFPLTKICPISEGEE
```

op3 + Amphiop4

op3							
B. lanceolatum	***B. floridae***				***B.belcheri***		**ScanProsite**[5]
Genomic scaffold in Assembly BraLan2[1]	JGI gene model (Putative allele or duplicate)[2]	NCBI gene model, Transcript and protein Accession numbers	Exon No and size[3]	Comments	Predicted gene model & scaffold in HapV2 assembly[4]	Comments	
Sc0000170<br>(545386..553817)<br><br>401aa<br>e1= 190<br>e2= 219<br>e3= 185<br>e4= 138<br>e5= 240<br>e6= 234	**84894***<br>scaffold_136<br>(74631[a]<br>scaffold_52)	**74631***<br>scaffold_245<br>(391aa)<br><br>XM_002589164<br>XP_002589210	401aa<br>e1= 193<br>e2= 219<br>e3= 185<br>e4= 138<br>e5= 240<br>e6= 231	Scaffold retained in NCBI is not the correct one	048450<br>Sc0000452<br><br>405aa<br>e1= 190<br>e2= 219<br>e3= 185<br>e4= 138<br>e5= 240<br>e6= 246		GPCR +<br>(71-319aa)<br>OPSIN +<br>(303-319aa)

B. floridae

```
>Bf_op3_1206bp
ATGGCGCTCTACAACAACACTTCGAGTCCGTTTCAGGACATTCTATGGGACGCCCCGTACAGCCAGGGTC
ACATCTGGGACAATTCTTCGACGTCCAACGCGACCGAAGACGTCATGGGTCAAGGAAAGGTGGAACTTCA
AGATTTCAGTGACTCCGGATATACAACAATCGCTACGTGTCTGGCACTTATAGGATTTGTGGGTTTCACG
AACAACTTCGTGGTGATTCTGCTGATTGGCTGTCATCGGCAGCTGCGCACTCCCTTCAACCTGCTGCTGC
TGAACATGTCCGTGGCAGACCTGCTGGTGTCCGTGTGCGGCAACACGCTGTCCTTCGCCTCCGCCGTCCG
ACACCGCTGGCTGTGGGGCAGGCCCGGCTGTGTCTGGTACGGGTTCGCCAACAGCTTGTTCGGTATTGTA
TCACTGGTGACCCTTAGTGCCCTGGCGTTCGAGCGGTATTGTGTAGTGGTGCGGAGTTCAGACATGTTGA
CCTACAAGTCTTCTCTAGGCGTCATCACATTTATCTGGCTCTACTCTCTCCTGTGGACGAGTTTGCCACT
GTTGGGCTGGAGCAGCTACCAGTTCGAAGGCCATAATGTCGGTTGTTCTGTGAACTGGGTGCAGCATAAC
CCGGACAATGTGTCCTACATCGTGACCCTGATGGTGACATGTTTCTTCGTACCGATGGTGGTCGTGTGTT
GGTCGTACGCCTGGATTTGGCGCACAGTACGGATGAGTTCGGAGGCAAAGCCTGAATATGGGAACTCCCA
GAACGCTGGTCGTCTGGTCACTACGATGGTCGTGGTCATGATCATTTGTTTCCTGGTCTGCTGGACGCCG
TACGCCGTCATGGCACTCATCGTCACATTCGGCGCGGATCACCTGGTGACACCGACGGCTTCCGTCATCC
CCTCGCTGGTGGCCAAGTCCAGCACGGCCTACAACCCCATCATCTACGTGCTGATGAACAACCAGTTTCG
AGAGTTTCTGCTGGCTCGTCTGCAGAGAGTCTGTTGCCGTCAGCAGGCCGTGCCGCGGGTCACACCGCTG
GACTACAACGTGCACGTCCGGCTTGGTGGAGAGGGTCCCAGCCAAGCACAGCAGTTTCTTCCGGCCGGGG
AAAATGTCGAAAACTTTGAAATGTTGAAATGTGTGCAGGAGAACTGCAAACTAAAAGCAGACTCACTCTC
CACTATATCAGAGTAG
```

```
>Bf_op3_401aa
MALYNNTSSPFQDILWDAPYSQGHIWDNSSTSNATEDVMGQGKVELQDFSDSGYTTIATCLALIGFVGFT
NNFVVILLIGCHRQLRTPFNLLLLNMSVADLLVSVCGNTLSFASAVRHRWLWGRPGCVWYGFANSLFGIV
SLVTLSALAFERYCVVVRSSDMLTYKSSLGVITFIWLYSLLWTSLPLLGWSSYQFEGHNVGCSVNWVQHN
PDNVSYIVTLMVTCFFVPMVVVCWSYAWIWRTVRMSSEAKPEYGNSQNAGRLVTTMVVVMIICFLVCWTP
YAVMALIVTFGADHLVTPTASVIPSLVAKSSTAYNPIIYVLMNNQFREFLLARLQRVCCRQQAVPRVTPL
DYNVHVRLGGEGPSQAQQFLPAGENVENFEMLKCVQENCKLKADSLSTISE
```

B. belcheri

```
>Bb_op3_1218bp
```

```
ATGCCGCTCTACAACGCCTCAGCTCCGGCCCATGACCTGCCCTGGGATACGCCGTACAGCCAGGATTACG
TCTGGAACGGTTCTTCTCCTTCAAACTCCAGCGAAGACGTAATAGAAGGAGGAAAGGAGGAACTTCAAGA
TTTCAGTGACGCCGGATACACGGCCATCGCTACGGGCCTGGCACTCATAGGAATTGTGGGCTTCGTGAAC
AACTTGGCCGTCATTCTGCTGATCGGCTGTCATCGGCAGCTGCGCACGCCCTTCAACCTGCTGCTGCTGA
ACATGTCCGTGGCCGACCTGCTGGTGTCCGTGTGCGGCAACACGCTGTCCTTCGCCTCCGCCGTCCAACA
CCGCTGGCTGTGGGGCCGGCCCGGCTGTGTCTGGTACGGGTTCGCCAACAGCTTGTTCGGTATTGTCTCA
CTGGTGACCCTGAGCGCCCTGGCGTTCGAGCGGTATTGTGTGGTGGTGCGGAGTTCGGACATGCTGACTT
ACAAGTCTTCTCTTGGCGTCATCACCTTTATCTGGCTGTACTCTCTCCTGTGGACAAGCTTACCACTGAT
GGGCTGGAGCAGCTACCAGTTCGAGGGTCACAGTGTCGGTTGCTCAGTGAACTGGGTGAAGAATAACCCC
GGTAACGTGTCCTACACCGTCACCCTGATGGTGACGTGTTTCTTCCTGCCGATGGCGGTTGTCTGCTGGT
CGTACGCCTGCATCTGGCGCACAGTCAGAATGAGCGCTGAGATGAAGTCAGTCTCCGGGAACCCGCAGAA
CTCTGGCCGTCTGGTGACGACGATGGTGGTGGTGATGATCGCGTGTTTCCTGGTGTGCTGGACCCCGTAC
ACCGTCATGGCGCTCATCGTCACCTTCGGCGCGGATCACCTGGTCACCCCGACCGCCTCCGTCATCCCGT
CTCTGGTGGCCAAGTCCAGCACGGCCTACAACCCTGTCATCTACGTGCTGATGAACAACCAGTTCCGTGA
GTTCCTGCTGGCCCGTCTCCGTACGTTCTGCTGCCGTCATCCGGGAGTGCCTCCCAGAGTTCCGCGCGTC
ACACCGCTGGACGACAACTGGCATGCGCATGCGCGGCTTGGCGGAGAAGGGCCCAGCCATGCGCAACAGT
TTATCCCGTCCGAGGACAAGGCAGAAAACATTGAAATGTTGCCGAAAGTACCGGAGAATCAGATGAAAGC
TGACTCCCTATCCACTATATCAGAATAA
```

```
>Bb_op3_405aa
MPLYNASAPAHDLPWDTPYSQDYVWNGSSPSNSSEDVIEGGKEELQDFSDAGYTAIATGLALIGIVGFVN
NLAVILLIGCHRQLRTPFNLLLLNMSVADLLVSVCGNTLSFASAVQHRWLWGRPGCVWYGFANSLFGIVS
LVTLSALAFERYCVVVRSSDMLTYKSSLGVITFIWLYSLLWTSLPLMGWSSYQFEGHSVGCSVNWVKNNP
GNVSYTVTLMVTCFFLPMAVVCWSYACIWRTVRMSAEMKSVSGNPQNSGRLVTTMVVVMIACFLVCWTPY
TVMALIVTFGADHLVTPTASVIPSLVAKSSTAYNPVIYVLMNNQFREFLLARLRTFCCRHPGVPPRVPRV
TPLDDNWHAHARLGGEGPSHAQQFIPSEDKAENIEMLPKVPENQMKADSLSTISE
```

```
                                                                                     ###
Bl_op3_401aa          1 MPLYNT-SRPTQDLPWDVPYGQDPVWNSSSTSNASQD-VIGQGKGELEEFSDSAYTAIAT
Bf_op3_401aa          1 MALYNNTSSPFQDILWDAPYSQGHIWDNSSTSNATED-VMGQGKVELQDFSDSGYTTIAT
Bb_op3_405aa          1 MPLYNA-SAPAHDLPWDTPYSQDYVWNGSSPSNSSED-VIEGGKEELQDFSDAGYTAIAT
Amphiop4_BAC76021     1 MPLYNTSSGPTQGLPWDTPYSQDPIWNDSSPSNSSEDAVVDQGRGELQDFSDAGYTAIAT

                              TM I                                 TM II
                        ###################                ######################
Bl_op3_401aa         59 GLALIGFVGFMNNFAVILLIGGHRQLRTPFNLLLLNMSVADLLVSVCGNTLSFASAVRHR
Bf_op3_401aa         60 CLALIGFVGFTNNFVVILLIGCHRQLRTPFNLLLLNMSVADLLVSVCGNTLSFASAVRHR
Bb_op3_405aa         59 GLALIGIVGFVNNLAVILLIGCHRQLRTPFNLLLLNMSVADLLVSVCGNTLSFASAVQHR
Amphiop4_BAC76021    61 GLALIGLVGSMNNFVVILLIGCHRQLRTPFNLLLLNVSVADLLVSVCGNTLSFASAVQHR

                                  TM III                                      TM
                                  ######################                ##########
Bl_op3_401aa        119 WLWGRPGCVWYGFANSLFGIVSLVTLSALAFERYCVVVRSSDMLTYKSSLGVITFIWMYS
Bf_op3_401aa        120 WLWGRPGCVWYGFANSLFGIVSLVTLSALAFERYCVVVRSSDMLTYKSSLGVITFIWLYS
Bb_op3_405aa        119 WLWGRPGCVWYGFANSLFGIVSLVTLSALAFERYCVVVRSSDMLTYKSSLGVITFIWLYS
Amphiop4_BAC76021   121WLWGRPGCVWYGFANSLFGIVSLVTLSALAFERYCVVVRSSEMLTYKSSLGMIAFIWMYS

                         IV                                         TM V
                        ############                           ######################
Bl_op3_401aa        179 LLWTSLPLLGWSSYQFEGHHIGCSVNWVEHNLGNVSYIITLMVTCFFVPMVVVCWSYAGI
Bf_op3_401aa        180 LLWTSLPLLGWSSYQFEGHNVGCSVNWVQHNPDNVSYIVTLMVTCFFVPMVVVCWSYAWI
Bb_op3_405aa        179 LLWTSLPLMGWSSYQFEGHSVGCSVNWVKNNPGNVSYTVTLMVTCFFLPMAVVCWSYACI
Amphiop4_BAC76021   181LLWTSLPLLGWSSYQFEGHSVGCSVNWVKHNVNNVSYIITLMVTCFFVPMVVVCWSYACI

                                                             TM VI
                                                   ######################
Bl_op3_401aa        239 WRTVRMSAEMKSEFGNPKNTGRLVTTMVVVMIVCFLVCWTPYAVMALIVTFGADHLVTPT
Bf_op3_401aa        240 WRTVRMSSEAKPEYGNSQNAGRLVTTMVVVMIICFLVCWTPYAVMALIVTFGADHLVTPT
Bb_op3_405aa        239 WRTVRMSAEMKSVSGNPQNSGRLVTTMVVVMIACFLVCWTPYTVMALIVTFGADHLVTPT
Amphiop4_BAC76021   241 WRTVRMSAEMKSEFGNPQNTGRLVTTMVVVMIVCFLVCWTPYTVMALIVTFGADHLVTPT

                                 TM VII
```

```
                          ########*#############
Bl_op3_401aa       299 ASVIPSLVAKSSTAYNPIIYVLMNNQFREFLLARLERLCCRQP----RAPRVTPIGIDDN
Bf_op3_401aa       300 ASVIPSLVAKSSTAYNPIIYVLMNNQFREFLLARLQRVCCRQQA----VPRVTPLDY--N
Bb_op3_405aa       299 ASVIPSLVAKSSTAYNPVIYVLMNNQFREFLLARLRTFCCRHPGVPPRVPRVTPLDDNWH
Amphiop4_BAC76021  301 ASVIPSLVAKSSTAYNPIIYVLMNNQFREFLLARLRTFCCRQP----RMLRVTPMDDN--

Bl_op3_401aa       355 TQVRLGGEGPSQAQPFLPSEENGDNVEMLTKAQEN-QLKGDGLSIISE
Bf_op3_401aa       354 VHVRLGGEGPSQAQQFLPAGENVENFEMLKCVQENCKLKADSLSTISE
Bb_op3_405aa       359 AHARLGGEGPSHAQQFIPSEDKAENIEMLPKVPEN-QMKADSLSTISE
Amphiop4_BAC76021  355 AHARLVGEGPSHAQQVIPSEENGENVEM-RKVQGN-QLKADSLSTISE
```

op4

op4							
B. lanceolatum	***B. floridae***				***B.belcheri***		**ScanProsite**[5]
Genomic scaffold in Assembly BraLan2[1]	JGI gene model (Putative allele or duplicate)[2]	NCBI gene model, Transcript and protein Accession numbers	Exon No and size[3]	Comments	Predicted gene model & scaffold in HapV2 assembly[4]	Comments	
Sc0000009<br>(4012428..4023488)<br><br>332aa<br>e1= 355<br>e2= 329<br>e3= 243<br>e4= 72	**70447***<br>scaffold_28<br>(206045[d])	**206045***<br>scaffold_22<br>(270aa)<br><br>XM_002611045<br>XP_002611091	333aa<br>e1= 355<br>e2= 329<br>e3= 243<br>e4= 75		164910+164900+164890*<br>Sc0000035<br><br>333aa<br>e1= 358<br>e2= 329<br>e3= 243<br>e4= 72		GPCR +<br>(52-303aa)<br>OPSIN –<br>**K296**is at position 293

B. floridae

>Bf_op4_1002bp
ATGGACGTGAACGTAACGGGGGGTTGGAACGGCAGCACGGCGCCATGGACAGTCACCCCGCTCGGGGAGG
GGGCCAGTCCCCTTCTCCTGCCCTACGAGAGCTTCCTTGCGGTCACGGCGGTGCTGACCATCTACGGTGT
CGGCGGGATCGTGTCCAACTCCGCCGTGCTGCTGATGTTCTGGCGGTACCCGACGCTCCGGACGCCGTTC
AACATGCTGCTGTTAAACCTGTGCTTAGGCGGGCTGATCGTGTCTATGGCCGGCATCCCCTTCTCCCTGT
CCTCCAGCATCCACGGGCGCTGGATCTGGGGCCGCAGCGGCTGCGTCTACTACGGCTTCACCAACAGTTT
CTGTGGTATCCTCTCCATGATCACACTGACGGTGATCGCCTACCAGCGCTACAAGATCACCGTGAGGCTC
CCTGGCGGCCCCAATCTGAAGTACAGCGATGTCATCAAGGCCATTGCCTTCGTCTGGATCTACTCCCTGG
TCTGGACGGCACCACCGCTCTTCGGGTGGAGCAGCTACCAGCTGGAGGGACCGAGGATCGGCTGCTCCGT
GGACTGGGCCTCCGGCACCGTCAACGACATCTCCTACATCATGGCGTTCTTCGTGTCGTGCCTCATCCTG
CCGCTAGGCGTCATCGCGGTGTGCTACATCAGGCTCTGGCAGCACATACACAAACGTGCAGGCCAGCAGC
AGAACAAGAATTCCGCCACGTCTAAAGCCGAGGGTCGGATCGGGCTGATGGTGGTGGTGCTGATCACGTG
TTTCCTCATCTGCTGGCTGCCGTACGGGCTCGTGGCCCTGGTCGTCACCTTCGGGGAGCCTCGGCTCATC
ACACCGACCTCCGCCATCGTCTGCACGCTCATGGCCAAGTCCAGCGTCATGTGGAATCCCATCATCTATG
TCGCCATGAACAAGCAGTTCGGAAGGCACCTGGTGGCCCTTCTTCGCTGCCTGCGAGACGCCGAGACTAC
TCCACAACCCACTCCAGTGTGA

>Bf_op4_333aa
MDVNVTGGWNGSTAPWTVTPLGEGASPLLLPYESFLAVTAVLTIYGVGGIVSNSAVLLMFWRYPTLRTPF
NMLLLNLCLGGLIVSMAGIPFSLSSSIHGRWIWGRSGCVYYGFTNSFCGILSMITLTVIAYQRYKITVRL
PGGPNLKYSDVIKAIAFVWIYSLVWTAPPLFGWSSYQLEGPRIGCSVDWASGTVNDISYIMAFFVSCLIL
PLGVIAVCYIRLWQHIHKRAGQQQNKNSATSKAEGRIGLMVVVLITCFLICWLPYGLVALVVTFGEPRLI
TPTSAIVCTLMAKSSVMWNPIIYVAMNKQFGRHLVALLRCLRDAETTPQPTPV

B. belcheri

>Bb_op4_1002bp
ATGGATGTGAACATGTCCGGGAGTTGGAACGGGAGCGGCCCGGCGGTGTGGAGCGTCACCCCGCGCGGGG
AGGCAGACGGGGCCCCGCTCCTGCCCTACCAGGGCTTCCTGGCCGTGTCTGCGGTGCTGGGGACGTTCGG
CGTCGGCGGCATCGTCTCCAACTTCACGGTGCTGCTGATGTTCTGGCAGTACCCGACCCTCCGCACGCCG
TTCAACATGCTGCTGATGAACCTGTGTCTGAGCGGGCTGATCGTGTCCGTGGCCGGCATTCCCTTCTCCC
TGGCCTCCGCTATCCACGGCCAATGGATCTGGGGCAGGGCTGGCTGTGTGTACTACGGTTTCACCAACAG
TTTCTGCGGTATTCTATCCATGATAACTCTGACGGTCATCGCCTACCAGCGCTACAAGATCACCATGCGC
CCCCCGGGAGCCCCCAGTCTGAAGTACCCAGATGTGACCAAGGCCATCGCCTTTATCTGGGTCTACTCCA
TCTGCTGGACCGTACCACCGCTGTTCGGGTGGAGCAGCTACCAGCTGGAGGGACCGAGGATAGGCTGCTC
CGTGGACTGGAGCTCGGGCACCGTCAACGGCATCTCCTACATCATGGCGTTCTTCCTGTCGTGCCTGATC
CTACCGCTGGGTGTCATTGCCGTGTGCTACGTCAGGCTCTGGCAGCACGTACACAAGCGGGCAGGCCAGC

AGCACAACAAGAAGTCTGTGGCGAGCAAGGCGGAGGGTCGGATCGGGCTGATGGTGGTGGTGCTCATCAC
GTGTTTCCTGCTGTGCTGGCTGCCGTACGGGCTCGTGGCGCTCATCGTGGCGTTCGGGAAGCCGCAGCTC
ATCACCCCGACCGCCGCCATCGTCTGCACGCTCATGGCCAAGTCCAGCGTCATGTGGAACCCCGTCATCT
ACGTCGTCATGAACAACCAGTTCCGGAGGCACCTGCTGGCCCTGCTCCGCTGTCTGCGGGACGCCGAGAC
CTCTGGACCAACTCCGGTCTGA

>Bb_op4_333aa
MDVNMSGSWNGSGPAVWSVTPRGEADGAPLLPYQGFLAVSAVLGTFGVGGIVSNFTVLLMFWQYPTLRTP
FNMLLMNLCLSGLIVSVAGIPFSLASAIHGQWIWGRAGCVYYGFTNSFCGILSMITLTVIAYQRYKITMR
PPGAPSLKYPDVTKAIAFIWVYSICWTVPPLFGWSSYQLEGPRIGCSVDWSSGTVNGISYIMAFFLSCLI
LPLGVIAVCYVRLWQHVHKRAGQQHNKKSVASKAEGRIGLMVVVLITCFLLCWLPYGLVALIVAFGKPQL
ITPTAAIVCTLMAKSSVMWNPVIYVVMNNQFRRHLLALLRCLRDAETSGPTPV

```
                                                             TM I
                                                        ######################
Bl_op4_332aa    1 MDLNMTDGWNTS-TAPWTVTPLGEVDDHPLLPYRGFIVVAAVLGTLCAGGIVSNFAVLLM
Bf_op4_333aa    1 MDVNVTGGWNGS-TAPWTVTPLGEGASPLLLPYESFLAVTAVLTIYGVGGIVSNSAVLLM
Bb_op4_333aa    1 MDVNMSGSWNGSGPAVWSVTPRGEADGAPLLPYQGFLAVSAVLGTFGVGGIVSNFTVLLM

                                      TM II                                 TM
                              ######################                ##########
Bl_op4_332aa   60 FWQYPTLRTPFNMLLLNLSVGGLIVSVAGIPFSLSSSIYGRWIWGRGGCVYYGFTNSFCG
Bf_op4_333aa   60 FWRYPTLRTPFNMLLLNLCLGGLIVSMAGIPFSLSSSIHGRWIWGRSGCVYYGFTNSFCG
Bb_op4_333aa   61 FWQYPTLRTPFNMLLMNLCLSGLIVSVAGIPFSLASAIHGQWIWGRAGCVYYGFTNSFCG

                      III                                  TM IV
                  ############                   #####################
Bl_op4_332aa  120ILSMITLTVIAYQRYKITVRPPGAANLKYHDVTKAIVFIWIYSFVWTAPPLFGWSSYQLE
Bf_op4_333aa  120 ILSMITLTVIAYQRYKITVRLPGGPNLKYSDVIKAIAFVWIYSLVWTAPPLFGWSSYQLE
Bb_op4_333aa  121 ILSMITLTVIAYQRYKITMRPPGAPSLKYPDVTKAIAFIWVYSICWTVPPLFGWSSYQLE

                                       TM V
                                     ######################
Bl_op4_332aa  180GPRVGCSVDWSSHTGNDISYIMAFFVTCLILPLGVIAVCYVRLWKHVHERAGQQHNKKSV
Bf_op4_333aa  180 GPRIGCSVDWASGTVNDISYIMAFFVSCLILPLGVIAVCYIRLWQHIHKRAGQQQNKNSA
Bb_op4_333aa  181 GPRIGCSVDWSSGTVNGISYIMAFFLSCLILPLGVIAVCYVRLWQHVHKRAGQQHNKKSV

                                      TM VI                         TM VII
                             ######################            ########*######
Bl_op4_332aa  240ASKAEGRIGMMVVVLITCFLLCWLPYGLVALVVTFGEPRLITPTAAIVCTLMAKSSVMWN
Bf_op4_333aa  240 TSKAEGRIGLMVVVLITCFLICWLPYGLVALVVTFGEPRLITPTSAIVCTLMAKSSVMWN
Bb_op4_333aa  241 ASKAEGRIGLMVVVLITCFLLCWLPYGLVALIVAFGKPQLITPTAAIVCTLMAKSSVMWN


                  #######
Bl_op4_332aa  300PVIYVVMNNQFRRHLVALLRCLRNAETTG-PTPV
Bf_op4_333aa  300 PIIYVAMNKQFGRHLVALLRCLRDAETTPQPTPV
Bb_op4_333aa  301 PVIYVVMNNQFRRHLLALLRCLRDAETSG-PTPV
```

op5

op5							
B. lanceolatum	***B. floridae***				***B.belcheri***		**ScanProsite**[5]
Genomic scaffold in Assembly BraLan2[1]	JGI gene model (Putative allele or duplicate)[2]	NCBI gene model, Transcript and protein Accession numbers	Exon No and size[3]	Comments	Predicted gene model & scaffold in HapV2 assembly[4]	Comments	
Sc0000170<br>(495358..501839)<br><br>375aa<br>e1= 364<br>e2= 182<br>e3= 150<br>e4= 234<br>e5= 198	**84890***<br>scaffold_136<br>(185357[a] scaffold_52)	**185357***<br>scaffold_245<br>(272aa)<br><br>XM_002589167<br>XP_002589213	375aa<br>e1= 364<br>e2= 182<br>e3= 150<br>e4= 234<br>e5= 198	Scaffold retained in NCBI is not the correct one – last exon is not predicted in NCBI	048470<br>Sc0000452<br><br>376aa<br>e1= 364<br>e2= 182<br>e3= 150<br>e4= 234<br>e5= 201		GPCR +<br>(55-304aa)<br>OPSIN -<br>**K296** is at position 294

B. floridae

```
>Bf_op5_1128bp
ATGGCGTCGGCGGTGCAAAACGGGACTTTTCCAGCCATGGACACCATGGCACCAACACCTGAAGCGCTAA
CTTCAGATCCCACCACCCCAGCTTACTTCACCACCGAACAACATCTCCTCATGGCCGTTTGGCTCGGTTT
TATCGGATCGTTTGGCTTCGTCGCCAACCTCCTGACGGTCCTGGTGTTCTGGTGTTTCAAGTCCCTCCGC
ACGCCCTTCCACCTGTACCTGGGCGGCATCGCGCTGAGCGACTTGCTGGTGGCGGCGCTGGGCAGCCCCT
TCGCCGTGGCGTCTGCGGTGGGAGAGCGGTGGCTGTTCGGGCGGGCGGCGTGCGTTTGGTACGCCTTCGT
GAACTACTTCCTCAGTATCGTGTCCATCGTTACCATGGCAACCATGTCGTTCTCGCGGTACTGGATCATC
ATCAGGCCGCAGTCCGCTCCACGACTGGACACGGTGTACGGCGCATGCGTGGTCAACGCCTTCGCATGGT
GCTACTCGTTCTTCTGGACCATCATGCCCGTGTTGGGCTGGAGCCGATTCACACAGGTTGCGGCCATGAC
CGTGTGCTCTCTGGACTGGGACCACCACACCCCCCTGAGTAAGTCGTACATCCCGGTGGCCTTCCTGACA
TGCCTGTTCCTGCCGCTAGGTGTCATCATCTTCAGCGTCTTCAAGACAACCATGCATCTGCGACGGGCTG
CGGAGGTTGAAGATGAGGTGCCGAACGAGGTCCGGGCTGGGCGGAAGACCACGAGAATCACCCTGGTCAT
GGCCGGCTGCTGGCTGGTCGCCTGGCTGCCGTACGCCTGCATGGCGCTAGTCATCGCCGCCGGGGGACGG
GTCTCTCCTACCGTGGAGGTCCTGGCCACCAAGTTCGCCAAGACAAGCTACATCGTGAACACCATCATCT
ATTTAGTCATGGAAAAGGAGTTCCGCAAGAGTCTTGTCCTTCTGCTGTTCTGTGGACGAGACCCTTTCGA
CATCCAGATCGAGCAGCCGGCGTACGAGAAGGCAGACGTGTACGTGGAGCGGCTGGTAACGGCGGAGCCA
ATGGTGGAGATGGAAGCCGTTAACGTCCGCCCGGCCCAGCAGGAACCAGCCAGAGCACCGTTCGGAACTC
CGCTGTGA
```

```
>Bf_op5_375aa
MASAVQNGTFPAMDTMAPTPEALTSDPTTPAYFTTEQHLLMAVWLGFIGSFGFVANLLTVLVFWCFKSLR
TPFHLYLGGIALSDLLVAALGSPFAVASAVGERWLFGRAACVWYAFVNYFLSIVSIVTMATMSFSRYWII
IRPQSAPRLDTVYGACVVNAFAWCYSFFWTIMPVLGWSRFTQVAAMTVCSLDWDHHTPLSKSYIPVAFLT
CLFLPLGVIIFSVFKTTMHLRRAAEVEDEVPNEVRAGRKTTRITLVMAGCWLVAWLPYACMALVIAAGGR
VSPTVEVLATKFAKTSYIVNTIIYLVMEKEFRKSLVLLLFCGRDPFDIQIEQPAYEKADVYVERLVTAEP
MVEMEAVNVRPAQQEPARAPFGTPL
```

B. belcheri

```
>Bb_op5_1131bp
ATGGAGTCGGTTGGGCAGAACGGGACGTTTCCAGACACCATGGCACCAACACCTGAAGCCCTGCTGACGT
CCGATCCCACCGCAACCCCGGCCTACTTCACCACGGAGCAACACCTGCTCATGGCCGTCTGGCTCGGCTT
CATCGGGTCGTTCGGCTTCATCGCCAACCTCCTGACGGTGCTGGTGTTCTGGTGCTTCAGGTCTCTCCGC
ACGCCGTTCCACCTGTACCTGGGCGGCATCGCGCTGAGCGACGTGCTGGTGGCGGCGCTTGGCAGCCCCT
TCGCCGTGGCGTCCGCGGTGGGCGAGCGGTGGCTGTTCGGCCGCGCGGCGTGCGTCTGGTACGCTTTCGT
```

```
CAACTACTTCCTCAGTATCGTGTCTATCGTGACCATGTCGGCCATGTCGTTCTCGCGATACTGGCTCATC
ATCAGACCGCAGTCCGCTCAGAGTCTGGAGACCGTGTTCGGCGCATGCGCGGTGAATGCCCTGGCGTGGT
GCTACTCGTTCTTCTGGACCATCATGCCCGTGTTGGGCTGGAGCCGGTTCACACATGTGGCAGCCATGAC
GGTGTGTTCCCTGGACTGGGATCACCACACCCCCCTGAGTAAGTCGTACATCCCGGTGGCTTTCCTGTCC
TGCCTGTTCCTACCGCTAGGTGTCATCATCTTCAGCGTCGCCAAGACAACCATGCATCTACGACGGGCTG
CCGAAGTTGAAGACGAGGTCCCAACTGAGGTGCAGGCCGGGCGTAAGACCACGAGAATCACCCTGGTGAT
GGCGGGGTGCTGGCTGGTGGCCTGGCTGCCCTACGCCTGCATGGCGCTCGTCATCGCCGCCGGAGGACAC
GTGTCCCCTACCATAGAGGTCCTGGCCACCAAGTTCGCCAAGACAAGCTACATCGTCAACACCATCATCT
ATGTAGTCATGGACAAGGAGTTCCGCAAGAGCCTGGTTCTCCTGTTGTTCTGCGGACGGGACCCGTTCAA
CATCCAGATCGAGCAGCCAGCGTACGAGAAGGCTGACGTGTACGTGGAGCGGCTGGTGACGGCCGAGCCC
ATGGTGGAGATGGAACCCGTGAACCTCCGCCAACAGGGGCAGCAGGAGCCTGGCAGAGAACCGTTCGGAA
CTCCGCTGTGA
```

```
>Bb_op5_376aa
MESVGQNGTFPDTMAPTPEALLTSDPTATPAYFTTEQHLLMAVWLGFIGSFGFIANLLTVLVFWCFRSLR
TPFHLYLGGIALSDVLVAALGSPFAVASAVGERWLFGRAACVWYAFVNYFLSIVSIVTMSAMSFSRYWLI
IRPQSAQSLETVFGACAVNALAWCYSFFWTIMPVLGWSRFTHVAAMTVCSLDWDHHTPLSKSYIPVAFLS
CLFLPLGVIIFSVAKTTMHLRRAAEVEDEVPTEVQAGRKTTRITLVMAGCWLVAWLPYACMALVIAAGGH
VSPTIEVLATKFAKTSYIVNTIIYVVMDKEFRKSLVLLLFCGRDPFNIQIEQPAYEKADVYVERLVTAEP
MVEMEPVNLRQQGQQEPGREPFGTPL
```

```
                                                                    TM I
                                                              ################
Bl_op5_375aa    1 MASVGQNGTFSAMDTMAPTPEALT--SDPTTPAYFTTEQHLLMAVWLGFIGSFGFVANLL
Bf_op5_375aa    1 MASAVQNGTFPAMDTMAPTPEALT--SDPTTPAYFTTEQHLLMAVWLGFIGSFGFVANLL
Bb_op5_376aa    1 MESVGQNGTFP--DTMAPTPEALLTSDPTATPAYFTTEQHLLMAVWLGFIGSFGFIANLL

TM II
                  ######          ######################                ######
Bl_op5_375aa   59 TVLVFWCFKSLRTPFHLYLGGIALSDLLVAALGSPFAVASAVGERWLFGRAACVWYAFVN
Bf_op5_375aa   59 TVLVFWCFKSLRTPFHLYLGGIALSDLLVAALGSPFAVASAVGERWLFGRAACVWYAFVN
Bb_op5_376aa   59 TVLVFWCFRSLRTPFHLYLGGIALSDVLVAALGSPFAVASAVGERWLFGRAACVWYAFVN

                   TM III                                      TM IV
                  ################                   ######################
Bl_op5_375aa  119YFLSIVSIVTMATMSFSRYWLIIRPQSAPRLDSLFGACVVNAFAWCYSFFWTIMPVLGWS
Bf_op5_375aa  119 YFLSIVSIVTMATMSFSRYWIIIRPQSAPRLDTVYGACVVNAFAWCYSFFWTIMPVLGWS
Bb_op5_376aa  119 YFLSIVSIVTMSAMSFSRYWLIIRPQSAQSLETVFGACAVNALAWCYSFFWTIMPVLGWS

                                                  TM V
                                          ######################
Bl_op5_375aa  179RFTQVAAMTVCSLDWDHHTPLSKSYIPVAFLTCLFLPLGVIIFSVVKTTMHLRRAAEVED
Bf_op5_375aa  179 RFTQVAAMTVCSLDWDHHTPLSKSYIPVAFLTCLFLPLGVIIFSVFKTTMHLRRAAEVED
Bb_op5_376aa  179 RFTHVAAMTVCSLDWDHHTPLSKSYIPVAFLSCLFLPLGVIIFSVAKTTMHLRRAAEVED

                                        TM VI                       TM VII
######################           ####*#######
Bl_op5_375aa  239 EVPNEVRVGRKTMRITLVMAGCWLVAWLPYACMALVIAAGGQVSPTVEVLTTKFAKTSYI
Bf_op5_375aa  239 EVPNEVRAGRKTTRITLVMAGCWLVAWLPYACMALVIAAGGRVSPTVEVLATKFAKTSYI
Bb_op5_376aa  239 EVPTEVQAGRKTTRITLVMAGCWLVAWLPYACMALVIAAGGHVSPTIEVLATKFAKTSYI

                  ##########
Bl_op5_375aa  299 VNTIIYFVMEKEFRKSLVLLLFCGRDPFDIQVDQPAYEKADVYVERLVTAEPMVEMEPVD
Bf_op5_375aa  299 VNTIIYLVMEKEFRKSLVLLLFCGRDPFDIQIEQPAYEKADVYVERLVTAEPMVEMEAVN
Bb_op5_376aa  299 VNTIIYVVMDKEFRKSLVLLLFCGRDPFNIQIEQPAYEKADVYVERLVTAEPMVEMEPVN


Bl_op5_375aa  359 LRPR-QQEPAREPFGTPL
Bf_op5_375aa  359 VRPA-QQEPARAPFGTPL
Bb_op5_376aa  359 LRQQGQQEPGREPFGTPL
```

op6

op6							
B. lanceolatum	***B. floridae***				***B.belcheri***		**ScanProsite[5]**
Genomic scaffold in Assembly BraLan2[1]	JGI gene model (Putative allele or duplicate)[2]	NCBI gene model, Transcript and protein Accession numbers	Exon No and size[3]	Comments	Predicted gene model & scaffold in HapV2 assembly[4]	Comments	
Sc0000005 (Orthologous scaffold)<br><br>op6ortholog is not present	**210643*** **(73041[m])** scaffold_42	**210643*** scaffold_6 **(299aa)**<br><br>XM_002613091 XP_002613137	325aa e1= 145 e2= 193 e3= 98 e4= 87 e5=260 e6= 195		Scaffold48‡(Orthologous scaffold)<br><br>op6ortholog is not present		GPCR + (55-315aa) OPSIN + (299-315aa)

B. floridae

>Bf_op6_978bp
ATGGCCACCACGCCGGCAGACCGGCTGGACGGCCTCACACCGGCGGGCCGGGGCGCGACCACCGCCGAGA
CGCACGCCGACGACTTCGCCTCCAAGCTATCGAGGGAAGCCGATATCGTCATCGGCGTGTATCTCATACT
CATAGGTACCGGCGCCATCCTGGGGAACGGGCGGGTGCTGTGGCTCTCCTACCGGTGCAGGGCCAGGCTG
CGGCCGGTGGAGATGTTCGTGGTGAGCCTGGCGGTGGCGGACGTGGGACTCTCCCTGGTCGGCCACCCGT
TCGCCGCCGCCTCCAGCCTGATGGGCCGCTGGTCCTTCGGCTCAGCCGGATGCACCTGGTATGGCTTCGT
TGTGTTTTTCTTGGGCATCGCCAGTATCGCCAGCATGACGCTCATGAGCATTATGCGCTTCATGATTGTC
TACAAGAGATACCCGGGCCAGTACCCCACCCGCCGGGCCAGCTGCGTCCTGGTCACCGCCGCATGGCTAT
ACGGGCTCTTCTGGGCATGCGCACCACTGGCAGGCTGGAGTCAGTACCAACCGGAGCCGTATGGCCTGTC
GTGCAGCGTGGACTGGGGCGGCTTCAGCAGTGACGCCGGCGGCAGCTCCTTCATCATCTGCATGCTCCTC
TTCTGTACCGCCGTCCCTGTCGTCATCATGGTGACGTCATATGCCGCCATCTTTGTCATCTACAGACAGG
CGCAGAAGGGCGTGGTCCTGAACCTGCAGGTTAACGCTACGTTCGGCGGGAAGAGGCAGAGGACGGAGAG
GAAGCTCACGCTGATAGCGCTGGCCGTGTGTGGCGGGTTCCTACTGGCCTGGCTGCCCTACGCAGTAGTG
GGGCTGTGGGCCAGTGTCGCCGGGGTAGATGCAGTACCGCTCGCCCTCGCTAGTGCGGCGCCCCTGTTCG
CCAAGAGCAACAGTCTGTGGAACCCCATCATCTACCTGGGAATGAACGAGCGCTTCAGGTCCGAATAA

>Bf_op6_325aa
MATTPADRLDGLTPAGRGATTAETHADDFASKLSREADIVIGVYLILIGTGAILGNGRVLWLSYRCRARL
RPVEMFVVSLAVADVGLSLVGHPFAAASSLMGRWSFGSAGCTWYGFVVFFLGIASIASMTLMSIMRFMIV
YKRYPGQYPTRRASCVLVTAAWLYGLFWACAPLAGWSQYQPEPYGLSCSVDWGGFSSDAGGSSFIICMLL
FCTAVPVVIMVTSYAAIFVIYRQAQKGVVLNLQVNATFGGKRQRTERKLTLIALAVCGGFLLAWLPYAVV
GLWASVAGVDAVPLALASAAPLFAKSNSLWNPIIYLGMNERFRSE

op7

op7							
B. lanceolatum	***B. floridae***				***B.belcheri***		**ScanProsite[5]**
Genomic scaffold in Assembly BraLan2[1]	JGI gene model (Putative allele or duplicate)[2]	NCBI gene model, Transcript and protein Accession numbers	Exon No and size[3]	Comments	Predicted gene model & scaffold in HapV2 assembly[4]	Comments	
Sc0000005 +<br>xfSc0029409<br>(5593502..>5607365)<br><br>aa<br>e1= 145<br>e2= 193<br>e3= 98<br>e4= 87<br>e5= 260<br><br>e6=185[#]<br>e7= Ns	**65045***<br>scaffold_6<br>(**73626**[a]<br>scaffold_45)	**65045***<br>scaffold_187<br><br>XM_002594150<br>XP_002594196	379aa<br>e1= 145<br>e2= 193<br>e3= 98<br>e4= 87<br>e5= 260<br><br>e6= 185<br>e7= 172	Exons 7+8 predicted in JGI were wrong<br><br>New final exon was predicted by Genscan and verified by PCR and sequencing	208750<br>scaffold48‡<br><br><br>379aa<br>e1= 142<br>e2= 193<br>e3= 98<br>e4= 87<br>e5a= 172<br>e5b= 88<br>e6= 185<br>e7= 175		GPCR +<br>(55-315aa)<br>OPSIN +<br>(299-315aa)

B. floridae

```
>Bf_op7_1140bp
ATGGCGACCACGCCGGGACTACCGTTGGACGGCCTCGCGCCGACGGGAAGAGGTGTGACCGCCGCCGACA
CGCTCGACGACGCCTTCGCCTCCAAGCTGTCGAGGGAAGCCGACATCGTCATCGGCGTGTATCTGCTACT
CATAGGTACCGGCTCCATCCTGGGAAACGGGCGGGTGCTGTGGCTCTCCTACCGGAACTGGGCGAAGCTG
CGGCCGGTGGAGCTGTTCGTGGTGAGCCTGGCGGTGACGGACGTGGGCATCTCGGTCTTCGGCTACCCGT
TCGCCGCCAGCTCCAGCTTGCTGGGCCGCTGGTCCTTCGGGTCTGCCGGATGTACCTGGTACGGCTTCAC
GGGTTTTTTCTTCGGCCTAACCAGCATCGCCAACATGGCGCTCATGAGCATCATGCGCTTCATGATTGTC
TACAAGGGATATCCAGGTCCGTACCCATCCCGCCGGGCCACCTCCGCCCTTATCGCCGCTGCGTGGCTGT
ACGGGCTCTTCTGGGCATGCGCACCCCTGGCAGGCTGGAGTCAGTACCATGTGGAACCGTTCGGCCTGTC
CTGCACCGTGGACTGGGGCAGCTTCAGCCGGGACGCCGGCGGCATGTCCTTCATCATCTGTCTGCTCGTC
TTCTGCGTCGCCATCCCTGTCACCGCCATCATGGCGTCATACGTCGCCATCTCAGCGATCTACAGACAGG
CCAAGAAGAGCATAGCAGGCCATCTGCAGGACAACTCCGCCATGTGCAAGAAAAGGAACAAATTGGAGAG
ATCAATCACACTGATGGCGTTGGCTGTGTGCGGCGGATTCCTGCTTGCCTGGCTTCCCTACGCAGTGGTG
GGGCTGTGGTCCGCTGTAGCTGGGGTAGATGCAGTACCACTCGCCCTCGCTAGTGCGGCGCCCCTGTTCG
CGAAGAGCAGCAGTCTGTGGAACCCCATCATCTACTTGGGAATGAACGATCGTTTCAGGTTGAACATGTG
CGCTTGCTTTGCTAAGACGTCTCGGAGCCAGACCACAGTGTATGCGCAGCAGCCGAGGATACCCATCCCC
CTGAGTCCCAGGGACCCGCTAGACAACAACCCTCCCGTGGCACCGGGACCTGCGCTGGACAGCAGACTCC
ATACATCCCACATCCAATAG
```

```
>Bf_op7_379aa
MATTPGLPLDGLAPTGRGVTAADTLDDAFASKLSREADIVIGVYLLLIGTGSILGNGRVLWLSYRNWAKL
RPVELFVVSLAVTDVGISVFGYPFAASSSLLGRWSFGSAGCTWYGFTGFFFGLTSIANMALMSIMRFMIV
YKGYPGPYPSRRATSALIAAAWLYGLFWACAPLAGWSQYHVEPFGLSCTVDWGSFSRDAGGMSFIICLLV
FCVAIPVTAIMASYVAISAIYRQAKKSIAGHLQDNSAMCKKRNKLERSITLMALAVCGGFLLAWLPYAVV
GLWSAVAGVDAVPLALASAAPLFAKSSSLWNPIIYLGMNDRFRLNMCACFAKTSRSQTTVYAQQPRIPIP
LSPRDPLDNNPPVAPGPALDSRLHTSHIQ
```

B. belcheri

```
>Bb_op7_1140bp
ATGGCGACGACCACGGCGGCGGACCTGTTGGACCAGGCGGCGGGTGGAGGCGCACCCACCGCCGAGACGG
ACGGAGACGCCTTCGCCTCCAAGCTAACGAGGGAAGCCGACATCATCATCGGCGTGTATCTCATACTCAT
```

```
AGGTACGGGGTCCACGCTGGGTAACGGGCGGGTGCTGTGGCTGTCCTACCGGAACTGGAAGAAGCTGCGG
CCGGTGGAGCTGCTGGTGGCGAGCCTGGCGGTTACCGATGTCGGCATCTCGCTGTTCGGCTACCCGTTCG
CCGCCACCTCCAGTCTGCTGGGCCGCTGGTCATTCGGCACCGCAGGGTGCACCTGGTACGGCTTCACAGG
GTTTTTCTTCGGCATCACCAGTATCGCCACCATGGCGCTCATGAGCCTTCTGCGCTTCATGATTGTCTAC
AAGGGATATCCGGGTTCGTACCCGTCCCGTCGGAAAACCGGCATCATTGTCGCCGCGGGCTGGCTGTACG
GGCTGTTCTGGGCATGTCTACCGCTGGCAGGCTGGAGCCAGTACCACGTGGAGCCGTTCGGCCTGTCCTG
CACGGTGGACTGGGGCAGCTTCAGCCGGGACGCCAACGGGATGTCCTTCATCCTCTGCATGATCATCTTC
TGCACGGCCGTCCCTGTCGCCGCCATGGTGGCGTCCTACGCCGCCATCTTCTACATCTACAGGAGGGCCA
AGAAGGGCGTGGACAGACATCTGAAGAACAACGCCGTCATGAGCCAGAAACGGAGAGAGGTGGAGAGATC
GATCACGCTGATGGCGCTGGCTGTGTGTGGCGGATTCCTGTTCGCCTGGATGCCCTATGCTGTGGTCGGG
ATGTGGTCAGCCGTCGCTGGGGCAGATGCAGTACCAATCGCTCTCGCTAGTGCGGCGCCCCTGTTCGCCA
AGAGCAGCAGTCTGTGGAACCCGATCATCTACTTGGGAATGAGCGATCGCTTCAGGTTGAACATGTGCGG
CTGCTTCGCCACGCCGACTCAGTCCCAGACGGCGGTGCACGCGCATCCGCAGCAGCCCGGAGCCCCCCTC
CCCCTCAGCCCCCGCGGCCCGCTGGTGAGTAACCTCCCCACGGCGGTGGGACCTCTGCCGGGCGCTCTCC
AGGCAACCAACATCCAGTAG
```

```
>Bb_op7_379aa
MATTTAADLLDQAAGGGAPTAETDGDAFASKLTREADIIIGVYLILIGTGSTLGNGRVLWLSYRNWKKLR
PVELLVASLAVTDVGISLFGYPFAATSSLLGRWSFGTAGCTWYGFTGFFFGITSIATMALMSLLRFMIVY
KGYPGSYPSRRKTGIIVAAGWLYGLFWACLPLAGWSQYHVEPFGLSCTVDWGSFSRDANGMSFILCMIIF
CTAVPVAAMVASYAAIFYIYRRAKKGVDRHLKNNAVMSQKRREVERSITLMALAVCGGFLFAWMPYAVVG
MWSAVAGADAVPIALASAAPLFAKSSSLWNPIIYLGMSDRFRLNMCGCFATPTQSQTAVHAHPQQPGAPL
PLSPRGPLVSNLPTAVGPLPGALQATNIQ
```

```
                                                                  TM I
######################
Bl_op7_322aa    1 MATTPGYRLDDLAPTDGGEPTAETHDDAFASKLSREADIVIGVYLILIGTGSVLGNGRVL
Bf_op7_379aa       1 MATTPGLPLDGLAPTGRGVTAADTLDDAFASKLSREADIVIGVYLLLIGTGSILGNGRVL
Bb_op7_379aa       1 MATTTAADLLD-QAAGGGAPTAETDGDAFASKLTREADIIIGVYLILIGTGSTLGNGRVL

                                           TM II
                                  #######################               ########
Bl_op7_322aa   61 WLSYRNWAKLRPVELFVVSLAVTDVGISLFGYPFAATSSLLGRWSFGSAGCTWYGFTGFF
Bf_op7_379aa      61 WLSYRNWAKLRPVELFVVSLAVTDVGISVFGYPFAASSSLLGRWSFGSAGCTWYGFTGFF
Bb_op7_379aa      60 WLSYRNWKKLRPVELLVASLAVTDVGISLFGYPFAATSSLLGRWSFGTAGCTWYGFTGFF

                       TM III                                  TM IV
                     ###############                        #######################
Bl_op7_322aa  121 FGTASIANMALMSIMRFMIVYKKYPGPYPTRPQTYVIIAIAWLYGLFWACAPLAGWSRYH
Bf_op7_379aa     121 FGLTSIANMALMSIMRFMIVYKGYPGPYPSRRATSALIAAAWLYGLFWACAPLAGWSQYH
Bb_op7_379aa     120 FGITSIATMALMSLLRFMIVYKGYPGSYPSRRKTGIIVAAGWLYGLFWACLPLAGWSQYH

                                                      TM V
                                             #######################
Bl_op7_322aa  181 VEPFGLSCTVDWGGFSRGAAGMSFILCLLVFCVALPVTAVVASFAGIFVIYRHAKKGVVT
Bf_op7_379aa     181 VEPFGLSCTVDWGSFSRDAGGMSFIICLLVFCVAIPVTAIMASYVAISAIYRQAKKSIAG
Bb_op7_379aa     180 VEPFGLSCTVDWGSFSRDANGMSFILCMIIFCTAVPVAAMVASYAAIFYIYRRAKKGVDR

                                    TM VI
                                      #########################
Bl_op7_322aa  241 HLQNNPTMSKKRKKMERALTLTALAVCGGFLLAWLPYAVVGLWSAVAGVDAVPLALASAA
Bf_op7_379aa     241 HLQDNSAMCKKRNKLERSITLMALAVCGGFLLAWLPYAVVGLWSAVAGVDAVPLALASAA
Bb_op7_379aa     240 HLKNNAVMSQKRREVERSITLMALAVCGGFLFAWMPYAVVGMWSAVAGADAVPIALASAA

                          TM VII
###*############
Bl_op7_322aa  301 PLFAKSSSLWNPIIYLGTNNRF--------------------------------------
Bf_op7_379aa     301 PLFAKSSSLWNPIIYLGMNDRFRLNMCACFAKTSRSQTTVY--AQQPRIPIPLSPRDPLD
Bb_op7_379aa     300 PLFAKSSSLWNPIIYLGMSDRFRLNMCGCFATPTQSQTAVHAHPQQPGAPLPLSPRGPLV


Bl_op7_322aa      ---------------------
Bf_op7_379aa     359 NNPPVAPGPALDSRLHTSHIQ
```

```
Bb_op7_379aa      360 SNLPTAVGPLPGA-LQATNIQ
```

op8

op8							
B. lanceolatum	***B. floridae***				***B.belcheri***		**ScanProsite**[5]
Genomic scaffold in Assembly BraLan2[1]	JGI gene model (Putative allele or duplicate)[2]	NCBI gene model, Transcript and protein Accession numbers	Exon No and size[3]	Comments	Predicted gene model & scaffold in HapV2 assembly[4]	Comments	
Sc0000019<br>(1226015..1261607)<br><br>366aa<br>e1= 88<br>e2= 208<br>e3= 83<br>e4= 165<br>e5= 164<br>e6= 393	**94083***<br>scaffold_247	**94083***<br>scaffold_266<br><br>XM_002587782<br>XP_002587828	377aa<br>e1= 91<br>e2= 208<br>e3= 83<br>e4= 165<br>e5= 164<br>e6= 423	Part of the fifth exon is masked by a string of Ns. Complete sequence was determined by PCR and sequencing	309970+309990+310000*<br>Sc0000349<br><br>375aa<br>e1= 88<br>e2= 208<br>e3= 83<br>e4= 165<br>e5= 170<br>e6= 414	Repeated region causes problem in the database prediction	GPCR +<br>(37-291aa)<br>OPSIN +<br>(275-291aa)

B. floridae

```
>Bf_op8_1134bp
ATGAACAACACCACCGCCGCCTTCTACCCACCGCGCCGCCAGGTCCCGGACTGGGGGTATAACGCGCTCG
GCTCATATGTTCTCTGCATCGGGTTTTGCAATCTCATCGGCAATACCTTCGTGCTCCTTGTATCTTATTA
CCGGCGCAAGAAGATCAAACCTCCCGAGGTCTTCACTATTAACCTAGCGGTCAGTGACCTGCTGTTGTTG
CTGTGTGCCTATCCGTGGATGGTGGCGTCTAGCTTCTCTCACGGATGGCAGTTTGGGGACGCAGGATGCA
TCTCATACGCCTTTTTCAGGTTCATGTTGGGACTGGTGAACATCGCCGACATCACTGTTCTGGCTGTGGT
TCGCTACCTGAAGGTCTGCAGATCCAAGGAAGTGGCGGACATGTCGTGGACATCAGCGTTGATGGTTGTA
GCGGGAACCTGGATGTTCGGAGCCTTTTGGGCGCTGATGCCGGTGTGTGGCTGGAGCCGATATACCCTAG
AACCCTTCGATGTTGCCTGCACCCTGGACTATAAAACCGCTCAGGAGAGCCAAGAAGGTGCCGCCTTCAT
GATGATTCTGTTCACCTTCTGCTTCTTCATTCCTGTCGCCGTCATCACTTTCTGCTACGTCTGCATCGTC
CGCACCgtGCGCTCCTCGCGGCAGGCCCTGAACTGCAGCAACACGAACGACAAGAATGGGGTGGAGAAAA
AGCTAACAAAGATTGCCGTGATGGTGGGTGTGGGTTTCATCCTTGCCTGGACCCCCTACTCTCTGGTCTC
CCTCTACGCCACATTCGGCGACCTGAAGTCCCTGCCGATCATCTACAGCATCCTGCCCAGCATGTTCGCC
AAGTCCGCCAGCATCTACAACCCCATCATCTACTTCTTCATGAACGACTCCTTCCGCCGTGATGTCACCG
CTCTCATCAAGTTAAGCATCCCCAAGCCGGGCGGTTCGACGAGCTGTTCGCACAGCGCGTGGGAGAAGAC
ACCGACGAGCGGGCGGGGTACCTTCATGACGTCCCCGCAGCGCATAAAACTGTCCGAGATGCGCCCGACT
GAGCCTTCGCAGGCTAAGACAACAACCACCACTGCTCTCATCACCCAGGTTGAAGGGCGCGAGGCCCCAT
CCACCTGGGTGTAA
```

```
>Bf_op8_377aa
MNNTTAAFYPPRRQVPDWGYNALGSYVLCIGFCNLIGNTFVLLVSYYRRKKIKPPEVFTINLAVSDLLLL
LCAYPWMVASSFSHGWQFGDAGCISYAFFRFMLGLVNIADITVLAVVRYLKVCRSKEVADMSWTSALMVV
AGTWMFGAFWALMPVCGWSRYTLEPFDVACTLDYKTAQESQEGAAFMMILFTFCFFIPVAVITFCYVCIV
RTVRSSRQALNCSNTNDKNGVEKKLTKIAVMVGVGFILAWTPYSLVSLYATFGDLKSLPIIYSILPSMFA
KSASIYNPIIYFFMNDSFRRDVTALIKLSIPKPGGSTSCSHSAWEKTPTSGRGTFMTSPQRIKLSEMRPT
EPSQAKTTTTTALITQVEGREAPSTWV
```

B. belcheri

```
>Bb_op8_1128bp
ATGAACAACACCACCGCCTTCCACCCGCCGCGGCACGCGGTGCCGGGCTGGGCCTTTAACGTGCTCGGCA
CATATGTTCTCTGTATAGGATTATGCAATCTCATCGGCAACACGTTCGTGCTGCTGGTGTCGTATTACCG
GCGCAAGAAGATCAAGCCTCCCGAGGTCTTCACCATTAACCTGGCGGTCAGTGACCTGATGCTGCTCCTG
TGTGCCTACCCGTGGATGGTGGCGTCAAGCTTTAACCACGGCTGGCTGTTCGGGGACGCAGGCTGTATTT
CTTACGCATTCTTCAGGTTCATGCTGGGCCTGGTGAACATCGCCAACATCACTGCTCTGGCTTTGGTTCG
GTACCTGAAGGTCTGCAGGTCAAAGCAAGTGACGGACATGTCGTGGACGTCAGCGTGGCTGAGCCTGGCA
```

```
GGGACCTGGTTGTTCGGAGCCTTCTGGGCGTTGATGCCGGTGTGCGGCTGGAGCCGGTACACCCGGGAAC
CGTTCGATGTCGCCTGCACGCTGGACTATAAAACCGCGCAGGACAGCCGCGAAGGCGCGGCCTTCATGAT
GACTCTGTTCGCCTTCTGCTACTTCATCCCTGTCGCCATCATCACCTTCTGCTACATCTGCATCGTCCGC
ACCGTGCGCTCCTCGCGGCAATCCCTGAACTGCAGCAACTCCAGCACGGACGACAAGCGCGGGGCAGAGA
AGAAGTTGACTAAGATCGCGGTGATGGTGGGTGTGGGGTTCATCCTGGCCTGGACGCCGTACTCGCTCGT
CTCGCTGTACGCCACGTTCGGCGACCTGCCGTCCCTGCCGGTCATCCTCAGCATCCTGCCCAGCATGTTC
GCCAAGTCCGCCAGCATCTACAACCCCATCATCTACTTCTTCATGAACGACTCCTTCCGCCGGGACGTCA
CCGCCATGATCAAGTTCAGCATCCCCAAGCCGGGATCGACCACAGGTTCGCAGAGCGCCTGGGAGAAGAC
TCCGACCGGCCGACATCTCACCTCCCCGCAGCGCCTCATGATGTCCGAGATGGGCTCGTCCAAGCGTAGT
GATTCGCCAGCCAAGACCACAACCACCGCACTCGTCACCCAGGTGGACGGGCACGAAGCCCCATCTACCT
GGGTGTAA
```

```
>Bb_op8_375aa
MNNTTAFHPPRHAVPGWAFNVLGTYVLCIGLCNLIGNTFVLLVSYYRRKKIKPPEVFTINLAVSDLMLLL
CAYPWMVASSFNHGWLFGDAGCISYAFFRFMLGLVNIANITALALVRYLKVCRSKQVTDMSWTSAWLSLA
GTWLFGAFWALMPVCGWSRYTREPFDVACTLDYKTAQDSREGAAFMMTLFAFCYFIPVAIITFCYICIVR
TVRSSRQSLNCSNSSTDDKRGAEKKLTKIAVMVGVGFILAWTPYSLVSLYATFGDLPSLPVILSILPSMF
AKSASIYNPIIYFFMNDSFRRDVTAMIKFSIPKPGSTTGSQSAWEKTPTGRHLTSPQRLMMSEMGSSKRS
DSPAKTTTTALVTQVDGHEAPSTWV
```

```
                        TM I
                              ########################        ######
Bl_op8_366aa    1 MNNTTA-YHPPRPVLPGWGFDALGVYVLCIGFCNLVGNMFVLLVSYYRRKKIKPPEVFTV
Bf_op8_377aa    1 MNNTTAAFYPPRRQVPDWGYNALGSYVLCIGFCNLIGNTFVLLVSYYRRKKIKPPEVFTI
Bb_op8_375aa    1 MNNTTA-FHPPRHAVPGWAFNVLGTYVLCIGLCNLIGNTFVLLVSYYRRKKIKPPEVFTI

TM IITM III
                  ##################                ######################
Bl_op8_366aa   60 NLAVCDLMLLICAYPWMVASSFSHGWLFGEAGCISYAFFRFMLGLVNIANITALAVVRYL
Bf_op8_377aa   61 NLAVSDLLLLLCAYPWMVASSFSHGWQFGDAGCISYAFFRFMLGLVNIADITVLAVVRYL
Bb_op8_375aa   60 NLAVSDLMLLLCAYPWMVASSFNHGWLFGDAGCISYAFFRFMLGLVNIANITALALVRYL

                                    TM IV
                                ######################
Bl_op8_366aa  120KVCRSKEVTDMSWTSGWVSLAGTWMFGAFWALMPVCGWSRYTREPFDVACTLDYKTARES
Bf_op8_377aa  121 KVCRSKEVADMSWTSALMVVAGTWMFGAFWALMPVCGWSRYTLEPFDVACTLDYKTAQES
Bb_op8_375aa  120 KVCRSKQVTDMSWTSAWLSLAGTWLFGAFWALMPVCGWSRYTREPFDVACTLDYKTAQDS

                              TM V
                       ######################                        #
Bl_op8_366aa  180QEGAAFMMTLFAFCYFIPVAIISFCYSCIVRTVRSSRRALNCST--IRDRSGAEKKLTKI
Bf_op8_377aa  181 QEGAAFMMILFTFCFFIPVAVITFCYVCIVRTVRSSRQALNCSN--TNDKNGVEKKLTKI
Bb_op8_375aa  180 REGAAFMMTLFAFCYFIPVAIITFCYICIVRTVRSSRQSLNCSNSSTDDKRGAEKKLTKI

TM VI                         TM VII
                  #####################             ########*#############
Bl_op8_366aa  238 AVMVGVGFILAWTPYSLVSIYATFRDLESIPVILSILPSMFAKSASIYNPIIYFFMNDSF
Bf_op8_377aa  239 AVMVGVGFILAWTPYSLVSLYATFGDLKSLPIIYSILPSMFAKSASIYNPIIYFFMNDSF
Bb_op8_375aa  240 AVMVGVGFILAWTPYSLVSLYATFGDLPSLPVILSILPSMFAKSASIYNPIIYFFMNDSF


Bl_op8_366aa  298 RRDVTALIKLSIPKAG-TTSCSQTAWENTPTGR---NCPQHMEL-------CETHLSPAK
Bf_op8_377aa  299 RRDVTALIKLSIPKPGGSTSCSHSAWEKTPTSGRGTFMTSPQRIKLSEMRPTEPSQ-AKT
Bb_op8_375aa  300 RRDVTAMIKFSIPKPG-STTGSQSAWEKTPTGR---HLTSPQRLMMSEMGSSKRSDSPAK


Bl_op8_366aa  347 TTTTALITHVDGREAPSTWV
Bf_op8_377aa  358 TTTTALITQVEGREAPSTWV
Bb_op8_375aa  356 TTTTALVTQVDGHEAPSTWV
```

op9 + Amphiop2

op9							
B. lanceolatum	***B. floridae***				***B.belcheri***		**ScanProsite[5]**
Genomic scaffold in Assembly BraLan2[1]	JGI gene model (Putative allele or duplicate)[2]	NCBI gene model, Transcript and protein Accession numbers	Exon No and size[3]	Comments	Predicted gene model & scaffold in HapV2 assembly[4]	Comments	
Sc0000182<br>(210518..>220518)<br><br>616aa<br>e1= 97<br>e2= 197<br>e3= 100<br>e4= 97<br>e5= 150<br>e6= 79<br>e7= 171<br>e8= 960	**71561**<br>scaffold_34<br>(110962[a]<br>scaffold_818)	**71561**<br>scaffold_98<br><br>XM_002604100<br>XP_002604146	576aa<br>e1= 100<br>e2= 197<br>e3= 100<br>e4= 97<br>e5= 150<br>e6= 79<br>e7= 171<br>e8= 837		153830/044820F*<br>Sc0000013<br><br>507aa<br>e1= 94<br>e2= 197<br>e3= 100<br>e4= 97<br>e5= 150<br>e6= 79<br>e7= 171<br>e8= 636		GPCR +<br>(40-293aa)<br>OPSIN –<br>**K296** is at position 283

B. floridae

```
>Bf_op9_1731bp
ATGGCCTCCACCAACAATACCACCAACGATAGCGAGTGGGGCCCGCAGGAAACGCTTGGCGTGTCAGCCA
CTATCATGGGTATATACCTCACCGTAGTAGGTCTGGTGTCCACCGTCGGTAACGCTACCGTGGTGCTGAT
GTTCATGCTGAAGTGGAGACAGCTCTGTCGCAAGGCCCCCAACCTGCTCATCATCAACCTAGCCGCAGTC
GACCTCTGCATATCCGTATTTGGGTATCCGTTTTCTGCGTCATCAGGTTTCGCCAACCAATGGCTCTTCT
CGGACGCCATCTGCACGCTGTACGGCTTCAGCTGTTTCCTGTTGTCCATGGTGAGCATGTGCACCCTGTG
TCTCATCAGCATCCACAGGTACATCACCATCTGCCGCCCTGAACATGCTAGTAAGTTGACCATGACCCGG
ACGATACTGGCTGTGGTCGGGGCCTGGGTGTACGGGATCTCCGTGGCGGTACCACCACTCTTCGGCATTG
CAAGGTATACGTACGAGTCCTTCGGTCTGTCCTGTACCATCGACTTTCACGGTACCACTGTGGCTGATAT
GGTGTACCTGAGTATCCTGATCATTCTGTGCTATGTCATCAATGTCGCCGTCATGGGGACCTGCTACTTC
AAAATCATCAGGAAGTTCTCCAAACATCGGTTCAGGGAAGTACGTGACGTCAGGACTAGTCATCAGCATA
GTTTCGAAAGGGGAGTCACTCTGCGGTGTATCCTGATGACGCTGTTCTATCTGATATCCTGGACGCCGTA
CACAGCCGTGGCGGTATGGACCATGGTCGGGCCCCCGCCGCCGGTACAGCTGGGCATGGTGGCCGCGCTG
ACCGCCAAGACTCACTGCGCCTTCAACCCCATCTTATACATGCTCATGAGCGAGGTGTACCGGAAGCTGG
TGCTCCGCACGATGTGTCCCTGTTGTTTCAACAAAATCAGCAACAAGCTTGTCAGGCTACCGGCTGACGA
CAGCAAACACTCGGGCAACCTCGACATCTTCACTGTGGGGTACAACACTCGCGACCAGGCCGTGCAGATC
AACAAAAACGCCGCCAGGCGGTTCTGTTTTGTAATGGAGACGGCGTCAGATGATTTGGGCATTGATGATG
AGGTCTTCGCGGGTCAGCTTGGGCTGTGTAGTCGAGTCAAGGCTACGGAACCCGGTGTAGAGGGTTTTGG
CGGCTCCGAAGTCCCTCAGTCGCCGTCAGGAACGGAAAGTGAATGGTCTCTCTCCCTGTTGGACTTCTTA
CCCAAGAGAAGCTCCTCGAAGACTGCAAAAGCTTCTTCGCTATCCGAAACTTGTTCTGACAATACGGTGT
TGTCATCAGCGGCAAGGAAAATGGCATTTTTGGAGTCATCACACCAACAGTCGGACAGAGAAGTCTGTTG
CATAGAGAACCGTCAGGCACCTGAAGACACGAAACCCTGCAAGTTCGCAATAGAATCGCTAGGGGTCAGG
CTTCCCCACAAGTGCTGTACAGCTTCACAGGTTGCTGGAGCCCCGAGTAGATACGCTGGGATGATCGAGA
CGTTTACAGACTCAAAAGGAAAAACGAAAAAAAAAGCAGCCGTCAGTTTGTCAGAGATTGACGTTAAAAA
GCCCCCACCAGCGTCCAAAACGTGGGAGAGAAGAAAAACAAGCAAAAACACGTCTCGCGGACAAAGGGTA
AAAAGAACGTTTGGCAAGTCTAGAAAACATGCGTATATTGTTGACTGCTAG
```

>Bf_op9_576aa
MASTNNTTNDSEWGPQETLGVSATIMGIYLTVVGLVSTVGNATVVLMFMLKWRQLCRKAPNLLIINLAAV
DLCISVFGYPFSASSGFANQWLFSDAICTLYGFSCFLLSMVSMCTLCLISIHRYITICRPEHASKLTMTR
TILAVVGAWVYGISVAVPPLFGIARYTYESFGLSCTIDFHGTTVADMVYLSILIILCYVINVAVMGTCYF
KIIRKFSKHRFREVRDVRTSHQHSFERGVTLRCILMTLFYLISWTPYTAVAVWTMVGPPPPVQLGMVAAL
TAKTHCAFNPILYMLMSEVYRKLVLRTMCPCCFNKISNKLVRLPADDSKHSGNLDIFTVGYNTRDQAVQI
NKNAARRFCFVMETASDDLGIDDEVFAGQLGLCSRVKATEPGVEGFGGSEVPQSPSGTESEWSLSLLDFL
PKRSSSKTAKASSLSETCSDNTVLSSAARKMAFLESSHQQSDREVCCIENRQAPEDTKPCKFAIESLGVR
LPHKCCTASQVAGAPSRYAGMIETFTDSKGKTKKKAAVSLSEIDVKKPPPASKTWERRKTSKNTSRGQRV
KRTFGKSRKHAYIVDC

B. belcheri

>Bb_op9_1524bp
ATGATCCCGGCCAACAACACCACGGACCACCGCGCCTGGAGCCTGGAGCACGGCGTGTCGGCCACTATCA
TGGGGGTTTACCTCACCATAGTAGGTCTGGTGGCCACCGTCGGTAACGCTACAGTGGTGCTGATGTTTAT
CCTGAAGTGGAGACAGCTCTGCCGCAAGGCGCCGAACCTGCTCGTCATCAACCTAGCCGCCGCCAACCTC
TGCGTAACCATATTTGGGTATCCCTTTTCTGCGTTATCAGGCTACGCCCACCAATGGTTATTCTCTGACG
CCATCTGCACGCTGTACGGGTTCAGCTGTTTTGTGCTGTCCATGGTGTCCATGCACACTCTGTGTCTCAT
CAGCATACACAGGTACATCACCATCTGCCGCCCTGAACACGCCAGTAAGTTGACAATGAGCAGGACAATC
TTGGCTGTGATCGGGGCCTGGCTGGTTGCGATCGTCGTGGCGGTACCACCACTGTTCGACATTGCAAGGT
ACACGTACGAGCCGTTCGGTCTGTCCTGTGCTATTGACTTCCGGGTCACGACCGTGGCTGACCTGGTGTA
CCTCATCTGCCTGATCGTCCTGTGCTACGTCATCAATGTCGTCATCATGGGAACCTGCTACTTCAAAATC
ATCAGGAAGTTCTCCAAACAGCGATTCAGACAAGTCCGTGACATCAGGACTAGTTATCGGCGCAGCTTCG
AAATGGGAGTCACCCTGCGGTGTATCCTGATGGTGCTGTTCTACCTGGTGTCCTGGACGCCCTACACGGC
CCTGTGTGTGTGGACCATGGTGGGCCCGCCGCCGCCGGTGGAGGCCAGCATGGCGGCCACCCTGATCGCC
AAGACCCACTGCGCCTTCAACCCCATCTTATACGCGTTCATGAGCGAGGTGTACCGGAAGCTGGTGTTTC
GCACCATGTGTCCGTGTTGTTTCACCAGGATCAGCTGCACTTCGGCCGCCACGCCGCCAGGGAGCAGCAA
AGTCTCCGGCCACCCCACCCCCGACATCTTCACCGTGGGCTACAGCAGCCGGGACCAGGCCGTGCAGATC
AACAGGGCCGCTTCCAGGCGCTTCTGTTTCGTTACGGAGACGGCGTCAGAAGATCTGGGCATTGAGGAAA
CGAGTTTTACGGGCCACATTGGTCTGTGGAGATCAGGGGCCACGGTAGAGGGTTTGGGCGGCGTCCAAGT
CACACAGTCGCCGACGTCAGGGTCAGGAAGTGAGTCGTCACCGTCTCTCTTGGACTTCCTACCCAAAAGG
GCTTCAGGAAGGATAGCTTCTGCAAAAGTGCCTTCCCCGTCTGCTTTTACTTTTGAGCAGACGACATGCC
CCGAGTTTATAGACAGACAGGCCGCTGGGCCGGAACAGTCTCAGCAGGACAAGACAACCCGATCATCTGC
ACTGGACGTCAGGCTGACCGAGGATGACGTCACAGATACCACACCTGCCTCGGAAACATGGCGGAGAAAG
ACGTCAAGAAGAACTTTTGGCAAGTCTAGGAAACATGCGTATATCGTTGATTAG

>Bb_op9_507aa
MIPANNTTDHRAWSLEHGVSATIMGVYLTIVGLVATVGNATVVLMFILKWRQLCRKAPNLLVINLAAANL
CVTIFGYPFSALSGYAHQWLFSDAICTLYGFSCFVLSMVSMHTLCLISIHRYITICRPEHASKLTMSRTI
LAVIGAWLVAIVVAVPPLFDIARYTYEPFGLSCAIDFRVTTVADLVYLICLIVLCYVINVVIMGTCYFKI
IRKFSKQRFRQVRDIRTSYRRSFEMGVTLRCILMVLFYLVSWTPYTALCVWTMVGPPPPVEASMAATLIA
KTHCAFNPILYAFMSEVYRKLVFRTMCPCCFTRISCTSAATPPGSSKVSGHPTPDIFTVGYSSRDQAVQI
NRAASRRFCFVTETASEDLGIEETSFTGHIGLWRSGATVEGLGGVQVTQSPTSGSGSESSPSLLDFLPKR
ASGRIASAKVPSPSAFTFEQTTCPEFIDRQAAGPEQSQQDKTTRSSALDVRLTEDDVTDTTPASETWRRK
TSRRTFGKSRKHAYIVD

```
                                                         TM I
                                                ######################
Bl_op9_616aa        1 MTPANNTTE--PSEWSPAE-LGVSATIMGVYLTIVGLVATVGNATVVLLFILKWRQLCRK
Bf_op9_576aa        1 MASTNNTTN--DSEWGPQETLGVSATIMGIYLTVVGLVSTVGNATVVLMFMLKWRQLCRK
Bb_op9_507aa        1 MIPANNTTDH--RAWS--LEHGVSATIMGVYLTIVGLVATVGNATVVLMFILKWRQLCRK
Amphiop2_BAC76020   1 MIPTNNNTENNDLEWGLEKEHGVSATIMGVYLTIVGLVATVGNATVVLMFIMKWRQLCRK

                             TM II                                 TM III
                         ######################             ##################
Bl_op9_616aa       58 APNLLIINLAAADLCITIFGYPFSASSGYAHKWLFSDAICTMYGFSCFLLSMVSMHTLCL
Bf_op9_576aa       59 APNLLIINLAAVDLCISVFGYPFSASSGFANQWLFSDAICTLYGFSCFLLSMVSMCTLCL
Bb_op9_507aa       57 APNLLVINLAAANLCVTIFGYPFSALSGYAHQWLFSDAICTLYGFSCFVLSMVSMHTLCL
Amphiop2_BAC76020  61 APNLLVINLAAANLCITIFGYPFSASSGYAHQWLFPDAICTLYGFSCFLLSMVSMHTLCL
```

```
                                                    TM IV
                         ###                   #####################
Bl_op9_616aa        118 ISVHRYITICRPEHASQLTMNRTILAVVGAWLYALTMAIPPLFNITRYKQEPFGLSCTID
Bf_op9_576aa        119 ISIHRYITICRPEHASKLTMTRTILAVVGAWVYGISVAVPPLFGIARYTYESFGLSCTID
Bb_op9_507aa        117 ISIHRYITICRPEHASKLTMSRTILAVIGAWLVAIVVAVPPLFDIARYTYEPFGLSCAID
Amphiop2_BAC76020   121 ISAHRYITICRPEHASKLTMNRTVLAVIGTWLYAIAVAVPPLFNIARYTYEPSGLSCTID

                                     TM V
                                   #######################
Bl_op9_616aa        178 FHVTSTADLVYLGVLIVLGYVINVAVMGYCYFKIVRKFSKHRFRDVRDIRTSNQHSFERG
Bf_op9_576aa        179 FHGTTVADMVYLSILIILCYVINVAVMGTCYFKIIRKFSKHRFREVRDVRTSHQHSFERG
Bb_op9_507aa        177 FRVTTVADLVYLICLIVLCYVINVVIMGTCYFKIIRKFSKQRFRQVRDIRTSYRRSFEMG
Amphiop2_BAC76020   181 FRVTTVADLVYLGSLIVLCYVIHVAVMATCYFKIIRKFSRHRFRQVRDIRTSHQRSFEMG

                                   TM VI                        TM VII
####################               #######*#############
Bl_op9_616aa        238 VTLRCILMTLLYLISWTPYTALAIWTMVGPPPPVEVGLVAALTAKTHCAFNPILYTLMSE
Bf_op9_576aa        239 VTLRCILMTLFYLISWTPYTAVAVWTMVGPPPPVQLGMVAALTAKTHCAFNPILYMLMSE
Bb_op9_507aa        237 VTLRCILMVLFYLVSWTPYTALCVWTMVGPPPPVEASMAATLIAKTHCAFNPILYAFMSE
Amphiop2_BAC76020   241 VTMRCILMTLFYLLSWTPYTAVCIWTMVGPPPPVVVSMAAALIAKTHCAFNPILYAFMSE

Bl_op9_616aa        298 VYRKLVLRTMCPCCFNRIGGNLARAPAEDSRHSG--NPDIFTVAYSSREQAVHIR--NPR
Bf_op9_576aa        299 VYRKLVLRTMCPCCFNKISNKLVRLPADDSKHSG--NLDIFTVGYNTRDQAVQINKNAAR
Bb_op9_507aa        297 VYRKLVFRTMCPCCFTRISCTSAATPPGSSKVSGHPTPDIFTVGYSSRDQAVQINRAASR
Amphiop2_BAC76020   301 VYRKLVFRTMCPCCFNRISCKFVGTPTGGSKVSA--NPDIFTVDYNSRDQAVQINKAPSR

Bl_op9_616aa        354 RFCFVMETASENLGIDDEVFTGQIVPCSQLKTGGPSVEGSGGPEVTQRPSV-SGSERSLS
Bf_op9_576aa        357 RFCFVMETASDDLGIDDEVFAGQLGLCSRVKATEPGVEGFGGSEVPQSPSG-TESEWSLS
Bb_op9_507aa        357 RFCFVTETASEDLGIEETSFTGHIGLW----RSGATVEGLGGVQVTQSPTSGSGSESSPS
Amphiop2_BAC76020   359 RFCFVMET-SEDLGSDDTGLTGHSGLW----RSGAEVEGLGGLQVTQSPSV-SGSELSLS

Bl_op9_616aa        413 LLLDVLPKRRSSSERAASSSV-RGSHNTVSSSSLRQTTCHHGSSQQPSGRKDTRTRQSHT
Bf_op9_576aa        416 L-LDFLPKRSSSKTAKASSLSETCSDNTVLSSAARKMAFLE-------------------
Bb_op9_507aa        413 -LLDFLPKRASGRIASAKVPSPSA-------FTFEQTTCPE-FID----RQAA-------
Amphiop2_BAC76020   413 -LLDFLPPKPSGRAVSAKLPSPPA-------LNSERATCPE-SSQQPSDRPAT-------

Bl_op9_616aa        472 DGKTRPTGGRVSKRSSSHFQQAFDAVAEAWREAEAMAGGYNVERQPPTDTKPTKCASESL
Bf_op9_576aa        456 ---------------SSHQQ-------------SDREVCCIENRQAPEDTKPCKFAIESL
Bb_op9_507aa        453 ------------------------------------------------------------
Amphiop2_BAC76020   457 ------------------------------------------------------------

Bl_op9_616aa        532 GSRPSNKCSTDAHDLGGSHRYMASIKTSTGTKGRAKDQSGVRWTEVDDTKLSSASKTSDG
Bf_op9_576aa        488 GVRLPHKCCTASQVAGAPSRYAGMIETFTDSKGKTKKKAAVSLSEIDVKKPPPASKTWER
Bb_op9_507aa        453 ---------------G--------PEQSQQDKTTRSSALDVRLTEDDVTDTTPASETWRR
Amphiop2_BAC76020   457 ---------------G--------LRQYQKGDTTRSSVGDLILTEDDVTNLPPASETWGR

Bl_op9_616aa        592 KKTSKT---DRTKRTVFGMSRKHAYIVD-
Bf_op9_576aa        548 RKTSKNTSRGQRVKRTFGKSRKHAYIVDC
Bb_op9_507aa        490 KT----------SRRTFGKSRKHAYIVD-
Amphiop2_BAC76020   494 KKSENPLSYRQTTRRTFGRSRKHSYIVD-
```

op10

op10							
B. lanceolatum	***B. floridae***				***B.belcheri***		**ScanProsite[5]**
Genomic scaffold in Assembly BraLan2[1]	JGI gene model (Putative allele or duplicate)[2]	NCBI gene model, Transcript and protein Accession numbers	Exon No and size[3]	Comments	Predicted gene model & scaffold in HapV2 assembly[4]	Comments	
Sc0000358 (60865..71904)<br><br>469aa<br>e1= 145<br>e2= 114<br>e3= 180<br>e4= 117<br>e5= 236<br>e6= 173<br>e7= 145<br>e8=300	**215180*<br>(76075^{m})**<br>scaffold_61	**215180***<br>scaffold_122<br>(262aa)<br><br>XM_002601660<br>XP_002601706	 <br><br>469aa<br>e1= 145<br>e2= 114<br>e3= 180<br>e4= 117<br>e5= 236<br>e6= 173<br>e7= 145<br>e8=300		061300/073780F*<br>Sc0000031<br><br>472aa<br>e1= 145<br>e2= 114<br>e3= 180<br>e4= 117<br>e5= 236<br>e6= 173<br>e7= 145<br>e8=309		GPCR +<br>(55-316aa)<br>OPSIN –<br>**K296** is at position 306

B. floridae

>Bf_op10_1410bp
ATGTTCAACAACAGCGATGGCAGCTCCAGTTCTAATGACACGCTGTCCCTGTGGCACTACGATGGGGAGT
TCCTGGATAAATCTCACATTCAACCTCTGACAACGCTCGGCTACCTGGGGACTGCCGTCTACCTGACCAT
CCTAGGTTCAATCGCCACGCTGGGGAATGGCTCTGTCATCGCTGTTTACATCCGTCAGAAAAAATTCCGC
TCCAAACCGCACAACATCCTCATCCTAAACCTGGCGGTCAGCGACCTCGGTATCTCCATATTTGGCTACC
CATTCTGCACGGCGTCCGGGTACGCTGGGTACTGGCTGTTCGGCGACACTGTGTGCCAACTGTACGCCTT
CATGTGTTATACACTGTCCATGAGCAGTCTCAACACCCTGGTCGTCATAGCCGGGTTCAGATACATCTCT
CTCTGCCACCCGCAGTATGCCTACAGGTTGACCCACCACGTGACGGCGTTCTCCCTGATCGGCGTGTGGG
TCTACAGCCTGCTGTGGACGGTACCGCCATTGGTAGGGTGGAGCAGCTACACCTACGAATTATTCGGTAC
GTCTTGTAGCATCAAGTGGACAGTAGAGAACACATCCGAGATGTCGTACGTGGTTGGGAGCTGTCTCTTC
TGCTACCTCCTCCACCTGCTCCTCTTGGGCTTTTTCTACTACAAGGTTGCGAGGCGGCTGCGGAAGCTGA
ACCTGCGGGGTCAACACGCCATGCCGGCAGTCCGGGGGAGACGGGACTTTCTCACCAGCAGCAGGATGCA
GTCGGAGTCCAAGGCCACTATGATGTGTTTCCTAATGGTGATACTGTTCATGGTGGCGTGGACTCCCTAC
ACCGTCTCATCCTTCTGGTCAGTCCTGGTGGAGGAGATCCCCCTAGCGGCCGCCACGTATCCGACAATGT
TCGCCAAGTCGTCATGTGTCTTCAACCCCATTATCTACGCCATCGCTCACAAGAAGTTCCGGACGTTCCT
GTTCCAGACGTGTTGCCCCTGGCTGTACAGAAGGGTGGGTCTGTGCAAAAGCAACCCTTACCTGGAGCAG
CAACGGAAGGACCGGCTGCGCAAACAGAAGGGACAGATTTTCTGTGTACAGTACACCAATGGGAATGTCT
ACATCGGTCCCCATGGATCACGTGGCTGCTGGTCAGAAGACGGAAGTGGTGATGGGGGAAGCAGTGGGCG
GTGCCACTACCACAGGGCGAGTGGGTCCGTCTCCACACTCCGGGTAGACAACCTGGCCAGGCGACCGCAT
GCGGGTAACGCACCGAAGACACTACACCCGAAAGGAAGGAAGTACACGGTGCAAGCGCAGGTAGAACCGG
TACCAAGAACGGACAACGACTCGTCTAGTAGCGAGGACTCCGTTCATGACTTGTCCTGTATTGACTTCAC
AGTGTTGTAG

>Bf_op10_469aa
MFNNSDGSSSSNDTLSLWHYDGEFLDKSHIQPLTTLGYLGTAVYLTILGSIATLGNGSVIAVYIRQKKFR
SKPHNILILNLAVSDLGISIFGYPFCTASGYAGYWLFGDTVCQLYAFMCYTLSMSSLNTLVVIAGFRYIS
LCHPQYAYRLTHHVTAFSLIGVWVYSLLWTVPPLVGWSSYTYELFGTSCSIKWTVENTSEMSYVVGSCLF
CYLLHLLLLGFFYYKVARRLRKLNLRGQHAMPAVRGRRDFLTSSRMQSESKATMMCFLMVILFMVAWTPY
TVSSFWSVLVEEIPLAAATYPTMFAKSSCVFNPIIYAIAHKKFRTFLFQTCCPWLYRRVGLCKSNPYLEQ
QRKDRLRKQKGQIFCVQYTNGNVYIGPHGSRGCWSEDGSGDGGSSGRCHYHRASGSVSTLRVDNLARRPH
AGNAPKTLHPKGRKYTVQAQVEPVPRTDNDSSSSEDSVHDLSCIDFTVL

B. belcheri

```
>Bb_op10_1419bp
ATGTTCAACGACAGCGCCGGTAACTCCAATACAGACGGAGAACTGCGTCTGTGGCACTACGATGGCGAGT
TCTTAGATAAATCCCACATCCAACCGCTGACTACGCACGGCTACCTGGCAACGGCCATCTACCTGACAAT
CTTAGGTTGTATCGCTACGCTGGGGAATGGCTCTGTCATCGTCGTCTACGCCCGTCAGAAAAAGTTCCGC
TCCAAACCGCACAACATTCTCATCCTCAACCTAGCGATCAGCGACCTCGGTATCTCAATATTTGGGTATC
CGTTCTGCACGGCGTCTGGGTACGCTGGGTACTGGTTGTTCGGGGACGCGGTGTGCCAGCTGTACGGCTT
CATGTGTTACACCCTGTCCATGAGCAGTCTCAACACCCTGGTCGTCATAGCCGGATTCAGATACATCTCT
CTCTGCCGCCCACAGTATGCCTACAAGTTGACCCACCGTGTGACGGCCTACTCGCTGATCGGGGTGTGGC
TGTACAGCCTGCTGTGGACGGTGCCGCCACTGGTAGGGTGGAGCAGCTACACGTATGAACTCTTCGGTAC
GTCTTGTAGTATCAAGTGGACGGTGGAGGACACCTCCGAGATGGTGTACGTCATCGGGAGCTGTCTCTTC
TGTTATCTTGTCCACCTGCTCCTCCTGGTGTTCTTCTACTACAAGATCGCCAAGCGGATGCGGAAGCTGA
ACCTGCGGGGACAACACGCCGTTCCGACCGCCAGCGGCAGGAGGGACTTCGTCACCAGCAGCAGGATGCA
GTCGGAAACTAGGGCCACTATGATGTGTTTCCTGATGGTGATACTGTTTATGGTGGCCTGGACTCCCTAC
ACCGTCTCGTCCTTCTGGTCTACACTAGTGAACGAGATCCCGCTATGGGCGGCCACATATCCCACCATGT
TCGCTAAGTCCTCCTGTGTCTTCAACCCCCTCATATACGCTGTCGCTCACAAGAAATTCCGGTCGTTCCT
GACCCAGACGTGTTGTCCTTGGCTGTACAGAAGAGTGGGCCTGGGGAAAAACAACCCATACCTGGACCAG
CAACGACAGAACAGGATACGCAAACAGAAGGGACAAATCTTCTGTGTACAATACACTCACGGCAATGTTT
ATATCGGTGCCCATGGTTCACGTGGCTGCTGGTCAGAAGACAGCGGTAGAATCCGCGGTACGTGCCGGTA
CCACAGGGCGAGGTCGTCCTTTTCCACACTCCACGTGGACAACCTGGCCAGACTGTCGCAGGGTGGGGCC
TCTTCTTCTACGGACAGTAGATTGCCGTCGGTACATCGGAAAGGGAGAAAGTACACCGTGCAAGCACAGA
TAGAACCTGCGCCTAGACAGGAGAACAGTGCGTCTAGCAGTGATGAGTCCGTCGACGACATGTCCTGTAT
TGATTTCACAGTGATATAG
```

```
>Bb_op10_472aa
MFNDSAGNSNTDGELRLWHYDGEFLDKSHIQPLTTHGYLATAIYLTILGCIATLGNGSVIVVYARQKKFR
SKPHNILILNLAISDLGISIFGYPFCTASGYAGYWLFGDAVCQLYGFMCYTLSMSSLNTLVVIAGFRYIS
LCRPQYAYKLTHRVTAYSLIGVWLYSLLWTVPPLVGWSSYTYELFGTSCSIKWTVEDTSEMVYVIGSCLF
CYLVHLLLLVFFYYKIAKRMRKLNLRGQHAVPTASGRRDFVTSSRMQSETRATMMCFLMVILFMVAWTPY
TVSSFWSTLVNEIPLWAATYPTMFAKSSCVFNPLIYAVAHKKFRSFLTQTCCPWLYRRVGLGKNNPYLDQ
QRQNRIRKQKGQIFCVQYTHGNVYIGAHGSRGCWSEDSGRIRGTCRYHRARSSFSTLHVDNLARLSQGGA
SSSTDSRLPSVHRKGRKYTVQAQIEPAPRQENSASSSDESVDDMSCIDFTVI
```

```
                                                                     TM I
                                                            ###################
Bl_op10_469aa    1 MFNSSDGKSSPNDALFLWHYDGEFLDKAHIQPLTPQGYLATAVYLTFIGCIATLGNASVI
Bf_op10_469aa    1 MFNNSDGSSSSNDTLSLWHYDGEFLDKSHIQPLTTLGYLGTAVYLTILGSIATLGNGSVI
Bb_op10_472aa    1 MFNDSAGNSNTDGELRLWHYDGEFLDKSHIQPLTTHGYLATAIYLTILGCIATLGNGSVI

                                          TM II
                   ####           #######################               #######
Bl_op10_469aa   61 AVYIRQKKFRSKPHNILILNLAASDLGISIFGYPFCTASGYAGYWLFGDAVCQLYGFMCY
Bf_op10_469aa   61 AVYIRQKKFRSKPHNILILNLAVSDLGISIFGYPFCTASGYAGYWLFGDTVCQLYAFMCY
Bb_op10_472aa   61 VVYARQKKFRSKPHNILILNLAISDLGISIFGYPFCTASGYAGYWLFGDAVCQLYGFMCY

                  TM III                                 TM IV
                   ################                  #######################
Bl_op10_469aa  121TLSMSSLNTLVVIAGFRYISLCHPQYAYRLTHRVTAYSLIGLWFYGLLWTVPPLVGWSSY
Bf_op10_469aa  121 TLSMSSLNTLVVIAGFRYISLCHPQYAYRLTHHVTAFSLIGVWVYSLLWTVPPLVGWSSY
Bb_op10_472aa  121 TLSMSSLNTLVVIAGFRYISLCRPQYAYKLTHRVTAYSLIGVWLYSLLWTVPPLVGWSSY

                                                 TM V
                                        #######################
Bl_op10_469aa  181TYEIFGTSCSIKWMVENTSEMLYVVGSCIFCYLLHLLLLAFFYYKIAKRMRKLNLRGQHT
Bf_op10_469aa  181 TYELFGTSCSIKWTVENTSEMSYVVGSCLFCYLLHLLLLGFFYYKVARRLRKLNLRGQHA
Bb_op10_472aa  181 TYELFGTSCSIKWTVEDTSEMVYVIGSCLFCYLVHLLLLVFFYYKIAKRMRKLNLRGQHA

                                                TM VI
                                           #########################
Bl_op10_469aa  241VPAARGRRDFVTSSRMKSESRATLMCFLMVILFVVAWTPYTVSSFWSILVQKIPLAAATY
Bf_op10_469aa  241 MPAVRGRRDFLTSSRMQSESKATMMCFLMVILFMVAWTPYTVSSFWSVLVEEIPLAAATY
```

```
Bb_op10_472aa  241 VPTASGRRDFVTSSRMQSETRATMMCFLMVILFMVAWTPYTVSSFWSTLVNEIPLWAATY

                       TM VII
                     ###*############
Bl_op10_469aa  301 PTMFAKSSCVFNPIIYAIAHKKFRSFLFQTCCPWLYRRVGLGEDNAYMEQRRRDRLRKQK
Bf_op10_469aa  301 PTMFAKSSCVFNPIIYAIAHKKFRTFLFQTCCPWLYRRVGLCKSNPYLEQQRKDRLRKQK
Bb_op10_472aa  301 PTMFAKSSCVFNPLIYAVAHKKFRSFLTQTCCPWLYRRVGLGKNNPYLDQQRQNRIRKQK

Bl_op10_469aa  361 GQIFCVQYTNGNVYIGSHGSRGCWSEDGSNNGENSGSCRYHRARASFSTLRVDNLTRQPQ
Bf_op10_469aa  361 GQIFCVQYTNGNVYIGPHGSRGCWSEDGSGDGGSSGRCHYHRASGSVSTLRVDNLARRPH
Bb_op10_472aa  361 GQIFCVQYTHGNVYIGAHGSRGCWSEDS---GRIRGTCRYHRARSSFSTLHVDNLARLSQ

Bl_op10_469aa  421 TGNT------LSNLHSKGRKYIVQAQVEPVPRTDNDTSSGEDSVHDLSCIDFAVI
Bf_op10_469aa  421 AGNA------PKTLHPKGRKYTVQAQVEPVPRTDNDSSSSEDSVHDLSCIDFTVL
Bb_op10_472aa  418 GGASSSTDSRLPSVHRKGRKYTVQAQIEPAPRQENSASSSDESVDDMSCIDFTVI
```

op11

op11							
B. lanceolatum	***B. floridae***				***B.belcheri***		**ScanProsite**[5]
Genomic scaffold in Assembly BraLan2[1]	JGI gene model (Putative allele or duplicate)[2]	NCBI gene model, Transcript and protein Accession numbers	Exon No and size[3]	Comments	Predicted gene model & scaffold in HapV2 assembly[4]	Comments	
Sc0000072 (1088219..1110938)<br><br>400aa<br>e1= 217<br>e2= 294<br>e3= 117<br>e4= 212<br>e5= 363	**256798*(110941 m)** scaffold_816<br>**+**<br>**84844 *** scaffold_135	**84844*** scaffold_36<br><br>XM_002608982<br>XP_002609028	401aa<br>e1= 217<br>e2= 294<br>e3= 117<br>e4= 212<br>e5= 366	First exons cannot be predicted in **84844** due to a string of Ns – **110941** contains the first 2 exons,**256798** only the 1st	BbT_037850F scaffold120‡<br><br>400aa<br>e1= 217<br>e2= 294<br>e3= 117<br>e4= 212<br>e5= 363	Repeats of genomic region here → prediction of 2 tandem copies. In v18h27.r3_ref_genome repeats were cleared out	GPCR + (79-332aa)<br>OPSIN + (316-332aa)

B. floridae

>Bf_op11_1206bp
ATGGACAACTCTACATGTTGTTTGAACGCTGCGGGCGGACCGCCGTGGACACCGAGACCGCCCGGCAGTC
GCCTTCGGGATGCCCACGACTGGGCCCCGAGCTGGAGTGACGAGGATCCGCTTCCAGGTGAAACCGACTT
CAACAGATACAACACTACCACGCACCAGCTGGACCCAGATGTACACATGGCTATCGCTATATACCTCACC
ATTGTTGGCGTTGTAGCTGTGGTCGGTAACGGCTTGGCGATCGGCGTATTCCTGAAAGAGAAGCAGTTCC
GAGGAAAGGAACACAACATCCTGCTGCTGAACCTGGCCGTGTCGGACCTGGCCGTCGCCGTGTTCGGCTA
CTCCTTCACGGTGATCGCCAGCTACGCCAAGCAGTGGCTGTTCGGCGAGGTGTGGTGCGTGCTGGACGGC
TTCATCTGCTTCACCTGTGCCATGAGCAGCATGAACACGCTATGTGTCATCAGCGTGTACCGGTACATCA
TCATCTGTAAACCACAGTATGCCTGCAGATTGACCCAGAGCTTCACCGTGCATGTTATCGTGGGTATCTG
GGCCTACGCCCTGGTCTGGACCGTACCGCCTCTGTTCGGATGGAGTTCGTACAGTATGCTGCCGTTTGGA
ACGTCCTGTGCCATAGACTGGTACGTGGAGAGTGTGTCTGATGCCCTGTACGTGTCCTTGTGTCTGCTGG
GGTGTTACGTTCTACACGTCACCGTCATGATCTTCTGCTACATCCGGGTCATTCGGCGGATGGATTCCAT
GCGATTCGCCGCCCTGGCCAGCGAAGAGGCGCGGCAAGTTGTGAAGAAGGACAAAAGGAAGAACATCATC
ATGTGCCTCTTCATGGTGGTGTCTTTCGTCGCCGTGTGGACACCGTACGCCGTCTCCTCGACCTGGGCAA
TATTTCAGCGGCACCTGCCGATCATGGCCCTTTACATCCCCACCATGTGCGCCAAGTCGTCCTGCATGCT
GAACCCCATCATCTACTCTGCATTCAACCAGCGATTCCGCAAGGCGGCGCTGCGCGTCGTAAAGGGGGCG
GCCGCTCAGGCACGGATCATGCCGGTTGGCTGGGGAACCCAGAATGGCACCGCCGCCATGATCAGGGCGA
GCACCTCCCGTCAGAACCGCTGCCAGGCCTACCTTCCCGAAGATATCAACATGGAGGCGATTGTCTACAA
AGATAACCTTGTGTAG

>Bf_op11_401aa
MDNSTCCLNAAGGPPWTPRPPGSRLRDAHDWAPSWSDEDPLPGETDFNRYNTTTHQLDPDVHMAIAIYLT
IVGVVAVVGNGLAIGVFLKEKQFRGKEHNILLLNLAVSDLAVAVFGYSFTVIASYAKQWLFGEVWCVLDG
FICFTCAMSSMNTLCVISVYRYIIICKPQYACRLTQSFTVHVIVGIWAYALVWTVPPLFGWSSYSMLPFG
TSCAIDWYVESVSDALYVSLCLLGCYVLHVTVMIFCYIRVIRRMDSMRFAALASEEARQVVKKDKRKNII
MCLFMVVSFVAVWTPYAVSSTWAIFQRHLPIMALYIPTMCAKSSCMLNPIIYSAFNQRFRKAALRVVKGA
AAQARIMPVGWGTQNGTAAMIRASTSRQNRCQAYLPEDINMEAIVYKDNLV

B. belcheri

>Bb_op11_1203bp
ATGGACAACTCTACATGCTGTTGGAGCACCTCAGGCGGGCCGCCCTGGACACCGGGACCGGCCGGCGGCC

TCCGTCGGGATGACCACGACTGGGCCCCGAACAGAAGCAACGATGAGCCGCTTTGGGCTGCAACCGACTT
CGACAGATACAACACCACCATGTACCAACTGGACCCAGACGTTCACATGGCTATTGCTATATACCTCACC
ATTGTTGGTATCGTAGCTGTGGTCGGTAACGGCTTGGCGATCAGCGTGTTTCTGAAAGAGAAGCAGTTCC
GCGGGAAGGAGCACAACATCCTGCTGCTGAACCTGGCCGTGTCCGACCTGGCCATCGCCGTGTTCGGCTA
CTCCTTCACGGTGATCGCCAGCTACGCCCGGCAGTGGCTGTTCGGCCACGTGTGGTGCGTGCTGGACGGC
TTCATCTGCTTCACCTGCGCCATGAGCAGCATGAACACGCTCTGTGTCATTAGCGTCTACCGATACATCA
TCATCTGCAAACCGCAGTATGCCTGTAGACTGACCCAGAGCTTCACCGTGCACGTTATCGTGGGGATCTG
GGTGTACGCCCTGGTCTGGACCGTGCCGCCGCTGTTTGGGTGGAGTTCGTACAGCATGCTGCCGTTCGGA
ACGTCCTGCGCCATAGACTGGTACGTGTCGAGCGTGTCTGACGCCCTGTACGTCTCCCTGTGTCTGCTGG
GTTGTTACGTCCTACACATCACCGTCATGGCGTTCTGCTACATCCGGGTCATCCGGCGGATGGATTCCAT
GCGATTCGCCGCCCTGGCCAGCGAAGAGGCGCGACAAGTCGTGAAGAAGGACAAAAGGAAGAACATCATC
ATGTGCCTCTCCATGGTGGTGGCCTTCCTCGCCGTGTGGACGCCGTACGCCGTCTCCTCGACCTGGGCCA
TATTCCAGCGCCACCTGCCGATCATGGCCCTTTTCATCCCCACCATGTGCGCCAAGTCGTCCTGCATGCT
CAACCCCATCATCTACACTGCATTCAACCAACGGTTCCGCAAGGCGGCGCTGCGCGTCGTGAAGGGGGCG
GCTGCTCACGCACGGATCGTGCCCGTGGGCTGGGGACACCGGAGCGACGCCGCCGTGAGCAGGGCCAACA
CCTCGCGCGAGAATCGCTTTCAGGCGTACCTTCCCGAGGATATCAGCATGGCGACCATTGGTTACAGAGA
TAACCTGGTGTAA

>Bb_op11_400aa
MDNSTCCWSTSGGPPWTPGPAGGLRRDDHDWAPNRSNDEPLWAATDFDRYNTTMYQLDPDVHMAIAIYLT
IVGIVAVVGNGLAISVFLKEKQFRGKEHNILLLNLAVSDLAIAVFGYSFTVIASYARQWLFGHVWCVLDG
FICFTCAMSSMNTLCVISVYRYIIICKPQYACRLTQSFTVHVIVGIWVYALVWTVPPLFGWSSYSMLPFG
TSCAIDWYVSSVSDALYVSLCLLGCYVLHITVMAFCYIRVIRRMDSMRFAALASEEARQVVKKDKRKNII
MCLSMVVAFLAVWTPYAVSSTWAIFQRHLPIMALFIPTMCAKSSCMLNPIIYTAFNQRFRKAALRVVKGA
AAHARIVPVGWGHRSDAAVSRANTSRENRFQAYLPEDISMATIGYRDNLV

```
Bl_op11_400aa     1 MDNSTCCLNSTGGPPWTPRPTGSLHLDDFDWVPTRSDDDPLRGETDFNRYNTTMYQLDPD
Bf_op11_401aa     1 MDNSTCCLNAAGGPPWTPRPPGSRLRDAHDWAPSWSDEDPLPGETDFNRYNTTTHQLDPD
Bb_op11_400aa     1 MDNSTCCWSTSGGPPWTPGPAGGLRRDDHDWAPNRSNDEPLWAATDFDRYNTTMYQLDPD

                      TM I                             TM II
                  ####################              ####################
Bl_op11_400aa    61 VHMAIAIYLTIVGVVAVIGNGLAIGVFLKEKQFRGKEHNILLLNLAVSDLAIAVFGYSFT
Bf_op11_401aa    61 VHMAIAIYLTIVGVVAVVGNGLAIGVFLKEKQFRGKEHNILLLNLAVSDLAVAVFGYSFT
Bb_op11_400aa    61 VHMAIAIYLTIVGIVAVVGNGLAISVFLKEKQFRGKEHNILLLNLAVSDLAIAVFGYSFT

                                    TM III
         #                ######################                     #
Bl_op11_400aa   121 AIASYARQWLFGPVWCVLDGFICFTCAMSSMNTLCVISVYRYIIICKPQYACRLTQSFTV
Bf_op11_401aa   121 VIASYAKQWLFGEVWCVLDGFICFTCAMSSMNTLCVISVYRYIIICKPQYACRLTQSFTV
Bb_op11_400aa   121 VIASYARQWLFGHVWCVLDGFICFTCAMSSMNTLCVISVYRYIIICKPQYACRLTQSFTV

                         TM IV                                         TM V
              ###################                         #############
Bl_op11_400aa   181HVIVGIWAYALVWTVPPLFGWSSYTMQPSGTSCAIDWYVENVSDALYVSMCLLGCYVLHV
Bf_op11_401aa   181 HVIVGIWAYALVWTVPPLFGWSSYSMLPFGTSCAIDWYVESVSDALYVSLCLLGCYVLHV
Bb_op11_400aa   181 HVIVGIWVYALVWTVPPLFGWSSYSMLPFGTSCAIDWYVSSVSDALYVSLCLLGCYVLHI

                                                 TM VI
       ########                              #####################
Bl_op11_400aa   241TVMVFCYIRVIRRMDSMQFAALASEEARQVVKKDKRKNIIMCLFMVVAFVAVWTPYAVSS
Bf_op11_401aa   241 TVMIFCYIRVIRRMDSMRFAALASEEARQVVKKDKRKNIIMCLFMVVSFVAVWTPYAVSS
Bb_op11_400aa   241 TVMAFCYIRVIRRMDSMRFAALASEEARQVVKKDKRKNIIMCLSMVVAFLAVWTPYAVSS

                                     TM VII
                        ########*############
Bl_op11_400aa   301TWAIFQRHLPVMALYIPTMCAKSSCMLNPIIYTAFNQRFRKAALRVVKGAAAHVQIVPVG
Bf_op11_401aa   301 TWAIFQRHLPIMALYIPTMCAKSSCMLNPIIYSAFNQRFRKAALRVVKGAAAQARIMPVG
Bb_op11_400aa   301 TWAIFQRHLPIMALFIPTMCAKSSCMLNPIIYTAFNQRFRKAALRVVKGAAAHARIVPVG
```

```
Bl_op11_400aa   361 WGSRND-AVMARASTSRQNRCEAYLPEDINMATIVFRDNLV
Bf_op11_401aa   361 WGTQNGTAAMIRASTSRQNRCQAYLPEDINMEAIVYKDNLV
Bb_op11_400aa   361 WGHRSD-AAVSRANTSRENRFQAYLPEDISMATIGYRDNLV
```

op12a+op12b+Amphiop1

op12a							
B. lanceolatum	***B. floridae***				***B.belcheri***		**ScanProsite[5]**
Genomic scaffold in Assembly BraLan2[1]	JGI gene model (Putative allele or duplicate)[2]	NCBI gene model, Transcript and protein Accession numbers	Exon No and size[3]	Comments	Predicted gene model & scaffold in HapV2 assembly[4]	Comments	
Sc0000154 (194247..201469)<br><br>386aa<br>e1= 172<br>e2= 153<br>e3= 141<br>e4= 117<br>e5= 212<br>e6= 366	**91094**<br>scaffold_205	**91094**<br>scaffold_36<br><br>XM_002609070<br>XP_002609116	381aa<br>e1= 181<br>e2= 153<br>e3= 141<br>e4= 117<br>e5= 212<br>e6= 342		025960/220050*Sc0000271 (scaffold123‡)<br><br>385aa<br>e1= 181<br>e2= 153<br>e3= 141<br>e4= 117<br>e5= 212<br>e6= 354		GPCR + (67-321aa)<br>OPSIN + (305-321aa)
**op12b**							
Not present	Not present				025970/220050*<br>Sc0000271<br>(scaffold123‡)<br><br>344aa<br>e1= 181<br>e2= 153<br>e3= 141<br>e4= 117<br>e5= 212<br>e6= 231		GPCR + (67-321aa)<br>OPSIN + (305-321aa)

B. floridae

```
>Bf_op12a_1146bp
ATGAACGCCTCTCCCAGCTCCTGGCTGCCGAGCGGAGAGCTGTTCACGGACAGCCCGGAGAACTCCAGCG
AGTGGCCGTGGACGGACGGACCGACTGACACGGCATGGCACCATCACCAGACGGTAGACCCTGTAACATA
TGGGGGCTACCTCGCAAGTGCAGTCTACCTTACAATAACAGGGCTGATAGCCTTCGTTGGAAACATTTTC
GCCATCATAGTGTTCTTGACGGAAAAGGAGTTCCGCAAAAAAGAGCACAACAGTTTTGCTCTGAATCTGG
CCATTGCTGACTTGAGCGTCTGCGTCTTCGCATATCCTTCGTCTACTATATCAGGGTACGCAGGAGAATG
GATGCTGGGAGACGTTGGCTGTACCATCTATGGCTTCCTCTGCTTCACCTTTTCCCTGACCAGCATGGTC
ACCCTGTGCGCTATCAGCGTCTACCGCTACATCGTCATCTGTAAACCGCAGTATGCCCATCTGCTGACCC
ACCGCAGGACCAACTATGTGATCCTGGGGATCTGGCTGTACGCCCTGGTGTTCTCTGTCCCTCCGCTGTT
TGGAGTCAACCGCTACACCTACGAACCAATCAAAGTCACCTGCTCCTTGGACTGGAACGTGCAGCATGTC
GGAGAGACAATCTACACTGCGGCGGTCATCATCATAGTCTACGTCCTGAACGTCTCGATCATGTGCTTCT
GCTACTTCAACATCATCTTCAAATCGGCTAACCTCAAGTTTGCGGCTCTGGCCAGTGAGAAAACTAGGAC
GGCTGCAAAAAAAGACATCTGGAAGACCTCAATGATGTGCCTGGCTATGGTGGTCTCCTTCCTCATCGCC
TGGACCCCGTACGCCGTGTCCTCTACCTGGGACATCCTGACTGAGGAAGATCTACCGATCATCGCCACCA
TCCTGCCCACCATGTTCGCCAAGTCGTCCTGCATGATGAACCCCATCATCTACTCCTGCTGTAACGGGAA
```

ATTCCGACAGGCGGCGCTCAAGACCTTCAGCAAGGTCGGCAGTTCCAACAAGCAGAACGGTCAAGCACAA
GTGGAACCGAGGGACCCGGGTTTCGCTGTTGAACCCGCAGGTCATCAGGCCTTCCAGATGAGGGTTCTTC
CTTCTAGTAGCGCTATGACTTTGTAG

>Bf_op12a_381aa
MNASPSSWLPSGELFTDSPENSSEWPWTDGPTDTAWHHHQTVDPVTYGGYLASAVYLTITGLIAFVGNIF
AIIVFLTEKEFRKKEHNSFALNLAIADLSVCVFAYPSSTISGYAGEWMLGDVGCTIYGFLCFTFSLTSMV
TLCAISVYRYIVICKPQYAHLLTHRRTNYVILGIWLYALVFSVPPLFGVNRYTYEPIKVTCSLDWNVQHV
GETIYTAAVIIIVYVLNVSIMCFCYFNIIFKSANLKFAALASEKTRTAAKKDIWKTSMMCLAMVVSFLIA
WTPYAVSSTWDILTEEDLPIIATILPTMFAKSSCMMNPIIYSCCNGKFRQAALKTFSKVGSSNKQNGQAQ
VEPRDPGFAVEPAGHQAFQMRVLPSSSAMTL

B. belcheri

>Bb_op12a_1158bp
ATGGACACCTCTCCCAGCTCCTGGCTGCCCGGCGGAGAGTTCTTCACGGACAGCCCGGAGAACTCCAGCG
AGTGGCCCTGGACGGACGGACCGACCGACACGGGATGGCGCCATCACCAGTCGGCAGACCCCGTGGGCTA
TGAAGGCTACCTCGCCAGTGCAATCTACCTTACCCTCACAGGGTTGATAGCCTTACCCGGGAACGTCATC
GCCATCACCGTGTTCCTGACGGAAAAGGAGTTCCGTAAGAAACAGCAGAACAGTTTTGTCCTGAATCTGG
CCATAGCTGACTTCAGCGTCTGCGTCTTCGCATATCCCTCGTCTACCATAGCAGGGTTCGCAGGAGGATG
GGTGCTCGGAGATGTTGGCTGTACGATCTATGGGTTCCTCTGCTTCACTTTTTCCCTGACCAGCATGGTC
ACCCTGTGTGCCATCAGCGTCTACCGCTACATCGTCATCTGTAAACCACAGTATGCCCATCTGCTGACCC
ATCGCAGGACCAACTTTGTGATCATCGGGATCTGGCTGTACGCGCTGGTGTTCACGGTCCCTCCGCTAGT
CGGCGTCAACCGCTACACTTACGAACCAATGCACATCACCTGCTCCTTGGACTGGAACGTGCAGTACCCC
GGGGAGACGGCCTACCTCGCAGCGGTTCTCGTCATCGTCTTCGTCCTGCAGGTCCTGATCATGTGTTTCT
GCTACTTCAACATCATCGTCAAGTCGGCCAACCTCAAGTTTGCGGCTCTGGCCAATGAGAGAACCAAGAA
GGCCGCAAAGAAAGACACCTGGAAGACATCAATGATGTGCCTGACCATGGTGGTGTCCTTCCTGATCGCC
TGGACCCCGTATGCCGTGTCCTCTACCTGGGACATCCTGTCTGAGGAAGATCTGCCCATTATCGCCACCA
TCCTGCCCAGCATGTTCGCCAAGTCGTCCTGCATGATGAACCCAATCATCTACTCTTGCTGTAGCAATAA
GTTCCGCCAGGCGGCTGCAAAGTCCTTCCGCAGGCTCGGCTGTATGCGTAAGCAGAGCGTTCCCCCGTCT
CCAGCAGAACTGCGGAACACAGTTCTGGAGTTCACAGCCGAACCTGCTGGTCAGGCCATTCCCATGAGCG
CTCTCCCTTCTAGTAGCGCTAAGTGTACTTCTTTGTAA

>Bb_op12a_385aa
MDTSPSSWLPGGEFFTDSPENSSEWPWTDGPTDTGWRHHQSADPVGYEGYLASAIYLTLTGLIALPGNVI
AITVFLTEKEFRKKQQNSFVLNLAIADFSVCVFAYPSSTIAGFAGGWVLGDVGCTIYGFLCFTFSLTSMV
TLCAISVYRYIVICKPQYAHLLTHRRTNFVIIGIWLYALVFTVPPLVGVNRYTYEPMHITCSLDWNVQYP
GETAYLAAVLVIVFVLQVLIMCFCYFNIIVKSANLKFAALANERTKKAAKKDTWKTSMMCLTMVVSFLIA
WTPYAVSSTWDILSEEDLPIIATILPSMFAKSSCMMNPIIYSCCSNKFRQAAAKSFRRLGCMRKQSVPPS
PAELRNTVLEFTAEPAGQAIPMSALPSSSAKCTSL

B. belcheri

>Bb_op12b_1035bp
ATGGACACCTCTCCCAGCTCCTGGCTGCCCGGCGGGGAGTTCTTCACGGACAACCCGGAGAACTCCAGCG
AGTGGCCCTGGACGGACGGACCGACCGACACGGGATGGCGCCATCACCAGTCGGCAGACCCCGTGGGCTA
TGAAGGCTACCTCGCCAGTGCAATCTACCTTACCCTCACAGGGTTGATAGCCTTACCCGGGAACGCCGTC
GCCATCACGGTGTTCCTGACGGAAAAGGACTTCCGTAAGAAACAGCAGAACAGTTTTGTCCTGAATCTGG
CCATAGCTGACTTCAGCGTCTGCGTCTTCGCATATCCCTTGTCTGCTACAGCAGGGTTCGCAGGAGGATG
GGTGCTCGGAGATGTTGGCTGTACGATCTATGGGTTCCTCTGCTTCACTTTTTCCCTGGTCAGCATGGTC
ACCCTGTGTGCCATCAGCGTCTACCGCTACATCGTCATCTGTAAACCACAGTATGCGCATCTGCTGACCC
ATCGCAGGACCAACTACGTCATCATCGGGATCTGGCTGTTCTCTCTGGTGTTCACGGTCCCTCCGCTAGT
CGGACTTAACCGCTACACTTACGATCCAATGGGGATCATCTGCTCCTTGGACTGGAACGTGCAGCACCCC
ACAGAGACGGCCTACGTCGTGGCGGTTCTTGTTATCCTCTATATCCTGCAGGTCCTGATCATGTGCTTCT
GCTACTTCAACATCATCGTCAAGTCGGCCAACTTGAAGTTCGCGGCTCTGGCCAATGAAGAAACTAAAAA
GGCTGCAAAGAAAGACACCTCGAAGACCTCACTGATGTGCCTGACCATGGTGGTGTCCTTCCTGATCGCC
TGGACCCCGTATGCCGTGTCCTGTACCTGGGACATCCTGTCTGAGGAAGATCTGCCCATCATCGCCACCA
TCCTGCCCAGCTTGTTCGCCAAGTCGTCCTGCATGATGAACCCCGTCATCTACGCCTGCTGTAGCAATAA
GTTCCGCCAGGCGGCAGCTAAGTCCTTCCGCAGGCTTGGCTTTATGCGCAGGTAA

>Bb_op12b_344aa
MDTSPSSWLPGGEFFTDNPENSSEWPWTDGPTDTGWRHHQSADPVGYEGYLASAIYLTLTGLIALPGNAV
AITVFLTEKDFRKKQQNSFVLNLAIADFSVCVFAYPLSATAGFAGGWVLGDVGCTIYGFLCFTFSLVSMV

```
TLCAISVYRYIVICKPQYAHLLTHRRTNYVIIGIWLFSLVFTVPPLVGLNRYTYDPMGIICSLDWNVQHP
TETAYVVAVLVILYILQVLIMCFCYFNIIVKSANLKFAALANEETKKAAKKDTSKTSLMCLTMVVSFLIA
WTPYAVSCTWDILSEEDLPIIATILPSLFAKSSCMMNPVIYACCSNKFRQAAAKSFRRLGFMRR
```

```
                                    TM
     ########
Bl_op12a_386aa       1 MSASPSAWLSSRELFTDSPENSSEWPWTDGPTDTTLRHYQF---VTYEGYLASAVYLTLT
Bf_op12a_381aa       1 MNASPSSWLPSGELFTDSPENSSEWPWTDGPTDTAWHHHQTVDPVTYGGYLASAVYLTIT
Bb_op12a_385aa       1 MDTSPSSWLPGGEFFTDSPENSSEWPWTDGPTDTGWRHHQSADPVGYEGYLASAIYLTLT
Amphiop1_BAC76019   1 MNASPSSWLSSGEFFTDSPENSSEWPWTDGPTDTTWRHHQSVDSVSYEGYLASAIYITLT
Bb_op12b_344aa       1 MDTSPSSWLPGGEFFTDNPENSSEWPWTDGPTDTGWRHHQSADPVGYEGYLASAIYLTLT

             I                 TM II
   ##############        ######################
Bl_op12a_386aa      58 GTIGFLGNVIAITVFLTRKEFRQKEHNSLVLNLAIADFSICVFAYPSSTISGYAGRWLLG
Bf_op12a_381aa      61 GLIAFVGNIFAIIVFLTEKEFRKKEHNSFALNLAIADLSVCVFAYPSSTISGYAGEWMLG
Bb_op12a_385aa      61 GLIALPGNVIAITVFLTEKEFRKKQQNSFVLNLAIADFSVCVFAYPSSTIAGFAGGWVLG
Amphiop1_BAC76019  61 GLIAFFGNVITITVFLTEKEFRKKQQNGFVLNLAIADLSVCVFAYPSSAIAGYAGRWVLG
Bb_op12b_344aa      61 GLIALPGNAVAITVFLTEKDFRKKQQNSFVLNLAIADFSVCVFAYPLSATAGFAGGWVLG

  TM III                                   TM
 ######################                  ###############
Bl_op12a_386aa      118 DVGCTIYGFLCFTFSMVSMVTLCAVSVYRYIVICKPQFAYLLTHRRTMYVIPGIWLYALL
Bf_op12a_381aa      121 DVGCTIYGFLCFTFSLTSMVTLCAISVYRYIVICKPQYAHLLTHRRTNYVILGIWLYALV
Bb_op12a_385aa      121 DVGCTIYGFLCFTFSLTSMVTLCAISVYRYIVICKPQYAHLLTHRRTNFVIIGIWLYALV
Amphiop1_BAC76019  121 DVGCTIYGFLCFTFALVSMVTLCVISIYRYILICKPQYAHLLTHRRTVYVIIGTWLYALV
Bb_op12b_344aa      121 DVGCTIYGFLCFTFSLVSMVTLCAISVYRYIVICKPQYAHLLTHRRTNYVIIGIWLFSLV

 IV                                  TM V
  #######                          ######################
Bl_op12a_386aa      178 FTIPPLVGVNRYTFEPIRVSCSLDWKVQYPGEKVYIAAVIIFVYVLNVSTMCFCYFNIIY
Bf_op12a_381aa      181 FSVPPLFGVNRYTYEPIKVTCSLDWNVQHVGETIYTAAVIIIVYVLNVSIMCFCYFNIIF
Bb_op12a_385aa      181 FTVPPLVGVNRYTYEPMHITCSLDWNVQYPGETAYLAAVLVIVFVLQVLIMCFCYFNIIV
Amphiop1_BAC76019  181 FTVPPLVGVKRYTYEPMQITCSLDWNVQHPGEKAYIAAVLVIVYVLQVLIMCFCYFNIIF
Bb_op12b_344aa      181 FTVPPLVGLNRYTYDPMGIICSLDWNVQHPTETAYVVAVLVILYILQVLIMCFCYFNIIV

                                   TM VI
  ######################
Bl_op12a_386aa      238 KSANLKFAALASEKTRKAAKKDIWRTSMMCLTMVVSFLIAWTPYAVSCTWDILSDEDLPI
Bf_op12a_381aa      241 KSANLKFAALASEKTRTAAKKDIWKTSMMCLAMVVSFLIAWTPYAVSSTWDILTEEDLPI
Bb_op12a_385aa      241 KSANLKFAALANERTKKAAKKDTWKTSMMCLTMVVSFLIAWTPYAVSSTWDILSEEDLPI
Amphiop1_BAC76019  241 KSANLKFAALASEKTKMAAKKDTWKTSVMCLTMVVSFLIAWTPYAVSSTWDILSAEDLPI
Bb_op12b_344aa      241 KSANLKFAALANEETKKAAKKDTSKTSLMCLTMVVSFLIAWTPYAVSCTWDILSEEDLPI

                                   TM VII
########*#############
Bl_op12a_386aa      298 LATILPTMFAKASCMMNPIIYACCSKAFRQAAATSFRRLGCWSRQNAPTQVAPSDAVFTV
Bf_op12a_381aa      301 IATILPTMFAKSSCMMNPIIYSCCNGKFRQAALKTFSKVGSSNKQNGQAQV--------E
Bb_op12a_385aa      301 IATILPSMFAKSSCMMNPIIYSCCSNKFRQAAAKSFRRLGCMRKQSVPPSPAE-----LR
Amphiop1_BAC76019  301 IATILPSLFAKSSCMMNPIIYACCNTKFRQAAVKSFRKLCGMCKQKVPLSTPQVVLAMQR
Bb_op12b_344aa      301 IATILPSLFAKSSCMMNPVIYACCSNKFRQAAAKSFRRLGFMRR----------------


Bl_op12a_386aa      358 QGSPAVRGSPTG-QAFEMRVLPSTSAVTSV-
Bf_op12a_381aa      353 PRDPGFAVEPAGHQAFQMRVLPSSSAMTL--
Bb_op12a_385aa      356 NTVLEFTAEPAG-QAIPMSALPSSSAKCTSL
Amphiop1_BAC76019  361 NTEFTSTVEPTG-QAFPMRVLPSISATHTAL
Bb_op12b_344aa          -------------------------------
```

op13a+op13b

op13a							
B. lanceolatum	***B. floridae***				***B.belcheri***		**ScanProsite[5]**
Genomic scaffold in Assembly BraLan2[1]	JGI gene model (Putative allele or duplicate)[2]	NCBI gene model, Transcript and protein Accession numbers	Exon No and size[3]	Comments	Predicted gene model & scaffold in HapV2 assembly[4]	Comments	
Sc0000154<br>(175052..184700)<br><br>386aa<br>e1= 172<br>e2= 153<br>e3= 141<br>e4= 117<br>e5= 212<br>e6= 366	**91095***<br>**(91106[d])**<br>scaffold_205	**91095***(499aa)<br>scaffold_36<br><br>XM_002609071<br>XP_002609117	391aa<br>e1= 181<br>e2= 153<br>e3= 141<br>e4= 117<br>e5= 212<br>e6= 372		025950/220040F*<br>Sc0000271<br>(scaffold123‡)<br><br>388aa<br>e1= 175<br>e2= 153<br>e3= 141<br>e4= 117<br>e5= 212<br>e6= 369		GPCR +<br>(67-321aa)<br>OPSIN +<br>(305-321aa)
**op13b**							
Sc0000040<br>(970123..981814)<br><br>387aa<br>e1= 175<br>e2= 153<br>e3= 141<br>e4= 117<br>e5= 212<br>e6= 366	Not present				Not present		GPCR +<br>(65-319aa)<br>OPSIN +<br>(303-319aa)

B. floridae

```
>Bf_op13a_1176bp
ATGAACGCCTCTCCCAGCTCCTGGCTGCCGAGCAGAGAGCTGTTCACGGACAGCCCGGAGAACTCCAGCG
AGTGGCCGTGGACGGACGGACCGACACAAGAGTCCTGTTCCGACGATGAGTCTGTCGACCCTATCGGCTA
CCATGCCTACCTTGCAGTCGCGATCTATCTCACCCTTCTTGGGATAGTAGCAGTTGGTGGAAATGTCATG
GCCATCATCGTATTCGTGACAGAAAAAGAGTTCCGTAAGAAAGAGCACAACAGTCTTCTTCTGAACATGG
CCTTAGCTGACTTAGGCGTCAGCATCTTCGGGTATCCCGCCTCCACTGTGTCAGGGTACGCAGGAAGATG
GGTGCTGGGAGAAGTGTGGTGTGTGATATATGCGTTTGTCTGCTACACCTTCTCCATGGTCACCATGAAC
ACCCTGTGTGCCATCAGCATCTATCGCTACATCGTCATGTGTACGCCACAGTATGCCCATCTGCTGACCC
ACCGCAGGACCATGTATGTGATCCTGGTGACCTGGCTGTACGCGCTGGTGTTCACGGTGCCTCCACTGGT
CGGAGTCAACTACTACACCTTCGAACCGATCCGAATCATCTGCTCCTTGAACTGGAACGTGCGGTATCCC
GGAGAGATGGCCTACACCGTACTGACCATCTTCTTCGGCTATGTCGTCTATGTCGTTATCATGGGGTACT
GCTACTGCAAGATCTACTTCAAGTCGAAGAATCTCAAGTTTGGCGCCCTGGCCAGTGACAAGGCCAAGAA
GGCTGTGAAGAAGGATATCCTAAAGGCTTCATTGATGTGCATGACTATGGTGGTGTCCTTCCTCATCGCC
TGGACCCCGTACGCCGTGTCCTCTACCTGGGACATCCTGACTGAGGAAGATCTACCGATCATCGCCACCA
TCCTGCCCAGCCTGTTCGCCAAGTCGTCCTGCATGATGAACCCCATCATCTACACCTGCTGTAACGGCAA
ATACCGACAGGCGGCGCTCAAGTCCTTCCGCAGGCTTGGTTGCTTCAACAAGCAGGTGAATCCGTTGAAT
ACGGAACAGCCACAGGCTGTCCGCCGGAGAAGGTGCTCTGCAGTAGGGCCTGCTGATCAGGGCATGGCCA
TGAACGCCATTCCACCTAGCAGGGCCACGAACGACAGGCAGTCGAGTTCTTGTTAG
```

```
>Bf_op13a_391aa
MNASPSSWLPSRELFTDSPENSSEWPWTDGPTQESCSDDESVDPIGYHAYLAVAIYLTLLGIVAVGGNVM
AIIVFVTEKEFRKKEHNSLLLNMALADLGVSIFGYPASTVSGYAGRWVLGEVWCVIYAFVCYTFSMVTMN
TLCAISIYRYIVMCTPQYAHLLTHRRTMYVILVTWLYALVFTVPPLVGVNYYTFEPIRIICSLNWNVRYP
GEMAYTVLTIFFGYVVYVVIMGYCYCKIYFKSKNLKFGALASDKAKKAVKKDILKASLMCMTMVVSFLIA
WTPYAVSSTWDILTEEDLPIIATILPSLFAKSSCMMNPIIYTCCNGKYRQAALKSFRRLGCFNKQVNPLN
TEQPQAVRRRRCSAVGPADQGMAMNAIPPSRATNDRQSSSC
```

B. belcheri

```
>Bb_op13a_1167bp
ATGGACGCCTCTCCCAGCTCCTGGCTGCCCGGCGGGGAGTTCTTCACGGACAGCCCGGAGAACTCCAGCG
AGTGGCCCGGGACGGACGGACCGACCCAAGAGATTTGTTCCGAGGACGAAGTTGTCATAGGCTACCATGC
CTACCTTGCTGTCGCGATCTATCTCACCCTTCTTGGAATAGTATCTGTTGCCGGGAATGCCATGTCCATC
ATCGTATTCGTGACAGAGAAAGAGTTCCGTAAAAAAGAGCACAACAGTCTTCTCTTGAACATGGCCATAG
CTGACTTGGGCGTCAGCATCTTTGGATATCCCGCCTCCACCGTGTCAGGGTTCGCAGGAAGATGGGTGAT
GGGGGACGGGTATTGTCCTGCATATGCGTTCATCTGCTACACCTTCTCTATGGTCACCATGATCACCCTG
GGTGCCATCAGCGTCTACAGATACATCGTCATCTGTAAACCACAGTATGCCCATCTGCTGACCCATCGCA
GGACCATGTATGTGATCCTCGGGATCTGGCTGTACGCCCTGACGTTCACGGTCCCTCCGCTAGTCGGCGT
CAACTACTACACCTATGAACCGATCCGAATCATCTGCTCCCTGAACTGGAACGTGCAGTACCCGGGAGAG
ATCGCCTACACCGCGTACTGCATCATCTTCGGCTACGTCATCAAGGTCGTCATCATGGCGTACTGCTACT
TCAACATCCTCTTCAAGTCGATGAACCTGAAGTTTGGCGCTCTGGCCAGCGAGAAGGCCAAGAAGGCTGT
GAAGAAAGACATCATGAAGGCCTCGCTGATGTGCCTGACCATGGTGGTGTCCTTCCTGATCGCCTGGACC
CCGTATGCCGTGTCCTGTACCTGGGACATCCTGTCTGAGGAAGATCTGCCCATCATCGCCACAATCCTGC
CCAGCTTGTTCGCCAAGTCGTCCTGCATGATGAACCCCGTCATCTACGCCTGCTGTAACGGCAAGTTCCG
CCAGGCGGCTGCAAAGTCCTTCCGCAGGCTCGGCTGTATGAATAACCAGGTAAATCCTCTGAATACCGTG
CCAGTGGTGCGCGGGAACAGGTGCCCTGCGGCAGGGCCTGCCGAGGAGGGCATGGCCATGAAGTCCGTTC
CACCCAGTGCTGCGGCCACGGTCGACAGGCAATCGACTTCTTGTTAG
```

```
>Bb_op13a_388aa
MDASPSSWLPGGEFFTDSPENSSEWPGTDGPTQEICSEDEVVIGYHAYLAVAIYLTLLGIVSVAGNAMSI
IVFVTEKEFRKKEHNSLLLNMAIADLGVSIFGYPASTVSGFAGRWVMGDGYCPAYAFICYTFSMVTMITL
GAISVYRYIVICKPQYAHLLTHRRTMYVILGIWLYALTFTVPPLVGVNYYTYEPIRIICSLNWNVQYPGE
IAYTAYCIIFGYVIKVVIMAYCYFNILFKSMNLKFGALASEKAKKAVKKDIMKASLMCLTMVVSFLIAWT
PYAVSCTWDILSEEDLPIIATILPSLFAKSSCMMNPVIYACCNGKFRQAAAKSFRRLGCMNNQVNPLNTV
PVVRGNRCPAAGPAEEGMAMKSVPPSAAATVDRQSTSC
```

```
                                                                         ######
Bl_op13a_386aa    1 MSASPSAWLSSGELFTDSPENSSEWPWTDGPTQETCSD-DD--HFGYESYLAIAIYLTLL
Bl_op13b_387aa    1 MSASPSAWLSSGELVTDSPENTSEWPWTDGPTQETCADDDD--HFGYESYLAVAIYLTLL
Bf_op13a_391aa    1 MNASPSSWLPSRELFTDSPENSSEWPWTDGPTQESCSDDESVDPIGYHAYLAVAIYLTLL
Bb_op13a_388aa    1 MDASPSSWLPGGEFFTDSPENSSEWPGTDGPTQEICSEDEV--VIGYHAYLAVAIYLTLL

  TM I                              TM II
 ################          ######################
Bl_op13a_386aa   58 GLMAIGGNSIAIITFVTEKEFRKKEHNILLLNMAIADLGVSIFGYPSSTVSGYAGRWLLG
Bl_op13b_387aa   59 GLMAIGGNSIAIITFVRKKEFRNKEHNSLLLNMAIADLGVSIFAYPSTTVSGYAGKWMLG
Bf_op13a_391aa   61 GIVAVGGNVMAIIVFVTEKEFRKKEHNSLLLNMALADLGVSIFGYPASTVSGYAGRWVLG
Bb_op13a_388aa   59 GIVSVAGNAMSIIVFVTEKEFRKKEHNSLLLNMAIADLGVSIFGYPASTVSGFAGRWVMG

    TM III                                  TM
 ######################                 ##############
Bl_op13a_386aa  118DVGCTMYAFLCFTFSLVSMFTLCAVSVYRYIVICKPQYAYMLNHRRTMYVTLGTWMYALL
Bl_op13b_387aa  119 DVGCTIFGFLCFTFSLVTEGTLCAISVYRYIVICKPQHAYLLTHRRTMYVILGTWVYALL
Bf_op13a_391aa  121 EVWCVIYAFVCYTFSMVTMNTLCAISIYRYIVMCTPQYAHLLTHRRTMYVILVTWLYALV
Bb_op13a_388aa  119 DGYCPAYAFICYTFSMVTMITLGAISVYRYIVICKPQYAHLLTHRRTMYVILGIWLYALT

IV                                      TM V
 #######                            ##################
Bl_op13a_386aa  178FSIPPLVGVSFYTYEPIRIICSLNWKLQYPGEMAYTAVTITFCYVANVWIMGYCYFNIFS
Bl_op13b_387aa  179 FTIPPLVGVSYYTYEPIRLICSLNWKLQYPGEIAYTALTIVFCYIANVWIMGYCYFKIFS
```

```
Bf_op13a_391aa  181 FTVPPLVGVNYYTFEPIRIICSLNWNVRYPGEMAYTVLTIFFGYVVYVVIMGYCYCKIYF
Bb_op13a_388aa  179 FTVPPLVGVNYYTYEPIRIICSLNWNVQYPGEIAYTAYCIIFGYVIKVVIMAYCYFNILF

                                                TM VI
     ######################
Bl_op13a_386aa  238KSTNLKFGALASEKAKKAVKRDILKAAMMCLTMVVSFLIAWTPYAVSSTWNVLVYGDLPV
Bl_op13b_387aa  239 KSTNLKFGALASEKAKKAVKSDILKAARMCLAMVVSYLFVWTPYAVSSTWNVFVDGDLPV
Bf_op13a_391aa  241 KSKNLKFGALASDKAKKAVKKDILKASLMCMTMVVSFLIAWTPYAVSSTWDILTEEDLPI
Bb_op13a_388aa  239 KSMNLKFGALASEKAKKAVKKDIMKASLMCLTMVVSFLIAWTPYAVSCTWDILSEEDLPI

                          TM VII
  ########*#############
Bl_op13a_386aa  298 LATILPSLFSKLSCMMNPIIYTCCNSKFRQAVSKSLRRLGCLHKQVNPLDTA--QMVRRK
Bl_op13b_387aa  299 LATVLPSLFAKTSCMLNPIIYTCCNSKFRQAVSKSLRRRGCLNKQVNPLDTA--QMVRRK
Bf_op13a_391aa  301 IATILPSLFAKSSCMMNPIIYTCCNGKYRQAALKSFRRLGCFNKQVNPLNTEQPQAVRRR
Bb_op13a_388aa  299 IATILPSLFAKSSCMMNPVIYACCNGKFRQAAAKSFRRLGCMNNQVNPLNTV--PVVRGN

Bl_op13a_386aa  356 RRSDVEPAAGGMAMNAVPPSMA-TGDRQSSSC
Bl_op13b_387aa  357 RRSDVELAAEGMAMKAVPPSMA-TGDGQSSSC
Bf_op13a_391aa  361 RCSAVGPADQGMAMNAIPPSRA-TNDRQSSSC
Bb_op13a_388aa  357 RCPAAGPAEEGMAMKSVPPSAAATVDRQSTSC
```

op14 + Amphiop3

op14							
B. lanceolatum	***B. floridae***				***B.belcheri***		**ScanProsite**[5]
Genomic scaffold in Assembly BraLan2[1]	JGI gene model (Putative allele or duplicate)[2]	NCBI gene model, Transcript and protein Accession numbers	Exon No and size[3]	Comments	Predicted gene model & scaffold in HapV2 assembly[4]	Comments	
Sc0000015<br>(325502..342752)<br><br>366aa<br>e1= 136<br>e2= 158<br>e3= 133<br>e4= 117<br>e5= 119<br>e6= 105<br>e7= 266<br>e8= 67	**90832***<br>scaffold_202<br>(108075[a]<br>Scaffold_607)	**90832***<br>scaffold_70<br>(360aa)<br><br>XM_002605803<br>XP_002605849	368aa<br>e1= 136<br>e2= 158<br>e3= 133<br>e4= 117<br>e5= 119<br>e6= 105<br>e7= 278<br>e8= 61	Exon 8 was predicted *de novo* and verified by PCR	269470*<br>Sc0000073<br><br>362aa<br>e1= 136<br>e2= 158<br>e3= 133<br>e4= 117<br>e5= 119<br>e6= 105<br>e7= 260<br>e8= 61		GPCR +<br>(52-310aa)<br>OPSIN +<br>(294-310aa)

B. floridae

```
>Bf_op14_1107bp
ATGGATATCCCGACGGAGACACCGTACGGAGCCGGGGACGATCCGGCTGGGACGGGCTGGCGTTGGGCGG
AAACGGACCAGAACGGCTTCCACAAGTACGATCATCTCATCGTGGGACTGTATCTGTTTGTCATTGGAAT
TATTGGTACTGTTGAAAATGGCATCACTCTGGCAACCTTCACGAAGTTCCGTTCGCTGAGGTCACCCACC
ACCATGCTGCTCGTACATCTGGCCATTGCAGACCTGGGCATCTGCATTTTTGGCTACCCGTTCTCTGGTG
CCTCCAGTCTGAGGTCCCACTGGCTGTTTGGCGGCGTGGGTTGCCAGTGGTACGGCTTTAACGGCATGTT
CTTCGGCATGGCGAACATCGGGCTGCTGACCTGCGTGGCTGTGGACCGGTACCTGGTCATCTGTCGGCAG
GATCTTGTTGACAAAGTGAACTACAACACGTACGGGGTGATGGCGGCTCTGGGATGGCTGTTCGCCGCCT
TCTGGGCAGCCCTGCCGCTGGTTGGCTGGGCAGAGTACTCTCTGGAACCTTCAGGTACGGCGTGCACCAT
CAACTGGCAGAAGAACGACAGCCTTTACATCTCCTACGTCACCTCCTGCTTCATCCTGGGCTTCGCCCTC
CCACTCGCCGTCATGATGTTCTGCTACTGGCAGGCCAGCTGTTTTGTCAATAAAGTACTGAAGGGAGACA
TCTCTGGAGACCTGACTTTTCCGGTCGCTGTTAATGTCGACTGGGAGTACCAGAACCACTTCTCAAAGAT
GTGCCTGGCCATGGTGGCAGCCTTTGTGGTGGCATGGACACCGTACTCCGTGCTGTTCCTCTTCGCCGCC
TTCGGGAACCCCGCGGACATCCCCGCCTGGATCACCCTGCTGCCCCCGCTCATCGCCAAGTCCTCGGCCC
TCTACAACCCCATCATCTACATCATCGCTAACCGCCGCTTCCGCAGCGCCATCTTCTCCATGGTGAAGGG
CCAGAACCCTGATGTGGAGCTGGAGGAAACTAACATACCCATGGACGCTGGCGACCACAGCTGCAGGATA
AGCCCGATTGAAGACACTGGCAAGGAGATGAGTTCCATGGGGAACGCCAATGCCTGA
```

```
>Bf_op14_368aa
MDIPTETPYGAGDDPAGTGWRWAETDQNGFHKYDHLIVGLYLFVIGIIGTVENGITLATFTKFRSLRSPT
TMLLVHLAIADLGICIFGYPFSGASSLRSHWLFGGVGCQWYGFNGMFFGMANIGLLTCVAVDRYLVICRQ
DLVDKVNYNTYGVMAALGWLFAAFWAALPLVGWAEYSLEPSGTACTINWQKNDSLYISYVTSCFILGFAL
PLAVMMFCYWQASCFVNKVLKGDISGDLTFPVAVNVDWEYQNHFSKMCLAMVAAFVVAWTPYSVLFLFAA
FGNPADIPAWITLLPPLIAKSSALYNPIIYIIANRRFRSAIFSMVKGQNPDVELEETNIPMDAGDHSCRI
SPIEDTGKEMSSMGNANA
```

B. belcheri

```
>Bb_op14_1089bp
ATGGATGCTCCGACAGAGACGCCGTATGAGCCTGAGGAAGATGTGGATGTGGCCGGGTGGCAGTGGACGG
AAACTGACAAGAACGGCATTCACAAATACGACCACCTCATCGTGGGACTGTACCTGTTTGTCATCGGTAT
```

```
CATCGGTACCATTGAAAACGGCGTCACTCTGGCAACATTCACGAAGTTCCGCCCCCTGAGGTCACCGACC
ACCATGCTACTCGTACATCTGGCCATAGCAGATTTGGGCATCTGTATTTTCGGCTACCCGTTTTCCGGGG
CCTCCAGTCTGAGGTCCCACTGGCTGTTTGGGGGTGTCGGCTGCCAGTGGTACGGCTTCAATGGCATGTT
CTTCGGCATGGCTAATATCGGACTGCTGACATGTGTGGCCGTGGACCGGTACCTGGTCATCTGCCGACAA
GACCTTGTCGACAAAGTAAACTACAACACCTACGGAGTGATGGCGGCCATGGGCTGGCTGTTTGCGGCCT
TCTGGGCAGCGCTGCCGCTGGTCGGCTGGGCAGAGTACGCCCTGGAGCCTTCAGGGACGGCATGCACTAT
CAACTTTCGGAAGAACGACAGCTTATACATCTCTTACGTCAGCAGCTGCTTCGTCCTGGGATTTCTCCTC
CCTCTCGCCGTTATGGCGTTCTGCTACTGGCAGGCCAGCTGCTTTGTCAGCAAGGTGCTGAAGGGAGACA
TTGCAGGAGATCTGACCTTTCCAGTCGCCATGAATGTTGACTGGGAGTATCAGAACCATTTCTCAAAGAT
GTGCCTGGCCATGGTGGCAGCGTTTGTGGTGGCCTGGACACCCTACTCCGTGTTGTTCCTCTTCGCCGCC
CTGGGAAACCCTGACGACATCCCCGCCTGGCTCACCCTGCTGCCTCCGCTCATCGCCAAGTCCTCGGCCC
TGTACAACCCCATCATCTACATCATCGCTAACCGGCGGTTCCGAAGTGCCATCTGCTCCATGATGAAGGG
TCAGAATCCTGGCATTGAAGATGACGAGGAACATGCTGACGATCATAGGATACACCCGAGTGAAGACAAT
GGGGTTATTTCCATGGTAACCCTGAACTTGCATCACTGA
```

```
>Bb_op14_362aa
MDAPTETPYEPEEDVDVAGWQWTETDKNGIHKYDHLIVGLYLFVIGIIGTIENGVTLATFTKFRPLRSPT
TMLLVHLAIADLGICIFGYPFSGASSLRSHWLFGGVGCQWYGFNGMFFGMANIGLLTCVAVDRYLVICRQ
DLVDKVNYNTYGVMAAMGWLFAAFWAALPLVGWAEYALEPSGTACTINFRKNDSLYISYVSSCFVLGFLL
PLAVMAFCYWQASCFVSKVLKGDIAGDLTFPVAMNVDWEYQNHFSKMCLAMVAAFVVAWTPYSVLFLFAA
LGNPDDIPAWLTLLPPLIAKSSALYNPIIYIIANRRFRSAICSMMKGQNPGIEDDEEHADDHRIHPSEDN
GVISMVTLNLHH
```

```
                                                                         TM I
 ####################
Bl_op14_366aa         1 MDVPTEMPYGVEDDA-DEGGWQWAQTDKNGFHKYDHLIVGLYLFVIGIIGTVENGVTLAT
Bf_op14_368aa         1 MDIPTETPYGAGDDP-AGTGWRWAETDQNGFHKYDHLIVGLYLFVIGIIGTVENGITLAT
Bb_op14_362aa         1 MDAPTETPYEPEEDV-DVAGWQWTETDKNGIHKYDHLIVGLYLFVIGIIGTIENGVTLAT
Amphiop3_BAC76023    1 MDIPTETPYGAEEDIGESAGWRWTETDKNGFHKYDHLIVGLYLFVIGIIGTIENGITLAT

TM II                                 TM
 ##          ######################                     ##########
Bl_op14_366aa        60 FTKFRSLRSPTTMLLVHLAIADLGICIFGYPFSGASSLRSHWLFGGVGCQWYGFNGMFFG
Bf_op14_368aa        60 FTKFRSLRSPTTMLLVHLAIADLGICIFGYPFSGASSLRSHWLFGGVGCQWYGFNGMFFG
Bb_op14_362aa        60 FTKFRPLRSPTTMLLVHLAIADLGICIFGYPFSGASSLRSHWLFGGVGCQWYGFNGMFFG
Amphiop3_BAC76023   61 FSKFRSLRSPTTMLLVHLAIADLGICIFGYPFSGASSLRSHWLFGGVGCQWYGFNGMFFG

 III                                 TM IV
############                   #####################
Bl_op14_366aa       120 MANIGLLTCVAVDRYLVICRQDLADKVNYNTYGVMAALGWLFAAFWAALPLVGWGEYALE
Bf_op14_368aa       120 MANIGLLTCVAVDRYLVICRQDLVDKVNYNTYGVMAALGWLFAAFWAALPLVGWAEYSLE
Bb_op14_362aa       120 MANIGLLTCVAVDRYLVICRQDLVDKVNYNTYGVMAAMGWLFAAFWAALPLVGWAEYALE
Amphiop3_BAC76023  121 MANIGLLTCVAVDRYLVICRHDLVDKVNYNTYGVMAALGWLFAAFWAALPLVGWAEYALE

                                                  TM V
######################
Bl_op14_366aa       180 PSGTACTINWRKNDSLFTSYVTTCFTLGFALPLAVMMFCYLQASWFVSKVLKGDISGDLT
Bf_op14_368aa       180 PSGTACTINWQKNDSLYISYVTSCFILGFALPLAVMMFCYWQASCFVNKVLKGDISGDLT
Bb_op14_362aa       180 PSGTACTINFRKNDSLYISYVSSCFVLGFLLPLAVMAFCYWQASCFVSKVLKGDIAGDLT
Amphiop3_BAC76023  181 PSGTACTINFQKNDSLYISYVTSCFVLGFVVPLAVMAFCYWQASCFVSKVLKGDIAGDLT

                                             TM VI
  ######################               #######
Bl_op14_366aa       240 FPVASNVDWEYQNHFSKMCLAMVAAFVVAWTPYSVLFLFAAFGDPADIPGWITLLPPLIA
Bf_op14_368aa       240 FPVAVNVDWEYQNHFSKMCLAMVAAFVVAWTPYSVLFLFAAFGNPADIPAWITLLPPLIA
Bb_op14_362aa       240 FPVAMNVDWEYQNHFSKMCLAMVAAFVVAWTPYSVLFLFAALGNPDDIPAWLTLLPPLIA
Amphiop3_BAC76023  241 FPVAANVDWEYQNHFSKMCLAMVAAFVVAWTPYSVLFLFAAFWNPADIPAWLTLLPPLIA

TM VII
*#############
Bl_op14_366aa       300 KSSALYNPIIYIIANRRFRSAICSMLKGQDPDEEDP----NMAMSHEEHRVTTIEDTDKE
Bf_op14_368aa       300 KSSALYNPIIYIIANRRFRSAIFSMVKGQNPDVELEETNIPMDAGDHSCRISPIEDTGKE
```

```
Bb_op14_362aa        300 KSSALYNPIIYIIANRRFRSAICSMMKGQNPGIEDDEEHA------DDHRIHPSEDN--G
Amphiop3_BAC76023  301 KSSALYNPIIYIIANRRFRNAICSMMKGQDPDVEDD-EHA------DEHRVRSIEDNDKE

Bl_op14_366aa        356 TVAMHHMLNTIT
Bf_op14_368aa        360 MSSMGNANA--
Bb_op14_362aa        352 VISMVTLNLHH
Amphiop3_BAC76023  354 IISMVNLNMTV
```

op15 + AmphiMop

op15							
B. lanceolatum	***B. floridae***				***B.belcheri***		**ScanProsite[5]**
Genomic scaffold in Assembly BraLan2[1]	JGI gene model (Putative allele or duplicate)[2]	NCBI gene model, Transcript and protein Accession numbers	Exon No and size[3]	Comments	Predicted gene model & scaffold in HapV2 assembly[4]	Comments	
Sc0000013<br>(1161015..1170390)<br><br>662aa<br>e1= 185<br>e2= 134<br>e3= 134<br>e4= 94<br>e5= 200<br>e6= 202<br>e7= 158<br>e8= 90<br>e9= 201<br>e10= 219<br>e11= 372	**65960***<br>**(65959[d])**<br>scaffold_9	**65960***<br>Scaffold_167<br>(616aa)<br><br>XM_002596237<br>XP_002596283	 <br><br><br>657aa<br>e1= 182<br>e2= 134<br>e3= 134<br>e4= 94<br>e5= 200<br>e6= 202<br>e7= 158<br>e8= 90<br>e9= 198<br>e10= 219<br>e11= 363	There are 11 and not 12 exons as originally predicted in JGI/NCBI databases	041410/004820F<br>Sc0000000<br><br>653aa<br>e1= 179<br>e2= 134<br>e3= 134<br>e4= 94<br>e5= 200<br>e6= 202<br>e7= 158<br>e8= 90<br>e9= 189<br>e10= 219<br>e11= 363		GPCR +<br>(51-317)<br>OPSIN +<br>(301-317)

B. floridae

```
>Bf_op15_1974bp
ATGGAGGTGACGCCTACCCCAGGATGTACCGGCAACGCTAGCGTGTGTAACGGGACGGACTCTGGCGGGG
GAGTCGTGTGGGACATCCCGCCTCTGGCTCATTACATCGTGGGCACGGCTGTGTTCTGTGTCGGCTGCTG
CGGGATGTTCGGCAATGCTGTCGTGGTGTACTCCTTTATCAAATCTAAAGGCTTACGGACTCCTGCTAAC
TTCTTCATCATCAACCTTGCTCTCAGCGACTTTCTCATGAACCTCACAAACATGCCCATCTTTGCCGTTA
ATTCGGCTTTCCAACGCTGGCTTCTCAGTGACTTCGCTTGCGAGCTGTACGGTTTCGCTGGAGGGTTGTT
CGGATGTCTGTCCATCAACACGCTGATGGCCATCTCCATGGACCGGTACCTCGTCATCACCAAGCCTTTC
CTGGTCATGCGGATTGTTACCAAGCAACGGGTAATGTTTGCCATTCTCCTGCTCTGGATATGGTCCCTTG
TATGGGCTCTGCCTCCTCTGTTCGGATGGAGCGCCTATGTGCCTGAAGGATTCGGAACAAGCTGCACTTT
CGACTACATGACGCCGAAGCTGAGCTACCACATCTTCACGTACATCATCTTCTTCACCATGTACTTCATC
CCCATGGGCGTCATCATCTACTGCTATTACAACATCTTTGCCACCGTCAAGTCAGGAGACAAACAGTTCG
GCAAGGCCGTCAAGGAGATGGCTCACGAAGATGTGAAGAATAAGGCTCAACAAGAGAGGCAGCGAAAAAA
TGAGATCAAGACCGCAAAGATCGCCTTCATCGTCATCACCCTGTTCCTGTCGGCATGGACTCCGTATGCT
GTGGTATCTGCGCTAGGGACGCTGGGATACCAGGACTTGGTGACGCCATATTTGCAGTCCATTCCCGCCG
TGTTCGCCAAGTCGTCTGCCGTGTACAACCCTATTGTGTACGCCATTACTCATCCGAAGTTCCGTGCGGC
AGTAAAGAAGCACATCCCGTGCCTGTCAGGCTGTCTGCCTGCGGATGAGGAGGAGACCAAGACCAAGACC
CGCGGAGCCACAACAACGGCTTCCATGAGCATGACCCAAACCACTGCGCCCACTCATGATCCCCAGGCAA
GCGTGCACTCCGGGTCCAGCGTGTCGGTTGATGATAGCAGCGGCGTGTCTCGCCAGGATACAATGATGGT
GAAGGTGGAAGTGGACAATAGAATGGAGAAGGCCGGCGGCGGTGCAGCCGATACCGCTCCTAAGGATGGC
ACCAGTGTGCCGACTGTTTCCGCCCAGATCGAAGTGCGACCCTCTGGTAACGTCAACACTAAAGCTGAGG
TGATCCCGTCTCCTCAGTCTGCAGCGGTAGCACATGGAGCATCGGCAAGTCCTGTCCCTAAGGTTGCGGA
ATTAAGCTCCTCGGTCAGTTTGGAGAGTGCCGCCATTCCAGGTAAGATACCGACCCCCCTCCCCAGCCAG
CCAATAGCAGCACCGATTGAACGCCACATGGCCGCCATGGCTGATGATCCGCCTCCTAAGCCGAGAGGTG
TGGCCACTACAGTCAACGTGAGGCGCTCGGAGAGTGGCTATGAGCGCAGCCAGGACAGTCTACGGAAAAA
```

GGCGGTCAGTGAAACGCGCAGTCGCTCTTTCAACTCGACGAAGGACCACTTCGCATCTGAGCGACAGACG
TCGACAACGCTGAACCAGCCTAGGGACATGTACAGCGGCGACATGGTGAAAAAGACACGACAGTCCCCTG
AAAAACAAGAGTACGACAACCCCGCATTTGATGCAGGTATCGCTGAGATTGACACCGACTCTGAGAACGA
GACAGAAGGGTCGTATGACATGCTCTCTGTTCGCTTTCAGGCTATGGCAGAAGAGCCTCCTGTCGAAACA
TACAGGAAAGCCAGCGACATGTCAATCAATCTTGGCAAAGCCTCTCTTATGCTCACCGAGGCCCATGATG
AGACTGTTTTGTGA

>Bf_op15_657aa
MEVTPTPGCTGNASVCNGTDSGGGVVWDIPPLAHYIVGTAVFCVGCCGMFGNAVVVYSFIKSKGLRTPAN
FFIINLALSDFLMNLTNMPIFAVNSAFQRWLLSDFACELYGFAGGLFGCLSINTLMAISMDRYLVITKPF
LVMRIVTKQRVMFAILLLWIWSLVWALPPLFGWSAYVPEGFGTSCTFDYMTPKLSYHIFTYIIFFTMYFI
PMGVIIYCYYNIFATVKSGDKQFGKAVKEMAHEDVKNKAQQERQRKNEIKTAKIAFIVITLFLSAWTPYA
VVSALGTLGYQDLVTPYLQSIPAVFAKSSAVYNPIVYAITHPKFRAAVKKHIPCLSGCLPADEEETKTKT
RGATTTASMSMTQTTAPTHDPQASVHSGSSVSVDDSSGVSRQDTMMVKVEVDNRMEKAGGGAADTAPKDG
TSVPTVSAQIEVRPSGNVNTKAEVIPSPQSAAVAHGASASPVPKVAELSSSVSLESAAIPGKIPTPLPSQ
PIAAPIERHMAAMADDPPPKPRGVATTVNVRRSESGYERSQDSLRKKAVSETRSRSFNSTKDHFASERQT
STTLNQPRDMYSGDMVKKTRQSPEKQEYDNPAFDAGIAEIDTDSENETEGSYDMLSVRFQAMAEEPPVET
YRKASDMSINLGKASLMLTEAHDETVL

B. belcheri

>Bb_op15_1962bp
ATGGAGGTAACGCCTACGCCGGGATGTACCGGGAACGGTACCGTCTGCAACGGGACTGACTCCGGCGGTG
TGGTATGGGACATCCCGCCACTAGCTCACTACATGGTGGGGACGGCCGTGTTCTGTATCGGGTGTTGCGG
CATGTTCGGCAACGCTGTCGTGGTATACTCCTTCATCAAATCTAAAGGCTTACGGACTCCTGCTAACTTC
TTCATCATCAACCTTGCTCTCAGCGACTTTCTCATGAACCTCACCAACATGCCTATCTTTGCTGTTAACT
CGGCTTTTCAACGCTGGCTTCTCAGTGACTTTGCTTGTGAGTTGTACGGTTTTGCTGGCGGATTGTTCGG
ATGTTTGTCCATCAACACGCTGATGGCCATCTCCATGGATCGGTATCTCGTCATCACCAAGCCTTTCCTG
GTCATGCGGATTGTTACCAAGCAACGGGTGATGTTCGCCATCCTCCTGCTGTGGATCTGGTCTCTTGTAT
GGTCTCTTCCTCCCCTGTTTGGATGGAGCGCCTACGTGTCAGAAGGATTCGGAACGAGCTGCACTTTCGA
CTACATGACGCCGAAGCTGAGCTACCACATCTTCACTTACATCATCTTCTTCACCATGTACTTCATTCCC
ATGGGTGTCATCATCTACTGCTACTACAACATCTTCGCCACCGTCAAGTCAGGAGACAAACAGTTCGGCA
AGGCCGTCAAGGAGATGGCTCATGAGGATGTGAAGAATAAGGCTCAACAAGAGCGACAGCGGAAGAATGA
GATCAAGACCGCCAAAATCGCCTTTATCGTCATCACCCTGTTCATGTCGGCCTGGACTCCGTACGCCGTG
GTCTCCGCGCTAGGGACGCTGGGATATCAGGACCTAGTGACGCCATATTTGCAGTCCATTCCCGCCATGT
TTGCCAAGTCGTCTGCCGTGTACAATCCTATTGTGTACGCCATCACTCACCCGAAGTTCCGCGCGGCTGT
GAAGAAGCACATCCCGTGCCTGTCAGGCTGTCTGCCGGCGGATGAGGAGGAGACCAAGACAAAGACCCGA
GGAACCTCCGCTACTGCCTCCATGAGCATGACCCAAACCACCGCTCCAACTCATGACCCCCAGGCAAGTG
TGCACTCCGCATCTAGTGTGTCGGTGGACGAAGGCAGCGGCGTATCCCGCCAGGACACCATGATGGTTAA
GGTGGAAGTGGATAAGAGGATGGAAAAGGCCGAGGGAGGTGCAACGGAGTCCGCCCCGCAGGAGGGAGCC
AGTGTACCGACTGTTTCTGCTCAGATAGAAGTGCGGCCCTCTGGGAACGTGACTACCAAGGCGGAGGTGA
TTCCGTCACCACAGACTTCTCATGGCTCATCGGCAAGCCCTGTTCCAAAGGTAGCTGAACTGAGCTCGTC
TGCCACCCTGGAGAGCGCCGCTATCCCCGGAAAGATCCCCACACCCCTCCCCAGCCAGCCGATAGCAGCT
CCGATCGAGCGTCACATGGCCGCTATGGCTGATGAGCCGCCTCCGAAGCCGAGAGGAGTGGCCACTACAG
TGAATGTGAGGCGGACGGAGAGTGGGTATGATCGCAGTCAGGACAGCCAGCAGAAAAAGGTGGTTGGAGA
CACGCATCGCAGTCGATCTTTCAACTCGACAAAGGACCGATTCGACGCCGAGCGACCGGCAACACTGAAC
CAACCCGAAGGGATCCACAGCGGCGACACGGCAAAGAAGACAACCCGGCAGTCTTCCGACACACAAGAGT
ACGACAACCCCGCTTTTGACGCAGGCATCACTGAGGTTGACACAGACTCAGAGAACGAGACTGAGGGGTC
GTATGACATGCTCTCTGTGCGCTTCCAGGCAATGGCAGAAGAGCCCCCTGTGGAAACATACAGGAAAGCC
AGCGACTTGGCGATCAATCTTGGCAAAGCCTCGCTGATGCTTGCCGAGGCCCATGATGAAACGGTTTTGT
GA

>Bb_op15_653aa
MEVTPTPGCTGNGTVCNGTDSGGVVWDIPPLAHYMVGTAVFCIGCCGMFGNAVVVYSFIKSKGLRTPANF
FIINLALSDFLMNLTNMPIFAVNSAFQRWLLSDFACELYGFAGGLFGCLSINTLMAISMDRYLVITKPFL
VMRIVTKQRVMFAILLLWIWSLVWSLPPLFGWSAYVSEGFGTSCTFDYMTPKLSYHIFTYIIFFTMYFIP
MGVIIYCYYNIFATVKSGDKQFGKAVKEMAHEDVKNKAQQERQRKNEIKTAKIAFIVITLFMSAWTPYAV
VSALGTLGYQDLVTPYLQSIPAMFAKSSAVYNPIVYAITHPKFRAAVKKHIPCLSGCLPADEEETKTKTR
GTSATASMSMTQTTAPTHDPQASVHSASSVSVDEGSGVSRQDTMMVKVEVDKRMEKAEGGATESAPQEGA
SVPTVSAQIEVRPSGNVTTKAEVIPSPQTSHGSSASPVPKVAELSSSATLESAAIPGKIPTPLPSQPIAA
PIERHMAAMADEPPPKPRGVATTVNVRRTESGYDRSQDSQQKKVVGDTHRSRSFNSTKDRFDAERPATLN
QPEGIHSGDTAKKTTRQSSDTQEYDNPAFDAGITEVDTDSENETEGSYDMLSVRFQAMAEEPPVETYRKA

SDLAINLGKASLMLAEAHDETVL

```
Bl_op15_662aa        1 ----------------------------------------------------MEVTPTPG
Bf_op15_657aa        1 ----------------------------------------------------MEVTPTPG
Bb_op15_653aa        1 ----------------------------------------------------MEVTPTPG
AmphiMop_BAE00065    1 MTEIPSFQPPINATEVEEENAVFPTALTEWFSEVGNQVGEVALKLLSGEGDGMEVTPTPG

                                                       TM I
 #######################
Bl_op15_662aa        9 CTGNASVCNGTDSGGGGVVWDIPPLAHYIVGTAVFCVGCCGMFGNAVVVYSFIKAKGLRT
Bf_op15_657aa        9 CTGNASVCNGTDSG-GGVVWDIPPLAHYIVGTAVFCVGCCGMFGNAVVVYSFIKSKGLRT
Bb_op15_653aa        9 CTGNGTVCNGTDS--GGVVWDIPPLAHYMVGTAVFCIGCCGMFGNAVVVYSFIKSKGLRT
AmphiMop_BAE00065   61 CTGNGSVCNGTDS--GGVVWDIPPLAHYIVGTAVFCIGCCGMFGNAVVVYSFIKSKGLRT

 TM II                                TM III
 ######################                ###################
Bl_op15_662aa       69 PANFFIVNLALSDLLMNLTNMPLFAVNSAFQRWLLSDFACELYGFAGGLFGCLSINTLMA
Bf_op15_657aa       68 PANFFIINLALSDFLMNLTNMPIFAVNSAFQRWLLSDFACELYGFAGGLFGCLSINTLMA
Bb_op15_653aa       67 PANFFIINLALSDFLMNLTNMPIFAVNSAFQRWLLSDFACELYGFAGGLFGCLSINTLMA
AmphiMop_BAE00065  119 PANFFIINLALSDFLMNLTNMPIFAVNSAFQRWLLSDFACELYGFAGGLFGCLSINTLMA

                                                     TM IV
###                     ######################
Bl_op15_662aa      129 ISMDRYLVITKPFLVMRIVTKQRVMFAILLLWVWSLVWSLPPLFGWGAYVSEGFGTSCSF
Bf_op15_657aa      128 ISMDRYLVITKPFLVMRIVTKQRVMFAILLLWIWSLVWALPPLFGWSAYVPEGFGTSCTF
Bb_op15_653aa      127 ISMDRYLVITKPFLVMRIVTKQRVMFAILLLWIWSLVWSLPPLFGWSAYVSEGFGTSCTF
AmphiMop_BAE00065  179 ISMDRYLVITKPFLVMRIVTKQRVMFAILLLWIWSLVWALPPLFGWSAYVSEGFGTSCTF

 TM V
  ######################
Bl_op15_662aa      189 DYMTPKLSFHIFTYFIFFTMYFIPLGVIIYCYYNIFATVKSGDKQFGKAVKEMAHEDVKN
Bf_op15_657aa      188 DYMTPKLSYHIFTYIIFFTMYFIPMGVIIYCYYNIFATVKSGDKQFGKAVKEMAHEDVKN
Bb_op15_653aa      187 DYMTPKLSYHIFTYIIFFTMYFIPMGVIIYCYYNIFATVKSGDKQFGKAVKEMAHEDVKN
AmphiMop_BAE00065  239 DYMTPKLSYHIFTYIIFFTMYFIPGGVMIYCYYNIFATVKSGDKQFGKAVKEMAHEDVKN

                                         TM VI                           TM
######################                 ######*
Bl_op15_662aa      249 KAQQERQRKNEIKTAKISFIVITLFISAWTPYAVVAALGTLGYQHLVTPYLQSIPAVFAK
Bf_op15_657aa      248 KAQQERQRKNEIKTAKIAFIVITLFLSAWTPYAVVSALGTLGYQDLVTPYLQSIPAVFAK
Bb_op15_653aa      247 KAQQERQRKNEIKTAKIAFIVITLFMSAWTPYAVVSALGTLGYQDLVTPYLQSIPAMFAK
AmphiMop_BAE00065  299 KAQQERQRKNEIKTAKIAFIVISLFMSAWTPYAVVSALGTLGYQDLVTPYLQSIPAMFAK

  VII
 #############
Bl_op15_662aa      309 SSAVYNPIVYAITHPKFRAAVKKHIPCLSGCLPADEEETKTKTRGAT-TSASMSMTQTTA
Bf_op15_657aa      308 SSAVYNPIVYAITHPKFRAAVKKHIPCLSGCLPADEEETKTKTRGAT-TTASMSMTQTTA
Bb_op15_653aa      307 SSAVYNPIVYAITHPKFRAAVKKHIPCLSGCLPADEEETKTKTRGTS-ATASMSMTQTTA
AmphiMop_BAE00065  359 SSAVYSPIVYAITYPKFREAVKKHIPCLSGCLPASEEETKTKTRGQSSASASMSMTQTTA

Bl_op15_662aa      368 PAHDPQASVDSTSSVSVDDSSGVSRQDTMMVKVEVDKRMEKAGGGASDSAPNEGASVPTV
Bf_op15_657aa      367 PTHDPQASVHSGSSVSVDDSSGVSRQDTMMVKVEVDNRMEKAGGGAADTAPKDGTSVPTV
Bb_op15_653aa      366 PTHDPQASVHSASSVSVDEGSGVSRQDTMMVKVEVDKRMEKAEGGATESAPQEGASVPTV
AmphiMop_BAE00065  419 PVHDPQASVDSGSSVSVDDSSGVSRQDTMMVKVEVDKRMEKAGGGAADAAPQEGASVSTV

Bl_op15_662aa      428 SAQIEVRPSGSVTTKAEVIPSPRTAAAATGSASASPVPKVMELRSSANLESAAIPGKIPT
Bf_op15_657aa      427 SAQIEVRPSGNVNTKAEVIPSPQSAAVAHG-ASASPVPKVAELSSSVSLESAAIPGKIPT
Bb_op15_653aa      426 SAQIEVRPSGNVTTKAEVIPSPQTSH----GSSASPVPKVAELSSSATLESAAIPGKIPT
```

```
AmphiMop_BAE00065  479 SAQIEVRPSGKVTTKADVISTPQTAH----GLSASPVPKVAELGSSATLESAAIPGKIPT

Bl_op15_662aa       488 PLPSQPIAAPIERHMAAMADEPPPKPRGVATTVNVRRTESGYDRTQDSLRKKAVGDMQSS
Bf_op15_657aa       486 PLPSQPIAAPIERHMAAMADDPPPKPRGVATTVNVRRSESGYERSQDSLRKKAVSE----
Bb_op15_653aa       482 PLPSQPIAAPIERHMAAMADEPPPKPRGVATTVNVRRTESGYDRSQDSQQKKVVGDT---
AmphiMop_BAE00065  535 PLPSQPIAAPIERHMAAMADEPPPKPRGVATTVNVRRTESGYDRSQDSQRKKVVGDT---

Bl_op15_662aa       548 FNRSRSFNSTKDHFASERQT--PLNQPKEIYTTDMAKK-TRQSPNKQEYDNPAFDAGITE
Bf_op15_657aa       542 -TRSRSFNSTKDHFASERQTSTTLNQPRDMYSGDMVKK-TRQSPEKQEYDNPAFDAGIAE
Bb_op15_653aa       539 -HRSRSFNSTKDRFDAERPA--TLNQPEGIHSGDTAKKTTRQSSDTQEYDNPAFDAGITE
AmphiMop_BAE00065  592 -HRSRSFNTTKDHFASEQPA--ALIQPKELYSDDTTKKMARQSSEKHEYDNPAFDEGITE

Bl_op15_662aa       605 IDTDSENETEGSYDMLSVRFQAMAEEPPVETYRKASDVAINLGKASLMLTEAHDETVL
Bf_op15_657aa       600 IDTDSENETEGSYDMLSVRFQAMAEEPPVETYRKASDMSINLGKASLMLTEAHDETVL
Bb_op15_653aa       596 VDTDSENETEGSYDMLSVRFQAMAEEPPVETYRKASDLAINLGKASLMLAEAHDETVL
AmphiMop_BAE00065  649 VDTDSENETEGSYDMLSVRFQAMAEEPPVETYRKASDLAINLGKASLMLSEAHDETVL
```

op16

op16							
B. lanceolatum	***B. floridae***				***B.belcheri***		**ScanProsite**[5]
Genomic scaffold in Assembly BraLan2[1]	JGI gene model (Putative allele or duplicate)[2]	NCBI gene model, Transcript and protein Accession numbers	Exon No and size[3]	Comments	Predicted gene model & scaffold in HapV2 assembly[4]	Comments	
Sc0000242 (291516..293213)<br><br>295aa<br>e1= 185<br>e2= 134<br>e3= 746	**86640*** scaffold_153 (86644[d])	**86640***(1005aa) scaffold_100<br><br>XM_002603763<br>XP_002603809	324aa<br>e1= 185<br>e2= 134<br>e3= 656		049200* Sc0000109<br><br>327aa<br>e1= 185<br>e2= 134<br>e3= 665		GPCR + (52-303aa) OPSIN – **K296** is at position 293

B. floridae

```
>Bf_op16_975bp
ATGGATGTCAGAAATATTTCCATGTGCTTGGGCTCATTGATCGAAGACAGCAGAGAGGCAAACGTCTCAG
CGGCGGGAATCGTGGAGCCGTCCGCCAAGACTCAGACCGTGTTCGGTGTGCTGATCCTCCTGGCTGGGCT
GACTGGGGTCCTCGGGAACGGCCTGGCTCTGCAAGCTTTTTATGCGTGTAAGGCCCTGCGAAAACCCAAG
CACTACCTGGTGGTGAATCTGAGCATCACAGACGGCCTGTTGTGTGTGGTGTTCTGTCCTGTCACAGTCT
GGGCAAGCTTCGCACACTCATGGACGTTTGGGAAACCAGGTCTGGACATGATCGGCTTCAGCGTTGGTGT
GATGGTCATAGTGTTGATGACCACCCAACTCGCCATAGCCGTACAGCGACTAGCCGTGTCCATCAAACCG
CTGAAGGCTGCCGTGTACATCACACACGGCAGGATGCTGCTCACCGCTGCCACCACATGGATCTACTCCG
CCCTGCTCATGCTGCCGCCTCTCCTGGGATGGAACAGATTCATCCTAGATCACAGTGGTGTCAGTGTAAC
GTTTGATTACTTAGCTCGTGACGACCTCTCAAGGCTATACGTCATTGTCTTGTTGATTTTCGCCTTTGCG
CTACCGTTAGTCGGCATAGTCTGTTCTTACAGCTACATTGTTGTGGCTGTGAGAAGGTCAAGGCAGAAGG
CGAAAGTGCCGACGACAGAGTCGACTTGTAAAAGAGATACGAAGACTGCCATTGTCGCACTGGGTCTCTC
GGCCTTGTTCTGTGCAAGTTGGACACCATATGCCATTGTTGTTCTCTTAAGTCTGTTTAACGTCACTGTT
CCTATTATCTACATCATGGTTGCTTCAGCCATAGCGAAGTGTTCATGCACAGTGAATCCCGTGATGTTTG
CGTTGACTCTACCTGTGGTCCGACAGTATTACAAGGAAAAGCTGGACAAATATCTCAGATTGTGA
```

```
>Bf_op16_324aa
MDVRNISMCLGSLIEDSREANVSAAGIVEPSAKTQTVFGVLILLAGLTGVLGNGLALQAFYACKALRKPK
HYLVVNLSITDGLLCVVFCPVTVWASFAHSWTFGKPGLDMIGFSVGVMVIVLMTTQLAIAVQRLAVSIKP
LKAAVYITHGRMLLTAATTWIYSALLMLPPLLGWNRFILDHSGVSVTFDYLARDDLSRLYVIVLLIFAFA
LPLVGIVCSYSYIVVAVRRSRQKAKVPTTESTCKRDTKTAIVALGLSALFCASWTPYAIVVLLSLFNVTV
PIIYIMVASAIAKCSCTVNPVMFALTLPVVRQYYKEKLDKYLRL
```

B. belcheri

```
>Bb_op16_984bp
ATGGATATTAGAAATATTTCCGAGCGCTTATTTCCGGAAGACAGCCCGATGGTGGAAAATCTCACAGCAG
CGGGAACAGTTGAGGAGCCGCCGCCGACTGCCCAGACTGTGTTCGGGGTGGTCCTTCTCGTGGCTTGTTT
GACTGGCGTCCTCGGAAACGGACTGGCTCTACAGGCTTTATACGCGTGTAAGGCCTTGCGGCAGCCTAAG
CACTACCTGGTGGCTAACCTGTGCGTGACAAACGGACTGATGTGCCTGGTGTACTGCTGCTCCACCGTCC
CCGCCAGCTTCGCACACACATGGCCGTTTGGAGAACTCGGTCGCGACGTGGTGGGCTTCAGCGTCAGTGT
GATGACCGTGGTGCTGATGACGTCACAGCTCGCCATAGCCGCACAGCGAGTCGCCGTCGCCGCCAGCCCC
CTAGCGGCCGCCTACAGCATCACACACGGCAAGATGCTGACGTGCGTCGCCGCCACATGGGTCTACTCAA
CCGTCGTTATGCTGCCTCCTCTCGCAGGGTGGAACAGGTTTGTCGTAGACCGCAGTGGCGTTAGCGTAAC
GTTTGACTACGCGGCTCTTGACGGTATTTCCAGGGCGTACACCTTTTCCTTGCTCGTCCTCGCCTTTGTA
```

```
GTACCGTTACTCGGTATAGTCTGTTGTTACAGCTACATCTTTGTCACCGTGAGAAGGTCTAGGCGAGAGG
CGAAGCTGAAGCATACAGAGTTGACTAGTAGAAGGGACAGAAAGACTGTGGTTGTCGCCATGTGCCTGAC
GGCTCTGTTCTGTGTAAGTTGGACGCCGTGCGCATCCGTAGTCGTTTTAACTCAGTGTAAAGTAACGGTT
TCTGTTACGTATGTCATGGTGGCTGCCGCCCTAGCGAAGTGCGCTTCTTCTATCGATCCGATGATGTTTG
CGTTTTCGCTACCTGTGGTCCGAAAGTATTACTGTAACAGGAAAAGGCAAAAGGAAAAATACATTGTAAC
CTAG
```

```
>Bb_op16_327aa
MDIRNISERLFPEDSPMVENLTAAGTVEEPPPTAQTVFGVVLLVACLTGVLGNGLALQALYACKALRQPK
HYLVANLCVTNGLMCLVYCCSTVPASFAHTWPFGELGRDVVGFSVSVMTVVLMTSQLAIAAQRVAVAASP
LAAAYSITHGKMLTCVAATWVYSTVVMLPPLAGWNRFVVDRSGVSVTFDYAALDGISRAYTFSLLVLAFV
VPLLGIVCCYSYIFVTVRRSRREAKLKHTELTSRRDRKTVVVAMCLTALFCVSWTPCASVVVLTQCKVTV
SVTYVMVAAALAKCASSIDPMMFAFSLPVVRKYYCNRKRQKEKYIVT
```

```
                                                                   TM I
                                                          ######################
Bl_op16_354aa    1 MDVRNIS--RRSFFEDNREVNSSAAGISVDGPSRTAQIVFGVVILLAGLTGVLGNGLALH
Bf_op16_324aa    1 MDVRNISMCLGSLIEDSREANVSAAGIV--EPSAKTQTVFGVLILLAGLTGVLGNGLALQ
Bb_op16_327aa    1 MDIRNIS--ERLFPEDSPMVENLTAAGTVEEPPPTAQTVFGVVLLVACLTGVLGNGLALQ

                                         TM II
                                 ######################                #########
Bl_op16_354aa   59 ALYACRALRNPKHYLVVNLCIAGGLLCVVYCPVTVWASFAHTWPFGQLGCDMFGFSVSVM
Bf_op16_324aa   59 AFYACKALRKPKHYLVVNLSITDGLLCVVFCPVTVWASFAHSWTFGKPGLDMIGFSVGVM
Bb_op16_327aa   59 ALYACKALRQPKHYLVANLCVTNGLMCLVYCCSTVPASFAHTWPFGELGRDVVGFSVSVM

                    TM III                                   TM IV
                    #############                    ######################
Bl_op16_354aa  119VIVLMATQLAIAVQRFAVAIKPLTAAVSITRGRMLLSAAFTWLYSALLMLPPLLGWNRFI
Bf_op16_324aa  119 VIVLMTTQLAIAVQRLAVSIKPLKAAVYITHGRMLLTAATTWIYSALLMLPPLLGWNRFI
Bb_op16_327aa  119 TVVLMTSQLAIAAQRVAVAASPLAAAYSITHGKMLTCVAATWVYSTVVMLPPLAGWNRFV

                                                   TM V
                                          ######################
Bl_op16_354aa  179IDQSGVSVMFDYLALDGLSRAYVIVLVVFAFAVPLIGIVCCYMYIFFAVRRSRQDAN---
Bf_op16_324aa  179 LDHSGVSVTFDYLARDDLSRLYVIVLLIFAFALPLVGIVCSYSYIVVAVRRSRQKAKVPT
Bb_op16_327aa  179 VDRSGVSVTFDYAALDGISRAYTFSLLVLAFVVPLLGIVCCYSYIFVTVRRSRREAKLKH

                                          TM VI                             TM
                                 ######################            #######*#####
Bl_op16_354aa  236 --VTCKREAKTALVALRYSALFCASWTPFAAVVLLTQCKVTVSINFGMVASAISKCSSTI
Bf_op16_324aa  239 TESTCKRDTKTAIVALGLSALFCASWTPYAIVVLLSLFNVTVPIIYIMVASAIAKCSCTV
Bb_op16_327aa  239 TELTSRRDRKTVVVAMCLTALFCVSWTPCASVVVLTQCKVTVSVTYVMVAAALAKCASSI

                    VII
                    #######
Bl_op16_354aa  294 HPMMFALSLPVVRRYHKVAWSPALLASVRFPSSAPRQRSLGSGRKLIRLDSRLSHGGKTG
Bf_op16_324aa  299 NPVMFALTLPVVRQYYKEKLDKYLRL----------------------------------
Bb_op16_327aa  299 DPMMFAFSLPVVRKYYCNRKRQKEKYIVT-------------------------------


Bl_op16_354aa  354 N
Bf_op16_324aa      -
Bb_op16_327aa      -
```

op17a+op17b

op17a							
B. lanceolatum	***B. floridae***				***B.belcheri***		**ScanProsite**[5]
Genomic scaffold in Assembly BraLan2[1]	JGI gene model (Putative allele or duplicate)[2]	NCBI gene model, Transcript and protein Accession numbers	Exon No and size[3]	Comments	Predicted gene model & scaffold in HapV2 assembly[4]	Comments	
Sc0000011 (2130885..2133960)<br><br>335aa<br>e1= 191<br>e2= 134<br>e3= 683	**201585*** scaffold_7 (175449[a] scaffold_507)	**201585*** scaffold_196 (278aa)<br><br>XM_002592905<br>XP_002592951	 <br><br>328aa<br>e1= 182<br>e2= 134<br>e3= 671		Sc0000116	**Op17a Ortholog is not present**	GPCR + (51-302aa) OPSIN – **K296** is at position 292
**op17b**							
Sc0000011 (2127552..2128936)<br><br>e1= 185<br>e2= 134<br>e3= 682	**Op17bOrtholog is not present**					**Op17bOrtholog is not present**	

B. floridae

\>Bf_op17a_987bp
ATGGACGTCGGAAATTTTACCGACCCCTCATTTCTCGAAGACGACAGAGAAAGAAATGATTTAGCGGCGG
CTATTGTTGACGAGCCGTCCCCCACTGCCCAGATAGTGTTTGGGGTGGCCATTCTTCTGGCTGGGGTCAC
AGGAATCATCGGGAACAGCCTAGCTCTTCAAGCTTTGTTTGCGTGTAAGGCCTTGCGAAGTCCGAAGCAC
TACCTGGTGCTGAACCTGTGCGTCACCAACGCCCTGCTGTGTGTGATGTACTGTTCTATCACGGTCTGGG
GAAGTTTCACACACACATGGACGGTTGGGGAAATCGGTCGCGACGTGATGGGCTTCAGTGTCAGTGTGAT
GACCATCGTCATGATGACCACCCAATTCGCCATAGCCGTCCAGCGGGTCGCCGTGGCCTTCAACCCTCTC
ACCGCGGCCGTCAACATCACGCGTGGCAAGATGCTGCTCACCGCTGCCACCACATGGATCTACTCCGCCC
TGATCATGTTACCGCCCCTTCTGGGATGGAACAGATTCATTATAGACCGCACCGGCGTCAGCGTCATGTT
TGACTACCTATCCTCCGACGGCTTCTCTAGGGCATACGTCATTGCGCTGTTGATTTTTGCATTTGTGCTG
CCGTTAATCGGTATAGTCTGTTGTTACAGCTACGTTCTTGTCGCAGTGAAGAGATCTAGGCAGAGCGCGA
ATGTGAAGAGTACAGAAAGGACTTGTAGAAGAGATAGGAAGACTGCTGTGGTCGCCCTGGCCCTCTCAGC
CTTGTTCTGTTTAAGTTGGGCGCCATATGCCATGGTGGTCCTCCTGAGTCTGTGTAAGATCACGGTTCCT
GTTATGTATGTCATGATAGCTAGCGCCATAGCGAAGTGTGCATCAACCATTGACCCAATGATGTTTGCGC
TGTCGCTACCCAAGGTCCGACAGTATTACCGAGAGAGAATGGGTAAACATTTCAGTGGACTTCTTTGTAG
AAAGTAG

\>Bf_op17a_328aa
MDVGNFTDPSFLEDDRERNDLAAAIVDEPSPTAQIVFGVAILLAGVTGIIGNSLALQALFACKALRSPKH
YLVLNLCVTNALLCVMYCSITVWGSFTHTWTVGEIGRDVMGFSVSVMTIVMMTTQFAIAVQRVAVAFNPL
TAAVNITRGKMLLTAATTWIYSALIMLPPLLGWNRFIIDRTGVSVMFDYLSSDGFSRAYVIALLIFAFVL
PLIGIVCCYSYVLVAVKRSRQSANVKSTERTCRRDRKTAVVALALSALFCLSWAPYAMVVLLSLCKITVP
VMYVMIASAIAKCASTIDPMMFALSLPKVRQYYRERMGKHFSGLLCRK

TM I

Suppl. file 1

```
                                                       #########################
Bl_op17a_335aa    1 MDVRNNFRSNRLLLGDNHREDNVSSAGLVDEPSACAQTVFGVVILLAGLTGILGSGLALQ
Bf_op17a_328aa    1 MDVG-NFTDPSFL--EDDRERNDLAAAIVDEPSPTAQIVFGVAILLAGVTGIIGNSLALQ

                                          TM II                                  TM
                    ###        ############################         ############
Bl_op17a_335aa   61 ALYRCKTLRNPKHYLTVNLCLTDGLLCVVYCPVTVWACFAHTWTFGEIGRNMFGFSVSVM
Bf_op17a_328aa   58 ALFACKALRSPKHYLVLNLCVTNALLCVMYCSITVWGSFTHTWTVGEIGRDVMGFSVSVM

                     III                                       TM IV
                    #############                    ########################
Bl_op17a_335aa  121 TLVLMTTQLAIAVQRLTVAFDPLTAVSTITHGKMLMTAAFTWLYSILLMLPPLLGWNRFV
Bf_op17a_328aa  118 TIVMMTTQFAIAVQRVAVAFNPLTAAVNITRGKMLLTAATTWIYSALIMLPPLLGWNRFI

                                                TM V
                                      #########################
Bl_op17a_335aa  181IDETGVSVMFDYLADDDLSRAYVIAMLIIAFVLPLIGVVCCYCYIFVAVRRSRQNAKVPA
Bf_op17a_328aa  178 IDRTGVSVMFDYLSSDGFSRAYVIALLIFAFVLPLIGIVCCYSYVLVAVKRSRQSANVKS

                                          TM VI                          TM VII
                                 #########################          ##########*#####
Bl_op17a_335aa  241SELTCKRDTKTAIVALGLSALFCVSWTPYAMVILLSLCNIAVPVTHVMVAAAIAKCSSTI
Bf_op17a_328aa  238 TERTCRRDRKTAVVALALSALFCLSWAPYAMVVLLSLCKITVPVMYVMIASAIAKCASTI


                    #######
Bl_op17a_335aa  301DPMMFALSLPAVRQFYKERLGKYFCDKSVKLGTSV
Bf_op17a_328aa  298 DPMMFALSLPKVRQYYRERMGKHFSGLLCRK----
```

op18

op18							
B. lanceolatum	***B. floridae***				***B.belcheri***		**ScanProsite[5]**
Genomic scaffold in Assembly BraLan2[1]	JGI gene model (Putative allele or duplicate)[2]	NCBI gene model, Transcript and protein Accession numbers	Exon No and size[3]	Comments	Predicted gene model & scaffold in HapV2 assembly[4]	Comments	
Sc0000212 (401872..406543)<br><br>349aa<br>e1= 215<br>e2= 128<br>e3= 707	**No predicted model**<br>scaffold_409 (scaffold_16)	**No predicted model**<br>scaffold_133	348aa<br>e1= 215<br>e2= 128<br>e3= 704		**No predicted model**<br>Sc0000016<br><br>350aa<br>e1= 218<br>e2= 128<br>e3= 707		GPCR + (62-311aa)<br>OPSIN –<br>**<u>K296</u>** is at position 301

B. floridae

```
>Bf_op18_1047bp
ATGGACTTCTTTCTCGACAATTCAAGCAACAGCCTTCCTGCCCTCTGTAAGGTACTGTTGACAGGAAACG
AGAGTGAGTTGGACGTGGGAAACCTGACAGACTGCGTCAGAGGGGAACGTTTCCACACCAGTCAGATTGT
GAATGCGACCCTTATCCTGTTGTTTGGAGTGACCGGAGCAGTGGGGAACACTTTGGCTCTCTACGCTTTT
ATCAGGGCTCTAAGGAAGCCTAAGAACTATCTGGTGGCCAACCTGTGCCTGAGCCAGTTGCTGATGTGTC
TAGCATACAGTCCGGTGACTGCTGTATCCAACTATCTACACAGATGGGTGGGTGGCTACATAGGCTGCCA
AGTGGTGGGCTTTCTGACTGGCATGGCGTGCATGGTGTCGATCCTGTCAATCACAGCCATCGCTAGGCAA
CGGTTGGGCGTGGTCAGGGCGCCACTTCACAGTCTCACAGCCTTCACACACTCAACAGAGCTGAAGCGAC
TGGCCCTCATTTGGCTGATATCCATAGTTTTGATGCTACCTCCCTTAACTGGATGGAACCGCTTTGTCGT
AGACCCTATCCAGTTCAGCGCTACGATGGATTACTTGTTGACTGACGCACCTTCGAAAGCGTACATTATA
GCTCTGATGGTCTGTGGGTTCTTTATCCCACTGGTGGACATTTGCTACTGCTATGGTTGTATACTGTACA
AAGTTGTTAAACTTGGACGTGGCAAACTCATTAGAAAGAGCACCAAAAAAGGGGTGAACGAGATCAGCAT
TGCACTTGGTACACTTATGATCACATCACTGTTCTGTGTCTGCTGGTTTCCATATATGGTTGTAGTGATT
CTGGGTATGTCAGAAGCATACATACCCCCAGAACTAGCTATGGCCGCCTCTCCACTTGCTAAACTGTCCA
CGACTATTAATTCCATCTTGTTTGCGCTTTCGCTGCCTGCTTTTCGAAGGCATTTCTTTCCGAGCAAGAA
AGTGTACAGGCCGAGTGCAACAGCTATGAAAACATATGACAGGTCAAAGAAGACCTGGAATTCATAA
```

```
>Bf_op18_348aa
MDFFLDNSSNSLPALCKVLLTGNESELDVGNLTDCVRGERFHTSQIVNATLILLFGVTGAVGNTLALYAF
IRALRKPKNYLVANLCLSQLLMCLAYSPVTAVSNYLHRWVGGYIGCQVVGFLTGMACMVSILSITAIARQ
RLGVVRAPLHSLTAFTHSTELKRLALIWLISIVLMLPPLTGWNRFVVDPIQFSATMDYLLTDAPSKAYII
ALMVCGFFIPLVDICYCYGCILYKVVKLGRGKLIRKSTKKGVNEISIALGTLMITSLFCVCWFPYMVVVI
LGMSEAYIPPELAMAASPLAKLSTTINSILFALSLPAFRRHFFPSKKVYRPSATAMKTYDRSKKTWNS
```

B. belcheri

```
>Bb_op18_1053bp
ATGGACAGCTCGTTTCTCGCCAATTCGAGCGACAGCTTTTCTGTCTTGTGTGAGTTAGCGTTGGCAGGAA
ACGAGAGCGTGTTGGACGAGGGAAACCTGACGCGCTGCATCGGTGGGGAACATTTCCGCACCAGTCAGAT
CGTCAACGCGACTCTCATCCTGCTGTTTGGAACAACGGGAGCCGTGGGAAACATGTTGGCCTTCTACGCT
TTCTTCAGGGCTCTAAGAAAGCCTAAAAACTACCTGGTGGCCAATCTGTGCTTGAGCCAGCTGCTCATGT
GCTTATCATACAGTCCAGTGACTGCAGTATCAAACTATCTTCACAGATGGGTGGAGGCTACATAGGGTG
CCAGGTGGTCGGCTTTCTCACTGGCGTGTCCTGTATGGTGTCCATCCTGTCAATCACTGCCATCGCTAGG
CAACAGCTGGGCGTGGTCAGGGCGCCTCTCCACAGCCTGACAGCCTTCAAGCACTCCACAGAGCTGAAGC
GAGTGGTCTTCATTTGGTTCATATCTATAGTTTGTATGCTACCGCCCTTATTTGGGTGGAACCGGTTTGT
TGTAAACCCTCTCCAGTTCAGTGCTACGATGGATTATTTGTCAACTGACGCATCGTCGAAGGCATATATT
GTAGTTTTGATGGTCTGTGGATTTTCTGTACCCCTGGCTTCTATTTGTCATAGCTACAGCTCTATATTCC
```

ACAGAGTTTTTAAACTTGGGCAAACCAAACATATCAGAAAAAGTGCCAAGAATGGAATCAATGGAATCAG
CGTTGCCCTTGCTGGACTCACCATTACATCACTCTTTTGTGTCTGCTGGTTTCCATATATGGTCGTAGTG
ATTCTGGGCTTGGCAGAAGCAGATATACCACCAGAACTAGACATGGCAGCCTCTCCACTTGCTAAACTGT
CAACCACTGTAAATTCCATTTTGTTTGCGCTTTCCCTGCCTGCTTTTCGAAGGTTTTTCTTTGGAGGCAA
AGAAAAATATAGGGAGAGTGTAATTGCTATGAAAACATATCACAGGTCCACCAAAAGTTGTGACAGGGTA
TAA

>Bb_op18_350aa
MDSSFLANSSDSFSVLCELALAGNESVLDEGNLTRCIGGEHFRTSQIVNATLILLFGTTGAVGNMLAFYA
FFRALRKPKNYLVANLCLSQLLMCLSYSPVTAVSNYLHRWVGGYIGCQVVGFLTGVSCMVSILSITAIAR
QQLGVVRAPLHSLTAFKHSTELKRVVFIWFISIVCMLPPLFGWNRFVVNPLQFSATMDYLSTDASSKAYI
VVLMVCGFSVPLASICHSYSSIFHRVFKLGQTKHIRKSAKNGINGISVALAGLTITSLFCVCWFPYMVVV
ILGLAEADIPPELDMAASPLAKLSTTVNSILFALSLPAFRRFFFGGKEKYRESVIAMKTYHRSTKSCDRV

```
                                                                            TM
                                                                 ##############
Bl_op18_349aa     1 MDS-FYDNSSDNSPVLCKLTSTGNESLSDTENLTDCVHAERFHIGQTVNATLILLFGATG
Bf_op18_348aa     1 MDF-FLDNSSNSLPALCKVLLTGNESELDVGNLTDCVRGERFHTSQIVNATLILLFGVTG
Bb_op18_350aa     1 MDSSFLANSSDSFSVLCELALAGNESVLDEGNLTRCIGGEHFRTSQIVNATLILLFGTTG

                    I                          TM II
                    ###########         #####################                  #
Bl_op18_349aa    60 TVGNTLAVYAFFRALRKPKNYLVANLCLGQLLMCLAYSPVTAVSNYLHRWVGGYIGCQVA
Bf_op18_348aa    60 AVGNTLALYAFIRALRKPKNYLVANLCLSQLLMCLAYSPVTAVSNYLHRWVGGYIGCQVV
Bb_op18_350aa    61 AVGNMLAFYAFFRALRKPKNYLVANLCLSQLLMCLSYSPVTAVSNYLHRWVGGYIGCQVV

                          TM III                                    TM IV
                    ####################                      ##################
Bl_op18_349aa   120GFLTGMSCMVSILSITAIARQRLGVVRAPLHSLTAFTHSAELTQLALIWFISIVSMLPPL
Bf_op18_348aa   120 GFLTGMACMVSILSITAIARQRLGVVRAPLHSLTAFTHSTELKRLALIWLISIVLMLPPL
Bb_op18_350aa   121 GFLTGVSCMVSILSITAIARQQLGVVRAPLHSLTAFKHSTELKRVVFIWFISIVCMLPPL

                                                             TM V
                                                     #####################
Bl_op18_349aa   180FGWNRFVIDSIQFSVTMDYLSTDISSKAYIVTLLVCGFFFPLVDICYCYSSIFYKVVKLG
Bf_op18_348aa   180 TGWNRFVVDPIQFSATMDYLLTDAPSKAYIIALMVCGFFIPLVDICYCYGCILYKVVKLG
Bb_op18_350aa   181 FGWNRFVVNPLQFSATMDYLSTDASSKAYIVVLMVCGFSVPLASICHSYSSIFHRVFKLG

                                               TM VI
                                      ######################                 ###
Bl_op18_349aa   240RSKVIRKSAKNGVNEISIALAGLLITSLFCICWLPYMVVVILGIAEVYIPPELALAASPL
Bf_op18_348aa   240 RGKLIRKSTKKGVNEISIALGTLMITSLFCVCWFPYMVVVILGMSEAYIPPELAMAASPL
Bb_op18_350aa   241 QTKHIRKSAKNGINGISVALAGLTITSLFCVCWFPYMVVVILGLAEADIPPELDMAASPL

                      TM VII
                    #*############
Bl_op18_349aa   300AKLSTTVNSILFALSLPAFRRYFFGSKKVYRGSAIAMKTYNKSVKHMGYV
Bf_op18_348aa   300 AKLSTTINSILFALSLPAFRRHFFPSKKVYRPSATAMKTYDRSKKTWNS-
Bb_op18_350aa   301 AKLSTTVNSILFALSLPAFRRFFFGGKEKYRESVIAMKTYHRSTKSCDRV
```

op19 + Amphiop6

op19							
B. lanceolatum	***B. floridae***				***B.belcheri***		**ScanProsite**[5]
Genomic scaffold in Assembly BraLan2[1]	JGI gene model (Putative allele or duplicate)[2]	NCBI gene model, Transcript and protein Accession numbers	Exon No and size[3]	Comments	Predicted gene model & scaffold in HapV2 assembly[4]	Comments	
Sc0000069<br>(1249266..1254369)<br><br>402 aa<br>e1= 164<br>e2= 114<br>e3= 931	**87094***<br>scaffold_158<br>(110002[a] scaffold_726)	**110002***<br>(334aa)<br>scaffold_726<br><br>XM_002586073<br>XP_002586119	**405aa**<br>e1= 164<br>e2= 114<br>e3= 940	Scaffold retained in NCBI is not the correct one	006470R*<br>Sc0000001<br><br>406 aa<br>e1= 170<br>e2= 114<br>e3= 937		GPCR +<br>(45-297aa)<br>OPSIN –<br>**K296** is at position 287

B. floridae

```
>Bf_op19_1218bp
ATGAGCCCCAACCTCACCAACACGTCCCTGCTGCCGAACCGGACGGACCGGCCCGAGCTGAGCCCGGCTG
ACGTCACCATGCAGCTGGTGTTCGGCTCGATGATGCTGGTCTTCGGGCTGATCGGAGTCGTCGGGAACGC
CGTCGCCTTGTACGCTTTCTGCAGATCCCGAAGTCTGAGGCGACCCAAGAACTACCTGATAGCTAACCTG
TGCCTGACGGACATGGTGGTCTGCCTGGTGTACAGTCCTATTATCGTCACCAGAAGTCTTAGTCATGGCT
TACCGTCCAAGGAAAGCTGCATCGTGGAGGGGTTTGTGGTGGGACTCGGGAGTATCGTCAGCATCTGCAG
CCTCGCCGGCATCGCTGTGGAGAGATACGTCACCATCACCCAGCCAATCAAATCCCTGTCCATTCTAACG
CACCGCGCTCTACTCGGGGCCGTCTCGGCCGTCTGGGTTTATGCCTTTCTCCTTGCATTTCCTCCGCTTG
TCGGATGGGGGCGCTACGTGAGCGAAGAGTCGAAGATCAGCTGCACCTTCGACTATCTTTCCACTGATGA
CGCCACCAGAGCACATGTTATCGTCCTTGTGATTGGTGCGTTCGGACTCCCGTTTTCCGTGATAACCTAT
TGCTACGTCCGTTCCTTCGCCACCGTCCGGAAGTGTACCAAGGAGAGAAAACAGATGTCCCCTCTGGCAA
AGTCAGATTCAAGGTCAGAGGTCAAAGCAGCGGTAAACTCCTTCGTCATCACTACGTCATTCTGCCTGTG
TTGGTGTCCCTACGCTGTGGTGGCGACCATGGGGGTATCAGGTTTCACTGTTCACAGCCACGCCGTCTTC
ATAGCCGCCCTCCTCGCCAAGCTGTCCGTACTCTTCAACCCAGTCGCTTATGTCCTGTCCATCCCGAGTT
TCAGAAAAGCGCTTTTCTCCTCCTCGAACGATCGGACAAAATATCAGACCGCGTTTACATTTGAAAGTTT
GGCAAAAACATCACCAGTGGAAAGAAAATGGTGTGCAGATTCGATAGAAAGATATCGAAGTAGCAACGTG
AACATCGAGTCCACAGAACTGACTGTACCGTACTCCGCATCGCGCGAGTCGTGCCTGCTCAGCCGTGCTG
CTACGGAGCGGCTCGCGGGGAGAAGCCCGTCTCTCACCGACATCGTGCGGGAGTTTGGTCTGCAGCAGAC
AGCTTCGCATCGAGAAACCTGGGTGTGA
```

```
>Bf_op19_405aa
MSPNLTNTSLLPNRTDRPELSPADVTMQLVFGSMMLVFGLIGVVGNAVALYAFCRSRSLRRPKNYLIANL
CLTDMVVCLVYSPIIVTRSLSHGLPSKESCIVEGFVVGLGSIVSICSLAGIAVERYVTITQPIKSLSILT
HRALLGAVSAVWVYAFLLAFPPLVGWGRYVSEESKISCTFDYLSTDDATRAHVIVLVIGAFGLPFSVITY
CYVRSFATVRKCTKERKQMSPLAKSDSRSEVKAAVNSFVITTSFCLCWCPYAVVATMGVSGFTVHSHAVF
IAALLAKLSVLFNPVAYVLSIPSFRKALFSSSNDRTKYQTAFTFESLAKTSPVERKWCADSIERYRSSNV
NIESTELTVPYSASRESCLLSRAATERLAGRSPSLTDIVREFGLQQTASHRETWV
```

B. belcheri

```
>Bb_op19_1221bp
ATGAGCCACAACCTGACCAACGTGTCGCTGGTGGCGAACCGGACGGATCCAGACCAGCCCGAGCTGAGTC
CGACCGACGTCACCATGCAGCTCATCTTCGGCTCCATGATGCTCGTCTTCGGGCTGATCGGAGTCGTCGG
GAACGTCGTCGCCTTGTACGCGTTTTGCAGAACTCGCAGTCTCAGAAGACCGAAGAACTACCTGGTTGCT
AACCTGTGTATGACGGACATGTTCGTGTGCCTGGTGTACTGTCCCATCATCGTTACCAGGAGCTTCAGTC
ACGGATTTCCTTCCAAGGAAAGCTGTGTTGTAGAGGGTTTTGTGGTGGGAGTCGGCAGCATTGCCAGCAT
```

```
CTGCAGCCTAGCCGCCATTGCTGTGGAACGGTATCTCACCATCACCCAGCCGTTCAAATCTCTGACGATC
TTAACCCAGCGAACACTTCTTGGCGCAGTTTTAACCGTGTGGGTCTACGCGCTTCTCCTTGCATTTCCAC
CGCTTGTCGGCTGGGGGCGCTACGTACGAGAGGAGACGTACGTCAGTTGCACCTTCGACTACCTGTCCAC
GGATGACGCCACAAGAGCGTATGTAACCACGCTTGTGATTGGTGCGTTTGGATTCCCGCTCCTCACGATA
GCCTATTGCTACATCCGTGTCTTCACGACCGCCAGGAAGCACGCAAAGGAGAGGAAATTCATCTCACCCC
TTCGAAAGCCAGAGGTAAGATCTGAGATCAAAACCGCCGTGAACGCTTGCGTCATAACGACGTCATTCTG
CCTTTGCTGGTGTCCCTACGCTGTCGTAGCTACCCTGGGCATCAGTGGGTACTACCAGGCTCAAAACCAG
GCTGTCTTCGCCGCAGCTCTGCTTGCTAAGTTGTCCGTCCTTTTCAACCCTATTGTTTACGTCTTGTCCA
TCCCAAATTTCCGCAAGGCCCTTTTTGCACAAGAACGGGAAAAACACACGCATGAAGATCTGGCACTGAC
CTCACCACCAGCAAACAAACGTGCGATCAAAAAGGTAGAAGGAAGTCCAAGCAGCGTCAGCAACGTCTAC
GCTGATCATGAATCTAACGAATGGGCTATGTCTTACTCAACCTCGCGTGAGACATGCTTGCTGAGCCGGG
CTGCCTCAAAGCGTCTGGCGGGAAAAACGAAGTCGATCGTGGATCTCGTGAATGAATTCGGACTTCAACA
GACAGCATTGTGTAAGGAAAGTTTGGTGTGA
```

```
>Bb_op19_406aa
MSHNLTNVSLVANRTDPDQPELSPTDVTMQLIFGSMMLVFGLIGVVGNVVALYAFCRTRSLRRPKNYLVA
NLCMTDMFVCLVYCPIIVTRSFSHGFPSKESCVVEGFVVGVGSIASICSLAAIAVERYLTITQPFKSLTI
LTQRTLLGAVLTVWVYALLLAFPPLVGWGRYVREETYVSCTFDYLSTDDATRAYVTTLVIGAFGFPLLTI
AYCYIRVFTTARKHAKERKFISPLRKPEVRSEIKTAVNACVITTSFCLCWCPYAVVATLGISGYYQAQNQ
AVFAAALLAKLSVLFNPIVYVLSIPNFRKALFAQEREKHTHEDLALTSPPANKRAIKKVEGSPSSVSNVY
ADHESNEWAMSYSTSRETCLLSRAASKRLAGKTKSIVDLVNEFGLQQTALCKESLV
```

```
                                                    TM I
######################
Bl_op19_402aa         1 MSHNLTNVSLPANRT--DQPELSPTDVTMQLIFGSMMLVFGLIGVIGNVVALYAFCRNHS
Bf_op19_405aa         1 MSPNLTNTSLLPNRT--DRPELSPADVTMQLVFGSMMLVFGLIGVVGNAVALYAFCRSRS
Bb_op19_406aa         1 MSHNLTNVSLVANRTDPDQPELSPTDVTMQLIFGSMMLVFGLIGVVGNVVALYAFCRTRS
Amphiop6_BAC76024     1 MSSNLTNVSLVANRT--DQTELSPTDVTMQLIFGSMMLVFGLIGVVGNVVALYAFCRTRS

                             TM II                            TM III
######################           ###################
Bl_op19_402aa        59 VRRPKNYLIANLCLTDLAVCLVYCPIIVTRSLSHGFPSEESCIVEGFVVGVGSVASIISL
Bf_op19_405aa        59 LRRPKNYLIANLCLTDMVVCLVYSPIIVTRSLSHGLPSKESCIVEGFVVGLGSIVSICSL
Bb_op19_406aa        61 LRRPKNYLVANLCMTDMFVCLVYCPIIVTRSFSHGFPSKESCVVEGFVVGVGSIASICSL
Amphiop6_BAC76024    59 LRRPKNYVVANLCLTDMFVCLVYCPIVVSRSFSHGFPSKESCIVEGFMVGVGSIASICSL

                                                    TM IV
 ####                      ######################
Bl_op19_402aa       119 VAIAVERYLTITKPMKSLTILTPRTLLGAVSAVWAYSLLLAGPPLVGWGRYVREKTDISC
Bf_op19_405aa       119 AGIAVERYVTITQPIKSLSILTHRALLGAVSAVWVYAFLLAFPPLVGWGRYVSEESKISC
Bb_op19_406aa       121 AAIAVERYLTITQPFKSLTILTQRTLLGAVLTVWVYALLLAFPPLVGWGRYVREETYVSC
Amphiop6_BAC76024  119AAIAVERYLSVTQPLKSLTILTQRKLLVAVLTVWVYSLLLAFPPLVGWGRYVREETYISC

                                   TM V
######################
Bl_op19_402aa       179 TFDYLSTDDATRAHVIMLVIGAFGFPLLIMTYCYIRVFTTVRKRAKERNIMSPLKKSESR
Bf_op19_405aa       179 TFDYLSTDDATRAHVIVLVIGAFGLPFSVITYCYVRSFATVRKCTKERKQMSPLAKSDSR
Bb_op19_406aa       181 TFDYLSTDDATRAYVTTLVIGAFGFPLLTIAYCYIRVFTTARKHAKERKFISPLRKPEVR
Amphiop6_BAC76024  179TFDYLSTDDATRAYVITLVMGAFGFPLLTIAYCYIRVFTTARKHAEERKFMSPLKRPESR

      TM VI                               TM VII
###################            #########*##########
Bl_op19_402aa       239 SEVKSAVTGCIITTSFCLCFCPYAVVASLGLSGFP-APSHAIFAAALLAKVTVLINPVVY
Bf_op19_405aa       239 SEVKAAVNSFVITTSFCLCWCPYAVVATMGVSGFT-VHSHAVFIAALLAKLSVLFNPVAY
Bb_op19_406aa       241 SEIKTAVNACVITTSFCLCWCPYAVVATLGISGYYQAQNQAVFAAALLAKLSVLFNPIVY
Amphiop6_BAC76024  239TEIKTAVTACVITTSFCLCWCPYAVVATLGISGVS-VQQQTVFSAALLAKLTVIINPIVY

      ###
Bl_op19_402aa       298 VFSIPSFRKALFANA--QTKKQTAYTGDGLIVPSPLVRERCTKSAEICRCSD----MDND
Bf_op19_405aa       298 VLSIPSFRKALFSSSNDRTKYQTAFTFESLAKTSPVERKWCADSIERYRSSN----VNIE
Bb_op19_406aa       301 VLSIPNFRKALFAQEREK------HTHEDLALTSPPANKRAIKKVEGSPSSVSNVYADHE
```

```
Amphiop6_BAC76024  298 VLSIPNFRKALFAQEREK------YASEDVVLTSLPGKTRRMKKVERSQSSNSNVVIE--


Bl_op19_402aa      352 -IQSPIAYSASRETCLVSLAATERLAERTNSILSLGKEFGLQDTASHQETWV
Bf_op19_405aa      354 STELTVPYSASRESCLLSRAATERLAGRSPSLTDIVREFGLQQTASHRETWV
Bb_op19_406aa      355 SNEWAMSYSTSRETCLLSRAASKRLAGKTKSIVDLVNEFGLQQTALCKESLV
Amphiop6_BAC76024  350 VKESSMAYSTSRESCLLSRAATKRLAGKTKSIVDLVDEFGLQETAPHKESLV
```

op20

op20							
B. lanceolatum	***B. floridae***				***B.belcheri***		**ScanProsite[5]**
Genomic scaffold in Assembly BraLan2)	JGI gene model (Putative allele or duplicate)[2]	NCBI gene model, Transcript and protein Accession numbers	Exon No and size[3]	Comments	Predicted gene model & scaffold in HapV2 assembly[4]	Comments	
Sc0000069 (1239309..1246463)<br><br>389aa<br>e1= 164<br>e2= 114<br>e3= 892	**110003*** scaffold_726	**110003*** scaffold_329 (398aa)<br><br>XM_002586074<br>XP_002586120	 <br><br>395aa<br>e1= 164<br>e2= 114<br>e3= 76<br>e4= 834		006460R<br>Sc0000001<br><br>391aa<br>e1= 170<br>e2= 114<br>e3= 892		GPCR + (45-295aa) OPSIN – **K296** is at position 285

B. floridae

```
>Bf_op20_1188bp
ATGAGCCCCAACCTCACCAACACGTCCCTGCTGCCGAACCGGACGGACCGGCCCGAGCTGACCCCGGCTG
ACGTCACCATGCAGCTGGTGTTCGGCTCGATGATGCTGGTCTTCGGGCTGATCGGAGTCGTCGGGAATGC
CGTCGCCTTGTACGCTTTCTGCAGTACTCGCAAACTGAGGAGACCCAAAAACTACGTGGTGGCAAACCTG
TGTCTGACAGACCTCATCATGTGCATCGTTTACTGTCCTGTTATCGTGATCAGTAGCTTCAGTGGAAGAA
TCCCGACAGATGGCGCGTGCACGATGGAAGGGTTCGTGGTCGGGATGGCGAGCATCGCGTCGGTTGGCAG
CCTGGTCGCCATAGCCGTGGAGCGGTTCTTCAGCATCACCCGGCCGATGAAATCTCTCACCATACTCACC
AAGCGGACTTTCCTAGGCGGAGTCGCTGTCGTGTGGCTCTACTCCCTCATTCTTGTCATACCACCGCTGC
TCGGCTGGGGGCGCTACGTGCGGGAGGAGACCAAGCTGAGCTGTTCCTTCGACTACCTGTCGACGGACGA
CGCGAACAGATCTTACGTCATCTGGCTCGTGATTGTCGCGTTCGGATTGCCTCTTCTCGTGATCGCGTAT
TGCTACATCAGCGTCTTCATCACCGTTAAAAAGTGCACGAAGAAGAGAAAACTCATGTCTCCCCACAAGA
AGTCCAGATCCGAAGTCAAGACTGCGGTAAACGCGTTCATCATGACCACAGCGTTCTGCCTGTGTTGGTG
TCCGTATGCTGTGGTGGCGACGATGGGAATCAGCGGTTCCTCTGTCCAAGGCACGGTGGTCTTCGGAGCT
GCTCTACTCGCCAAGCTGTCCGTACTCATCAACCCAGTTGCTTACGTGTTCTCCATTCCGAGTTTCCGCA
AGGCGCTGTTCGGACACAGGAAGAGAGGATACGGCACGTCCGATGGGCTGGCGAACGATTCCTCGTCAGA
GAAAAGACGGGGGAAGAAGCATGAATCCGATGCCAACAGCGCGACCGAGTATCTCAGACTGACTGTGTTT
TACTCGATGTACAGCCGAACGGGGCAGCTGAGTCCATGGGCATCCAAGCGGCTGGCAGGGAAGACCAAGT
CCATGCTGGATCTGACTACGGAATATGGACGGTTGGAAAGGACTGCGCACAAGGAGAGCTGGGTGTGA
```

```
>Bf_op20_395aa
MSPNLTNTSLLPNRTDRPELTPADVTMQLVFGSMMLVFGLIGVVGNAVALYAFCSTRKLRRPKNYVVANL
CLTDLIMCIVYCPVIVISSFSGRIPTDGACTMEGFVVGMASIASVGSLVAIAVERFFSITRPMKSLTILT
KRTFLGGVAVVWLYSLILVIPPLLGWGRYVREETKLSCSFDYLSTDDANRSYVIWLVIVAFGLPLLVIAY
CYISVFITVKKCTKKRKLMSPHKKSRSEVKTAVNAFIMTTAFCLCWCPYAVVATMGISGSSVQGTVVFGA
ALLAKLSVLINPVAYVFSIPSFRKALFGHRKRGYGTSDGLANDSSSEKRRGKKHESDANSATEYLRLTVF
YSMYSRTGQLSPWASKRLAGKTKSMLDLTTEYGRLERTAHKESWV
```

B. belcheri

```
>Bb_op20_1176bp
ATGAGCCACAACCTGACCAACGTGTCGCTGGGGGCGAACCGGACGGATCCGGACCAGCCCGAGCTGAGTC
CGACCGACGTCACCATGCAGCTCCTCTTCGGCTCCATGATGCTCGTCTTCGGGCTGATCGGAGTCGTCGG
GAACGTCGTCGCCTTGTACGCGTTTTGCAGTACTCGCAAACTGCGGAGACCCAAGAACTACGTGGTGGCT
AACCTGTGTCTGACTGACCTGGTCATGTGTATAGTCTACTGTCCGGTCATCGTCATCAGTAGCTTTAGTA
ACAGTGTCCCGACCGACGCGGCCTGCAGAATGGAGGGCTTTGTAGTCGGAATGGGGAGCATCGCGTCTGT
CGCTAGTCTGGTCGCCATCGCCGTGGAACGTTTCTTCAGCATCACCCAACCCATGAAATCCCTCACTATC
```

```
CTCACTAAACGGACGTTACTCGGCGCAGTCGCAGCCGTATGGGTCTACGCCGTAGCCCTTGTCGTTCCAC
CGTTTTTCGGATGGGGGCGCTACGTACGAGAAGTGACCGATCTGAGCTGTTCGTTCGACTACCTATCCAC
AGACGCCGCTAATAGAGCGTATGTCATGACGCTTGTGATTGGTGCGTTTGGACTCCCCCTCCTCACCATA
GCCTATTGCTACATCCGTGTCTTCACAACCGTCAAGAGGTGCTCGCAAAAGAGAAGACTGATGTCCCCCT
ACAAGAACTCTAAATCTGATGTCAAAACTGCAGTAAACGCGTTCATCATGACCACTGCGTTCTGTTTATG
CTGGTGCCCGTACACTGTTGTAGCCATCATCGGCGTTAGCGGTTCTCCTGTCCAGGACACAATCGTATTC
GGAGCCGCCATGCTTGCCAAGCTGTCTGTCCTCATAAACCCGGTTGCATACGTCTTTTCCATCCCTAGTT
TCCGCCAGGCCCTTTTCGGGCACAACAAACGGACAAACAACACATCGGAAGGGTTGGGGAATGATTCCTC
TTCAGAGAAAAGACGCTGGAAAAAGCGGGATGACGGTAGCGGCACGCAAAGTGAATACCACCAACTGACT
GTGTTTTACTCGGTTTCCAACATCGCCAGTCCGCTAAGTTCTGCAGCCTCTAAAAGACTGGCAGGGAGAA
CGAAGTCAATTGTTGATCTCGCAACAGAATATGGACAGAAAGAAAGCTGGGTATGA

>Bb_op20_391aa
MSHNLTNVSLGANRTDPDQPELSPTDVTMQLLFGSMMLVFGLIGVVGNVVALYAFCSTRKLRRPKNYVVA
NLCLTDLVMCIVYCPVIVISSFSNSVPTDAACRMEGFVVGMGSIASVASLVAIAVERFFSITQPMKSLTI
LTKRTLLGAVAAVWVYAVALVVPPFFGWGRYVREVTDLSCSFDYLSTDAANRAYVMTLVIGAFGLPLLTI
AYCYIRVFTTVKRCSQKRRLMSPYKNSKSDVKTAVNAFIMTTAFCLCWCPYTVVAIIGVSGSPVQDTIVF
GAAMLAKLSVLINPVAYVFSIPSFRQALFGHNKRTNNTSEGLGNDSSSEKRRWKKRDDGSGTQSEYHQLT
VFYSVSNIASPLSSAASKRLAGRTKSIVDLATEYGQKESWV
```

```
                                                             TM I
                                                    #######################
Bl_op20_389aa     1 MSHNLTNVSLPANRT--DQPELSSKDVTMQLIFGSMMLVFGLIGVIGNVVALYAFCSTRK
Bf_op20_395aa     1 MSPNLTNTSLLPNRT--DRPELTPADVTMQLVFGSMMLVFGLIGVVGNAVALYAFCSTRK
Bb_op20_391aa     1 MSHNLTNVSLGANRTDPDQPELSPTDVTMQLLFGSMMLVFGLIGVVGNVVALYAFCSTRK

                                   TM II                                     TM III
                           ######################              #################
Bl_op20_389aa    59 LRRPKNYVVANLCLTDLVMCLVYCPVIVFSSFSNSIPTDAACTMEGFVVGMASIASVGSL
Bf_op20_395aa    59 LRRPKNYVVANLCLTDLIMCIVYCPVIVISSFSGRIPTDGACTMEGFVVGMASIASVGSL
Bb_op20_391aa    61 LRRPKNYVVANLCLTDLVMCIVYCPVIVISSFSNSVPTDAACRMEGFVVGMGSIASVASL

                                                     TM IV
                    #####                    #####################
Bl_op20_389aa   119VAIAVERFFSITQPMKSLVIVTNRTFLGAVAAVWVYAFFLVVPPLLGWGRYVREETKLSC
Bf_op20_395aa   119 VAIAVERFFSITRPMKSLTILTKRTFLGGVAVVWLYSLILVIPPLLGWGRYVREETKLSC
Bb_op20_391aa   121 VAIAVERFFSITQPMKSLTILTKRTLLGAVAAVWVYAVALVVPPFFGWGRYVREVTDLSC

                                          TM V
                                 ######################
Bl_op20_389aa   179SFDYSSKDTANRAYVITLCILGFGFPLIIIAYCYIRVFTTVKKCSQKRRLMSPHKKSKSE
Bf_op20_395aa   179 SFDYLSTDDANRSYVIWLVIVAFGLPLLVIAYCYISVFITVKKCTKKRKLMSPHKKSRSE
Bb_op20_391aa   181 SFDYLSTDAANRAYVMTLVIGAFGLPLLTIAYCYIRVFTTVKRCSQKRRLMSPYKNSKSD

                                 TM VI                              TM VII
                         ######################            #######*#############
Bl_op20_389aa   239 VKTAVNAFIMTTAFCLCWCPYTVVAIIGASGSSVQGTVVFGAALLAKTSVLINPVAYVFS
Bf_op20_395aa   239 VKTAVNAFIMTTAFCLCWCPYAVVATMGISGSSVQGTVVFGAALLAKLSVLINPVAYVFS
Bb_op20_391aa   241 VKTAVNAFIMTTAFCLCWCPYTVVAIIGVSGSPVQDTIVFGAAMLAKLSVLINPVAYVFS

Bl_op20_389aa   299 IPSFRKALFGRNKRTWSTSNGPGNDSSSEKRRKL---DASASESGYIKLTVYSA---YSS
Bf_op20_395aa   299 IPSFRKALFGHRKRGYGTSDGLANDSSSEKRRGKKHESDANSATEYLRLTVFYSMYSRTG
Bb_op20_391aa   301 IPSFRQALFGHNKRTNNTSEGLGNDSSSEKRRWKKRDDGSGTQSEYHQLTVFYSVSNIAS

Bl_op20_389aa   353 TLSPAASKRLAGRTESMVDLASEFGRMGTRSHKESWV
Bf_op20_395aa   359 QLSPWASKRLAGKTKSMLDLTTEYGRLERTAHKESWV
```

```
Bb_op20_391aa   361 PLSSAASKRLAGRTKSIVDLATEYGQ------KESWV
```

op21

op21							
B. lanceolatum	***B. floridae***				***B.belcheri***		**ScanProsite**[5]
Genomic scaffold in Assembly BraLan2[1]	JGI gene model (Putative allele or duplicate)[2]	NCBI gene model, Transcript and protein Accession numbers	Exon No and size[3]	Comments	Predicted gene model & scaffold in HapV2 assembly[4]	Comments	
Sc0000322 (253878..258725)<br><br>295aa<br>e1= 101<br>e2= 134<br>e3= 123<br>e4= 132<br>e5= 155<br>e6= 243	**86195*** scaffold_149 (86253[a] Scaffold_150)	**86195*** scaffold_226 (326aa)<br><br>XM_002590477<br>XP_002590523	295aa<br>e1= 101<br>e2= 134<br>e3= 123<br>e4= 132<br>e5= 155<br>e6= 243	Scaffold retained in NCBI is not the correct one	033980* Sc0000117<br><br>297aa<br>e1= 101<br>e2= 134<br>e3= 123<br>e4= 132<br>e5= 155<br>e6= 249		GPCR + (24-284aa) OPSIN – **K296**is at position 274

B. floridae

```
>Bf_op21_888bp
ATGTTGAACCCGATCATCTTTTACAGCTTGGGAACGTTTGTTCTAGTGGTGGGCATCGTCGGCTGGATGG
GAAACCTGCTAATTATCTATGTCTTCTTCAGATCTAAACATTTCCACCGAACGAAGAACGTCCTGACGTT
GAACCTGGCCATCACGAACGCCCTGATGTGTGTAGCGACAGTGCCTACTTTCGTGGCGTCCAGCTTCAGT
CGAAAGTGGATCTTCAGTGACACAGTTTGCCAGATGACCGGTTTCATGGCAGGGTGGTTCGGCACACAGT
CTATCTGCACTCTGGCGGCGATCGCCCTCGACCGGTGCATCGCTATCACTCTGCCGATGACCGGTAAACT
AAGTCGCGGTCGCAGGGCGATAATAAGTATAGTGGTGGTGTGGCTGTGGTCCCTGGTCTGGTGTCTACCT
CCGTTCTTCGGCTGGAACAAGTGGTCCATGCAGAAATACCAGACAGGCTGCACCTTCAACTATTTGTCAG
ACAATACATTCCAAAGATGGTACATCATGGCCCTGACGTTAGCAGTCTTCGTACTGCCAATGGTGGTGAT
GATAGTGTGCTACTACGCCATCTGGCGGGCCGCACGGCGCAGCACCCTCGCACTCAAGGGCATGATGGAC
CTAGGGTCGTTTCAGAACCTGCCGAAAAAGCGAAGGAATGTCGACGTCCACACGGCCAAGGTCGGGGTGC
TGGTCACCTTGCTCTTCATCTTGTCCTGGACACCTTTCGCCATCGTGGCTCTGATTGGCTGGGCTGGATA
TGGTCACGTGCTAACACCTCTGGCGGGAGCCATTCCTGCTGCGATTGCCAAAATGTCAGTTGTCGTGAAC
TACGTCGTGTATGCAGCGCTGATGCCTGACTTCAAGCGAGGTAGGTAA
```

```
>Bf_op21_295aa
MLNPIIFYSLGTFVLVVGIVGWMGNLLIIYVFFRSKHFHRTKNVLTLNLAITNALMCVATVPTFVASSFS
RKWIFSDTVCQMTGFMAGWFGTQSICTLAAIALDRCIAITLPMTGKLSRGRRAIISIVVVWLWSLVWCLP
PFFGWNKWSMQKYQTGCTFNYLSDNTFQRWYIMALTLAVFVLPMVVMIVCYYAIWRAARRSTLALKGMMD
LGSFQNLPKKRRNVDVHTAKVGVLVTLLFILSWTPFAIVALIGWAGYGHVLTPLAGAIPAAIAKMSVVVN
YVVYAALMPDFKRGR
```

B. belcheri

```
>Bb_op21_894bp
ATGTTGGATCCGATCATTTTCTACTGCCTGGGGGCGTTCGTCCTACTGGTGGGCTTCGTCGGCTGGGTCG
GAAACCTGCTGATTGTTTATGTCTTCTTCAGATCTAAACATTTCCACCACACGAAGAACGTGCTGACGTT
AAACCTGGCCATCACGAACGTCTTAATGTGTTTAGCGACAGTTCCTACTTACGTGGCGTCAAGCTTCAGC
CGGAAGTGGATCTTCAGTGATACAGTTTGTCAGATGACGGGGTTCATGGCGGGGTGGTTCGGCACACAGT
```

```
CTATCTGCACGCTGGCGGCGATCGCGCTCGATCGGTGCATCGCTATCACTATGCCGCTGACCGGCAAACT
GAATCGGGGCCGAAGGATGGCGGTGAGTGTGGCGGTAGTGTGGCTGTGGTCGTTGGTCTGGTGTTTACCG
CCGTTCTTCGGCTGGAACAAGTGGTCCAAGCTGAAATACCAGACAAGCTGCACCTTCGACTACCTATCAG
GAAACACGTTCCAGAGATGGTACATCATGGCCCTGACACTGGCAGGCTTCGTCCTGCCCATGGTGGTGAT
GGTAGTGTGCTACTACGCCATCTGGCGGGCCGCGCGGCGCAGCACCCTGGCGCTGAAGAGCATGATGGAC
AAAGGGTCATTTCAGAAGTTACCGAAGAAGCGGAGACAAGTGGACGTACACACCGCCAAGATCGGGCTGC
TGGTCACCATGCTGTTCATCCTGGCCTGGACACCCTTCGCCATCGTGGCTCTGATTGGCTTTGCTGGGTA
CGGTCACCTGCTAACGTTAAACCCTCTTGCCGGAGCCATTCCGGCTGCAATCGCCAAAGGGGCCGTTGTC
GTGAACTTCGTCGTGTATGCGGCGCTGATGCCTGACTTCCAGCGAGGTACGTAA
```

```
>Bb_op21_297aa
MLDPIIFYCLGAFVLLVGFVGWVGNLLIVYVFFRSKHFHHTKNVLTLNLAITNVLMCLATVPTYVASSFS
RKWIFSDTVCQMTGFMAGWFGTQSICTLAAIALDRCIAITMPLTGKLNRGRRMAVSVAVVWLWSLVWCLP
PFFGWNKWSKLKYQTSCTFDYLSGNTFQRWYIMALTLAGFVLPMVVMVVCYYAIWRAARRSTLALKSMMD
KGSFQKLPKKRRQVDVHTAKIGLLVTMLFILAWTPFAIVALIGFAGYGHLLTLNPLAGAIPAAIAKGAVV
VNFVVYAALMPDFQRGT
```

```
                                     TM I                               TM II
                             #######################          #################
Bl_op21_295aa     1 MLDPIIFYSLGAFVLVVGIVGWIGNLLIIYVFFRSKHFHHTKNVLTLNLAITNVLMCLAT
Bf_op21_295aa     1 MLNPIIFYSLGTFVLVVGIVGWMGNLLIIYVFFRSKHFHRTKNVLTLNLAITNALMCVAT
Bb_op21_297aa     1 MLDPIIFYCLGAFVLLVGFVGWVGNLLIVYVFFRSKHFHHTKNVLTLNLAITNVLMCLAT

                                                TM III
                   #####                ######################
Bl_op21_295aa    61 VPTFVTSSFSRKWIFSDTVCQMTGFMAGWFGTQSICTLAAIALDRCIAITMPLTGKLNRG
Bf_op21_295aa    61 VPTFVASSFSRKWIFSDTVCQMTGFMAGWFGTQSICTLAAIALDRCIAITLPMTGKLSRG
Bb_op21_297aa    61 VPTYVASSFSRKWIFSDTVCQMTGFMAGWFGTQSICTLAAIALDRCIAITMPLTGKLNRG

                              TM IV                                          TM
                     #####################                           ##########
Bl_op21_295aa   121RRVTISVAVVWLWSLIWCLPPFFGWNKWSMQKYQTGCTFNYLSDNAFQRWYIMALTLAVF
Bf_op21_295aa   121 RRAIISIVVVWLWSLVWCLPPFFGWNKWSMQKYQTGCTFNYLSDNTFQRWYIMALTLAVF
Bb_op21_297aa   121 RRMAVSVAVVWLWSLVWCLPPFFGWNKWSKLKYQTSCTFDYLSGNTFQRWYIMALTLAGF

                    V
                   ############                                       #########
Bl_op21_295aa   181ILPMVVMIVCYYAIWRAARRSTLALKGMMDRRTFQKISKKRRHVDVHTAKVGVLVTLLFI
Bf_op21_295aa   181 VLPMVVMIVCYYAIWRAARRSTLALKGMMDLGSFQNLPKKRRNVDVHTAKVGVLVTLLFI
Bb_op21_297aa   181 VLPMVVMVVCYYAIWRAARRSTLALKSMMDKGSFQKLPKKRRQVDVHTAKIGLLVTMLFI

                   TM VI                               TM VII
                   #############                ######*#############
Bl_op21_295aa   241LSWTPFAIVALIGWAGYGHLI--TPLAGAIPAAIAKAAVIVNYIVYAAMMPDFKRGT
Bf_op21_295aa   241 LSWTPFAIVALIGWAGYGHVL--TPLAGAIPAAIAKMSVVVNYVVYAALMPDFKRGR
Bb_op21_297aa   241 LAWTPFAIVALIGFAGYGHLLTLNPLAGAIPAAIAKGAVVVNFVVYAALMPDFQRGT
```

NOTES-

An asterisk (*) next to gene models denotes that the database model was modified in the current study

1. Numbers in brackets mark the gene location (from the translation initiation to the termination codon)

2. Numbers in brackets represent either an alternative gene model in the database (m), an allele (a) or a gene duplicate due to a mistake in the assembly (d)

3. As concluded from *in silico* analysis and cloning/sequencing experiments in this study

4.‡: Scaffold from v18h27.r3_ref_genome was analyzed

5. + / – denote presence or absence of respective motif in the protein according to ScanProsite analysis. Numbers in brackets mark position of the motif in the *B. floridae* protein sequence, except for the cases of op12b and op13b.

Supplementary file 1. Predicted transcripts and encoded opsin proteins from the three *Branchiostoma* species, their genomic location, structure and alignment of orthologs. Blue and black font in transcripts denote alternating exons. Green, blue and red letters in protein sequences mark the transmembrane domains, the extracellular and the cytoplasmic loops, respectively. The K296 lysine is colored pink. In the alignments, the seven transmembrane domains are marked in Roman numerals, together with the position of K296 (pink star).

Supplementary Table 1. Opsin genes in the *Branchiostoma* genus

		B. lanceolatum	*B. floridae*[1]	*B. belcheri*[2]
C-type opsins	*op1*	MF464463	BK010247 (c-opsin1)	BK010225 (*Amphi*op5)
	op2	MF464464	BK010248	BK010226
	op3	MF464465	BK010249 (c-opsin3)	BK010227 (*Amphi*op4)
	op4	MF464466	BK010250	BK010228
	op5	MF464467	BK010251	BK010229
Neuropsins	*op6*	Not present	BK010252	Not present
	op7	MF464468	BK010253	BK010230
	op8	MF464469	BK010254	BK010231
Go opsins	*op9*	MF464470	BK010255	BK010232 (*Amphi*op2)
	op10	MF464471	BK010256	BK010233
	op11	MF464472	BK010257	BK010234
	op12a	MF464473	BK010258	BK010235 (*Amphi*op1)
	op12b	Not present	Not present	BK010236
	op13a	MF464474	BK010259	BK010237
	op13b	MF464475	Not present	Not present
Peropsins	*op14*	MF464476	BK010260	BK010238 (*Amphi*op3)
Melanopsins	*op15*	MF464477	BK010261	BK010239 (*Amphi*Mop)
Amphiop6	*op16*	MF464478	BK010262	BK010240
	op17a	MF464479	BK010263	Not present
	op17b	MF464480	Not present	Not present
	op18	MF464481	BK010264	BK010241
	op19	MF464482	BK010265	BK010242 (*Amphi*op6)
	op20	MF464483	BK010266	BK010243
	op21	MF464484	BK010267	BK010244
TOTAL		21+1pseudo	21	20

Notes:
Numbering of genes is based on Pantzartzi *et al*

1: Gene names in parentheses based on Vopalensky et al (2012)

2: Gene names in parentheses based on Koyanagi et al. (2002) and Koyanagi et al. (2005)

Supplementary Table 2. Primers used for validation of predicted models and RT-PCR

Primers for validation of *in silico* predicted models

Target	Name		5´ - 3´ sequence
Bf_op1	JP109	F	TGTCTGCTGTGGACGACTCTA
	JP280	F	GGTCTGGTATCCCTGATCTCC
	362-PV	R	GCTGAAGCTTACGTTTTAACCTCGCCAAATTTGTTG
Bf_op2	JP538	F	TCAACTGGGAGTCACGCAC
	364-PV	R	GCTGAAGCTTTTATTTCTCTGCCTCTAATAGGGTGGAAATG
Bf_op3	18-PV_JP	F	TGTGTCCTACATCGTGAC
	Opsin12R	R	CCCAAGCTTCTACTCTGATATAGTGGAGAGTG
Bf_op4	20-PV-RT	F	CAACGACATCTCCTACATCA
	21-PV-RT	R	AGGAAACACGTGATCAGCA
Bf_op5	244-PV	F	CGACGCGTATGGACACCATGGCACCAACAC
	102-PV	R	CTGGATCCTCACAGCGGAGTTCCGAACGGT
Bf_op6	246-PV	F	CGACGCGTATGGCCACCACGCCGGCAG
	JP535A	F	TGGTATGGCTTCGTTGTG
	JP535B	R	ATGACGACAGGGACGGCGGTA
	247-PV	R	GCTGAAGCTTATTCGGACCTGAAGCGCTCGTTC
Bf_op7	31-PV	F	CATCTCAGCGATCTACAG
	JP530	R	TGTATGGAGTCTGCTGTCCAG
	JP548B	R	TCATGTTTCATTGCGGAACC
Bf_op8	35-PV	F	CGTCATCACTTTCTGCTAC
	Opsin13R	R	CCCAAGCTTTTACACCCAGGTGGATGG
Bf_op9	24-PV	F	ATCCTGATGACGCTGTTCTA
	Opsin14R	R	CCCAAGCTTCTAGCAGTCAACAATATACGC

Target	Name		5´ - 3´ sequence
Bf_op10	26-PV-RT	F	AGCATCAAGTGGACAGTAG
	27-PV-RT	R	CCACCATGAACAGTATCAC
Bf_op11	JP520A	F	ATGGACAACTCTACATGTTG
	JP520B	R	CTACACAAGGTTATCTTTGTAG
Bf_op13a	JP519A	F	TGGCCTACACCGTACTGAC
	JP519B	R	TGGCTGTTCCGTATTCAACG
Bf_op14	517A	F	ATGGCATCACTCTGGCAACC
	517B	R	CCGAAGAACATGCCGTTAAAGC
	37-PV-RT	F	AGTACCAGAACCACTTCTCA
	JP525	R	GGCATTGGCGTTCCCCATGG
Bf_op15	JP148	F	CGGGATCCATGGAGGTGACGCCTACC
	183PV	R	GGAATTCTCACAAAACAGTCTCATCATGG
Bf_op16	JP544A	F	AAATATTTCCATGTGCTTGG
	JP545B	R	CTTGAGAGGTCGTCACGA
Bf_op17a	JP545A	F	TAGCGGCGGCTATTGTTG
	JP551D	R	CCATCACGTCGCGACCGAT
Bf_op20	JP554C	F	ACTCGCAAACTGAGGAGACC
	JP554D	R	GTATGGTGAGAGATTTCATCG
Bf_op21	12-PV-RT	F	CCTGACGTTAGCAGTCTTCG
	Opsin9R	R	CCCAAGCTTTTACCTACCTCGCTTGAAGTC

Primers for RT-PCR

Target	Name		5´-3´sequence	Target	Name		5´-3´sequence

Bf_op1	16-PV-RT<br>17-PV-RT	F<br>R	GCTCCTCTTACCTTACCTAC<br>GATGAAGATAGCCACGATG
Bf_op2	JP274<br>JP275	F<br>R	GCTGGGCATAGTGAACAACTC<br>ATGACCGTCAAGGTGATCAAG
Bf_op3	JP278<br>JP279	F<br>R	CCCTGATGGTGACATGTTTCT<br>GCACACCAGGAAACAGATGAT
Bf_op4	JP276<br>JP277	F<br>R	TACGGCTTCACCAACAGTTTC<br>GGGAGTAGATCCAGACGAAGG
Bf_op5	JP103<br>JP104	F<br>R	GCATGGTGCTACTCGTTCTTC<br>GTTCGGCACCTCATCTTCAAC
Bf_op6	JP535A<br>JP535B	F<br>R	TGGTATGGCTTCGTTGTG<br>ATGACGACAGGGACGGCGGTA
Bf_op7	31-PV-RT<br>32-PV-RT	F<br>R	CATCTCAGCGATCTACAG<br>GAGTGGTACTGCATCTAC
Bf_op8	JP290<br>JP291	F<br>R	GCTCGGCTCATATGTTCTCTG<br>CATGAACCTGAAAAAGGCGTA
Bf_op9	JP111<br>JP112	F<br>R	AGCATCCACAGGTACATCACC<br>CCACAGTGGTACCGTGAAAGT
Bf_op10	JP113<br>JP114	F<br>R	TGTCCATGAGCAGTCTCAACA<br>CGACATCTCGGATGTGTTCTC
Bf_op11	JP520C<br>JP520E	F<br>R	GATTGACCCAGAGCTTCAC<br>CTTCCTTTTGTCCTTCTTCAC
Bf_op12a	JP89<br>JP90	F<br>R	CGTCTACCGCTACATCGTCAT<br>GCCGCAGTGTAGATTGTCTCT
Bf_op13a	JP519A<br>JP519B	F<br>R	TGGCCTACACCGTACTGAC<br>TGGCTGTTCCGTATTCAACG
Bf_op14	37-PV-RT<br>38-PV-RT	F<br>R	AGTACCAGAACCACTTCTCA<br>TTCCTCCAGCTCCACATCAG
Bf_op15	JP101<br>JP102	F<br>R	GTTGTTCGGATGTCTGTCCAT<br>GGCGTCATGTAGTCGAAAGTG
Bf_op16	JP97<br>JP98	F<br>R	GTCACTCAAGACCCACCAGAG<br>GGGCATCACAGGTCTTACACT
Bf_op17a	6-PV-RT<br>7-PV-RT	F<br>R	CACTACCTGGTGCTGAAC<br>TCATCACACTGACACTGAAG
Bf_op18	JP546A<br>JP546B	F<br>R	ATGGACTTCTTTCTCGACAATT<br>GCCACCAGATAGTTCTTAGG
Bf_op19	JP87<br>JP88	F<br>R	GTGGTCTGCCTGGTGTACAGT<br>CTGGGTGATGGTGACGTATCT
Bf_op20	JP554C<br>JP554D	F<br>R	ACTCGCAAACTGAGGAGACC<br>GTATGGTGAGAGATTTCATCG
Bf_op21	JP99<br>JP100	F<br>R	GGTCCATGCAGAAATACCAGA<br>GTCGACATTCCTTCGCTTTTT

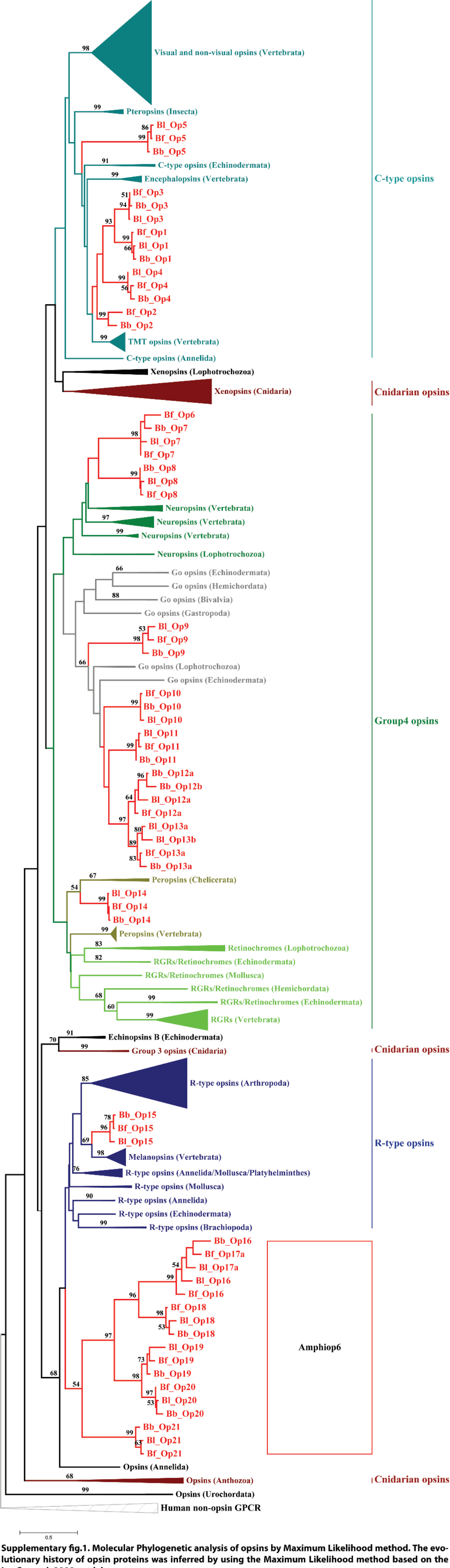

Supplementary fig.1. Molecular Phylogenetic analysis of opsins by Maximum Likelihood method. The evolutionary history of opsin proteins was inferred by using the Maximum Likelihood method based on the Le_Gascuel_2008 model.

The tree with the highest log likelihood is shown. Bootstrap values are shown (only values>50) either at the nodes or above the branches in the case of non-expanded subgroups (Neuropsins). A discrete Gamma distribution was used to model evolutionary rate differences among sites (2 categories). The tree is drawn to scale, with branch lengths measured in the number of substitutions per site. The analysis involved 827 amino acid sequences. There were a total of 419 positions in the final dataset (third cytoplasmic loop was excluded).

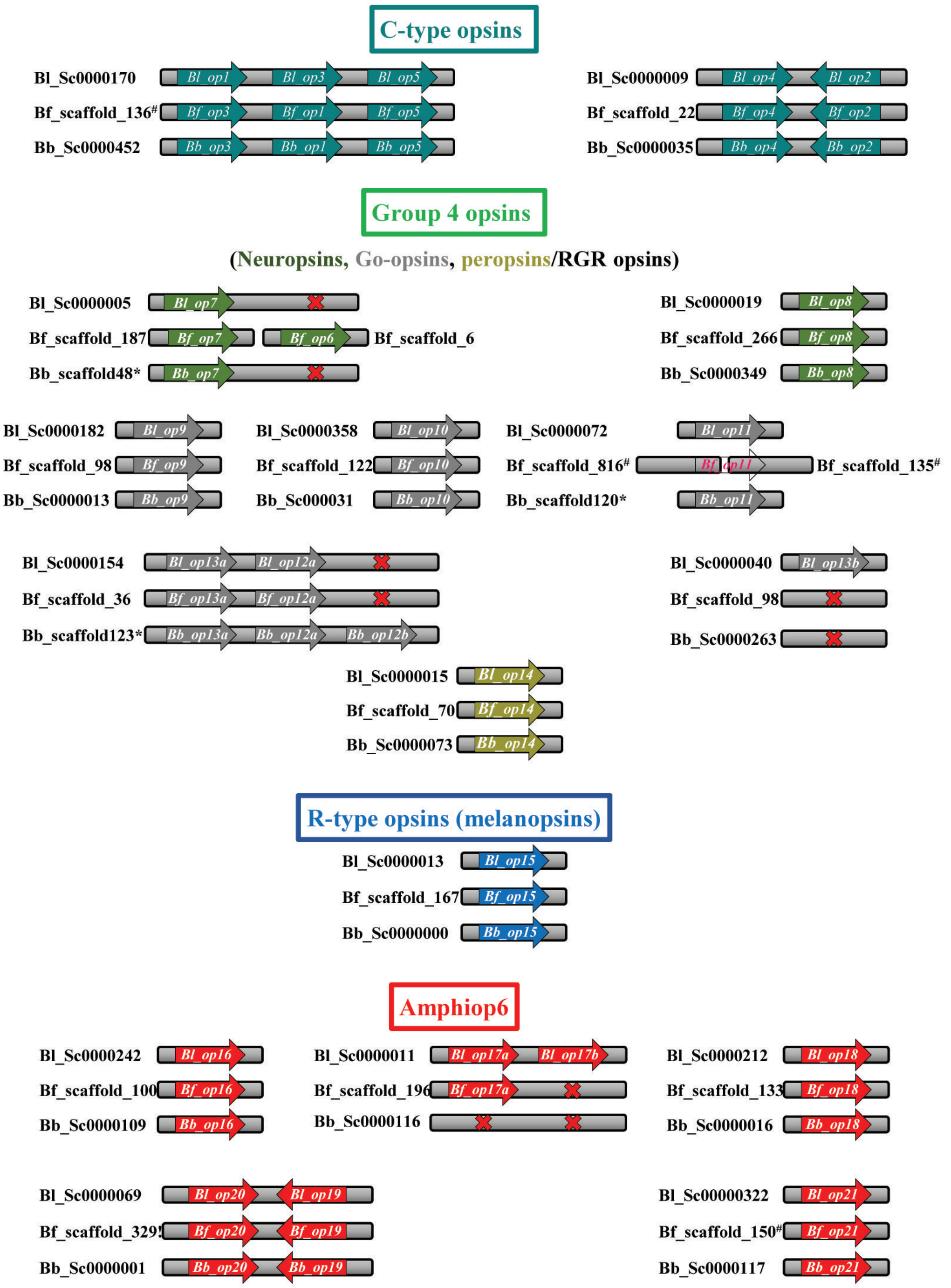

Supplementary Fig.2. Opsin containing genomic loci from *Branchiostoma* genus.

The genomic loci from *Branchiostoma lanceolatum* (Bl), *B. floridae* (Bf) and *B. belcheri* (Bb) containing opsin genes or lacking opsin genes (red x) but exhibiting synteny conservation to either of the other two respective opsin loci are depicted. Scaffold numbers for *B. floridae* correspond to v2.0 genome assembly in NCBI; # scaffolds correspond to the v1.0 genome assembly in JGI; † problem with genome assembly, see Supplementary File 1. Scaffold numbers for *B. belcheri* correspond to HapV2 genome assembly; * scaffolds refer to the r3refgenome assembly. Groups are colored based on their phylogenetic position (see supplementary Figure 1)

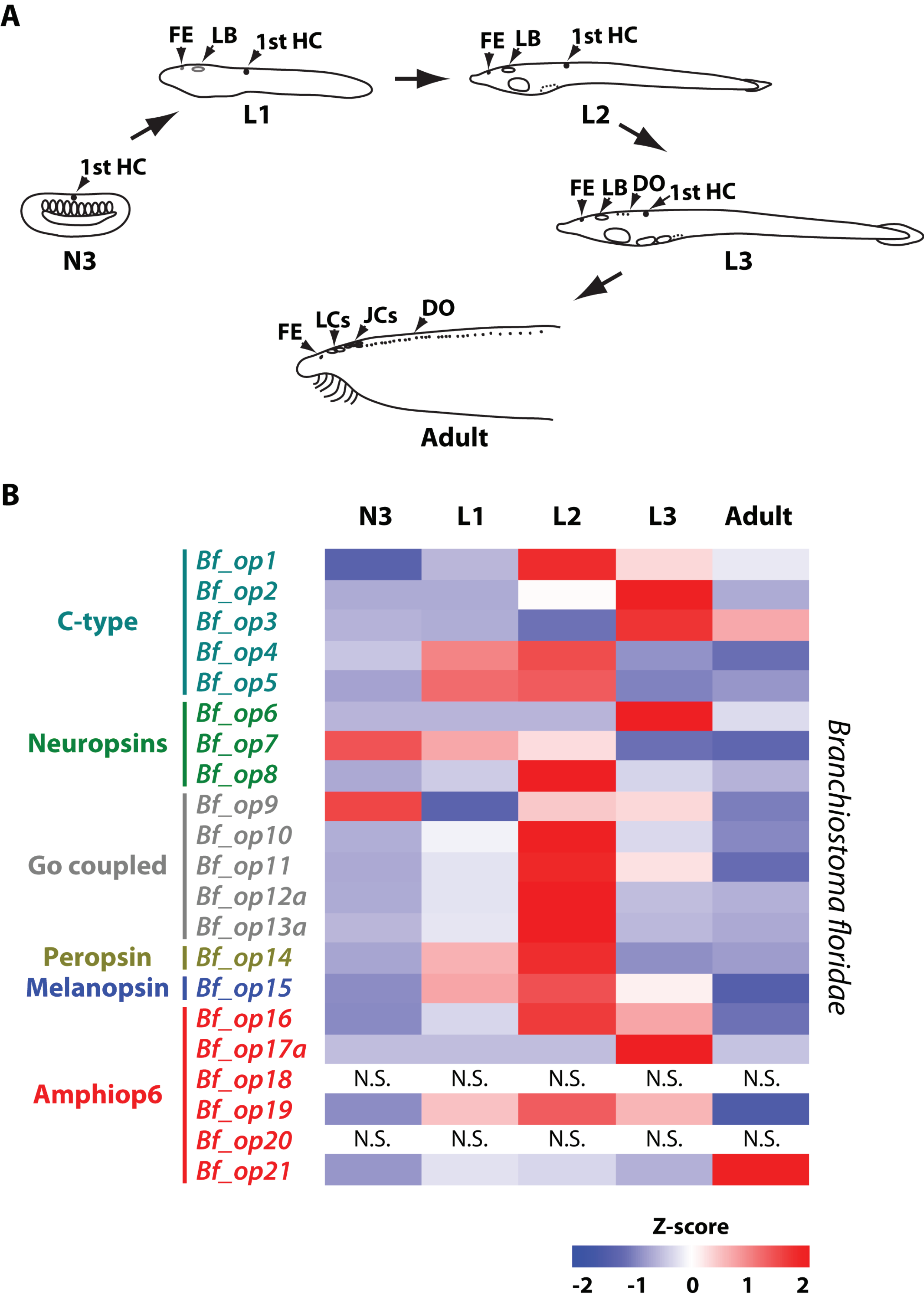

Supplementary Fig.3. mRNA expression levels of individual B. floridae opsins across different developmental stages.

(A) Schematic drawing of developmental stages (N3, L1, L2, L3, adult), in which detection of opsin genes expression was performed. Staging was determined according to Hirakow and Kajita (1994) (see Materials and Methods). 1st HC: 1st Hesse cell, FE: frontal eye, LB: lamellar body, DO: dorsal ocelli, JCs: Joseph cells. (B) Heat map displaying expression of opsin genes across different developmental stages. Opsin genes expression was detected by qRT-PCR and normalized to expression of TBP (B. floridae). Each row represents particular opsin gene expression in various developmental stages. Blue color represents expression below row average, white color represents average row expression, red color expression above row

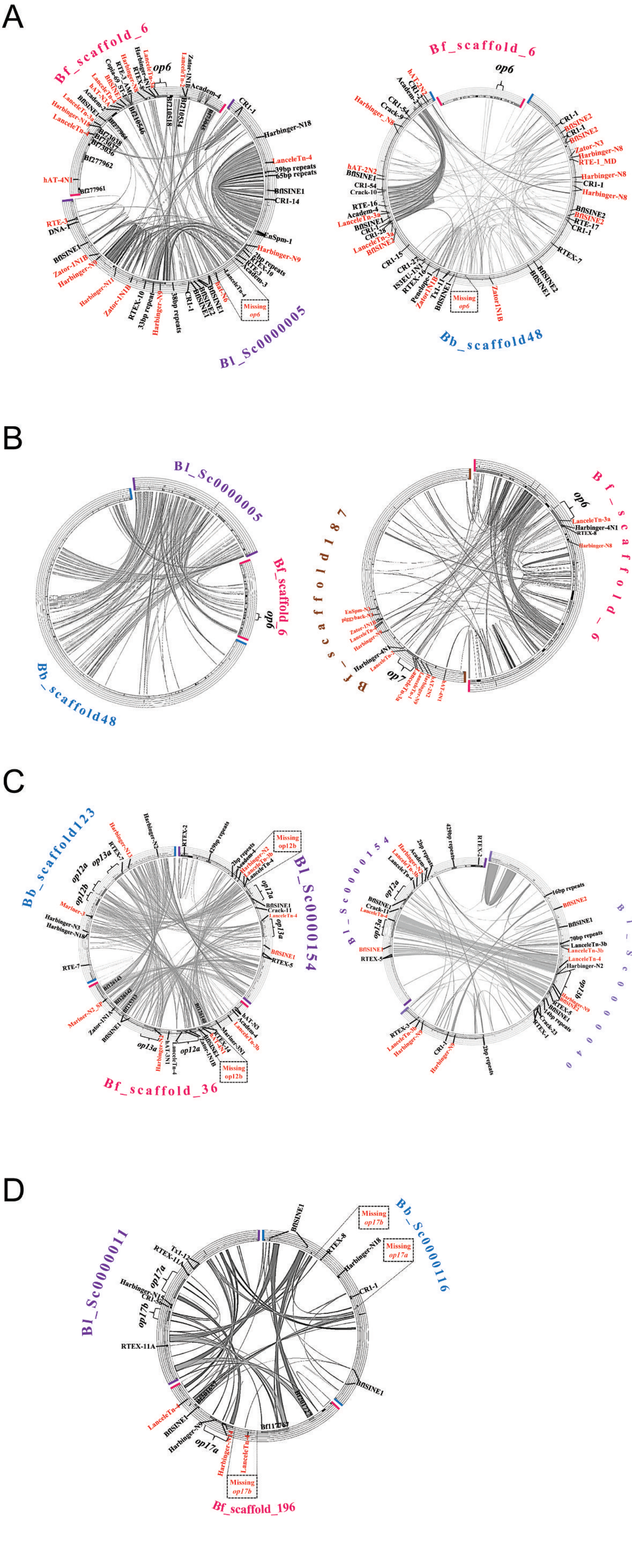

Supplementary Figure 4. Transposable elements and Branchiostoma opsin genes.

(A) Comparison of genomic regions of Bf_scaffold_6 with Bl_Sc0000005 (left) and Bb_scaffold48 (right), same as in fig.1. (B) Comparison of the op6-containing Bf_scaffold_6 with Bl_Sc0000005 and Bb_scaffold48 (left) and with Bf_scaffold_187 (right). Similarity between B. lanceolatum and B. belcheri scaffolds seems to be larger than of either of these with B. floridae. For B. floridae, similarity is observed in the genic region of op6 and op7 but not so much in the flanking regions. (C) Left: Comparison of Bl_Sc0000154 with Bf_scaffold_36 and Bb_scaffold_23, as in fig. 2. Right: Comparison of B. lanceolatum scaffolds bearing opsins op13a (Sc0000154) and op13b (Sc0000040). (D) Comparison of the op17a-containing Bf_scaffold_196 with Bl_Sc0000011 and Bb_Sc0000116, as in fig.3. Simple tandem repeats and various transposable elements (TE) are marked in red and black letters (complete and partial copies based on the Rep-Base database). Predicted B. floridae gene models are listed in the internal part of the scaffolds.
